# Palladium-Catalyzed Carbonylative Sonogashira Coupling of Aliphatic Alkynes and Aryl Thianthrenium Salts to Alkynones and Furanones

**DOI:** 10.1021/acs.orglett.4c03519

**Published:** 2024-12-09

**Authors:** Yan-Hua Zhao, Xing-Wei Gu, Xiao-Feng Wu

**Affiliations:** † Leibniz-Institut für Katalyse e.V., Albert-Einstein-Straße 29a, 18059 Rostock, Germany; # Dalian National Laboratory for Clean Energy, Dalian Institute of Chemical Physics, Chinese Academy of Sciences, Dalian 116023, Liaoning, China

## Abstract

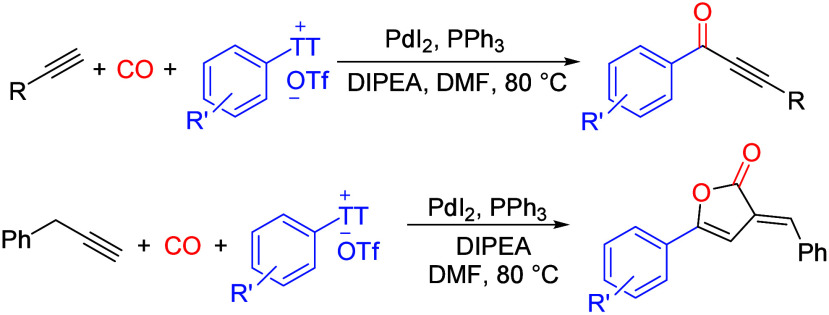

Herein, we developed a mild and efficient palladium-catalyzed carbonylative Sonogashira coupling of aryl thianthrenium salts with aliphatic alkynes and benzyl acetylene toward alkynones and furanones. Various desired products were prepared in good yields with broad functional group tolerance including the bromide group. In the case of using benzyl acetylene, the corresponding furanones can be obtained in good yields under the same conditions with two molecules of CO inserted.

Alkynones are an important class of compounds found in many bioactive molecules,^1^ natural products,^2^ and pharmaceuticals.^3^ They are the key intermediates in synthesizing heterocyclic compounds^4^ such as furans,^5^ pyrazoles,^6^ pyrroles,^7^ and flavonoids.^8^ Hence, various synthetic procedures were developed for their preparation. Traditionally, the reaction of alkynyl organometallic reagents^9^ or terminal alkynes^10^ with acyl chlorides plays an important role in the preparation of alkynones. However, besides storage issues, the requirement of an inert atmosphere protection and the use of dry solvents limited their applications. Moreover, the method often suffers from a narrow functional group tolerance and poor substrate stability. To solve these problems, alternative methods are highly desired, such as transition metal-catalyzed carbonylative Sonogashira reaction.^11^

Carbon monoxide (CO), as a cheap and easily available source of C1, has been recognized as an important synthon with the development of carbonylation chemistry.^10d,12^ In 1981, Kobayashi and Tanaka reported the preparation of acetylenic ketones by the palladium-catalyzed carbonylation Sonogashira coupling reaction of terminal alkynes with organic halides first.^13^ Afterward, the substrates were expanded aryl triflates,^14^ and aryl triazenes.^15^ In 2011, Lee’s group reported the synthesis of alkynyl ketones by the reaction of aryl iodides with alkynyl carboxylic acids in the presence of a Pd/Cu catalyst under a CO atmosphere.^16^ In 2017, Wu’s group reported a carbonylation cross-coupling method for aryl diazonium salts with terminal alkynes by using formic acid as the CO source.^17^ In the same year, El Ali’s group reported the carbonylation Sonogashira coupling reaction of aryl alkynes, alkyl alkynes and dialkynes with aryl iodides in the presence of (N-heterocyclic carbene) Pd(pyridine)Br_2_ complex.^18^ Recently, Lei’s group achieved a smooth synthesis of acetylenic ketones from arylhydrazines and terminal alkynes by electrochemical Pd-catalyzed oxidative Sonogashira carbonylation.^19^

Additionally, furanones are vital structural motifs^20^ in a variety of pharmaceutical molecules and natural products with a range of biological activities such as cardiotonic, anticancer, analgesic, antituberculosis, anti-inflammatory, antimicrobial, antimalarial, and antiviral activities.^21^ Presently, medicines incorporating a furanone substrate are even commercially available, e.g., narthogenin, ascorbic acid, butalactin, basidalin, and rofecoxib. Thus, far, there have been several reports on the synthesis of furanones, by transition metal-catalyzed cyclization of alkyne substrates.^22^

In recent years, thianthrenes have been brought to the attention of scientists inspired by the studies from groups including Shine, Ritter, and others.^23^ Thianthrenium salts have a higher activity and better chemoselectivity than aryl halides. In addition, thianthrenium salts are reactive electrophilic reagents that are readily available from inexpensive and readily available aromatic hydrocarbons. It was demonstrated in previous work by our group that the thianthrenium salt can undergo a carbonylation cross-coupling reaction with aryl alkynes in the presence of a palladium catalyst to give the corresponding alkynes in excellent yields.^24^ Unfortunately, aliphatic alkynes were failed in this catalytic system. In order to solve this challenge, we restudied this transformation with aliphatic alkynes. Herein, we report our new results on palladium-catalyzed carbonylative Sonogashira coupling of aliphatic alkynes with thianthrene salts. Notably, in the case of using benzyl acetylene, the corresponding furanones can be obtained in good yields under the same conditions with two molecules of CO inserted.

Initially, we started with readily available aryl thianthrenium salt **2a** and heptane **1a** as the model substrates to establish this reaction, and the target product **3a** was obtained (Table 1). After a systematic investigation of the reaction parameters, we determined the optimal conditions: alkyl alkyne **1a** (1.0 equiv), aryl thianthrenium salt **2a** (1.5 equiv), PdI_2_ (2 mol %), PPh_3_ (6 mol %), DIPEA (2.0 equiv), DMF (0.1 mol L-1), under CO (10 bar) atmosphere, at 80 °C for 15 h, and the corresponding alkenone **3a** was successfully obtained with a GC yield of 78% (72% isolated yield, Table 1, entry 1). In variation experiments, the reaction did not proceed in the absence of a ligand, catalyst, or base (Table 1, entries 2–4). Different bases were then examined, and only 56% of the product was detected when DABCO was used as the base (Table 1, entry 5). No product could be detected when inorganic bases (e.g., Cs_2_CO_3_ or K_3_PO_4_) were tested (Table 1, entries 6–7). Next, different catalyst precursors (e.g., Pd(OAc)_2_, PdBr_2_, (cinnamyl)_2_Pd_2_Cl_2_) were checked in the reaction, and the yield of the desired product decreased (Table 1, entries 8–10). Screening of other phosphine ligands showed that monodentate phosphine ligands (e.g., PCy_3_ or BuPAd_2_) exhibited good reactivity (Table 1, entries 11–12). In contrast, bidentate phosphine ligand BINAP was ineffective in promoting the reaction, and only a trace amount of the desired product was detected (Table 1, entry 13). Among the solvents tested, 61–62% of the target product was obtained using DCE and DMSO instead of DMF (Table 1, entries 14–15). The corresponding acetylenic ketone was formed in 40% yield when acetonitrile was used as the solvent (Table 1, entry 16). Moreover, modifying the reaction concentration resulted in a decreased yield (Table 1, entry 17). When the amount of arylthianthrene salt **2a** was reduced, a 70% yield of the target product was achieved. In addition, the reaction was inhibited by the addition of CuI as an additive (Table 1, entry 19).

**Table 1:**
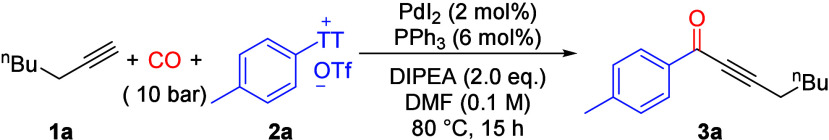
Optimization of the Reaction Conditions^a^

Entry	Variation from the Standard conditions	Yield (%)^b^
1	None	78 (72)^c^
2	No ligand	n.o.
3	No catalyst	n.o.
4	No base	n.o.
5	DABCO instead DIPEA	56
6	Cs_2_CO_3_ instead of DIPEA	n.o.
7	K_3_PO_4_ instead of DIPEA	n.o.
8	Pd(OAc)_2_ instead of PdI_2_	65
9	PdBr_2_ instead of PdI_2_	65
10	(cinnamyl)_2_Pd_2_Cl_2_ instead of PdI_2_	61
11	PCy_3_ instead of PPh_3_	39
12	BuPAd_2_ instead of PPh_3_	63
13	BINAP instead of PPh_3_	trace
14	DCE as solvent	61
15	DMSO as solvent	62
16	CH_3_CN as solvent	40
17	0.2 mol L^–1^ DMF	66
18	1.0 equiv. **2a**	70
19	CuI (10 mol %) as additive	trace

a The reaction was conducted using **1a** (0.2 mmol), **2a** (x mmol), catalyst (2 mol %), ligand (6 mol %), base (2.0 equiv), CO (10 bar), 80 °C, 15 h.

b Determined by GC with hexadecane as internal standard.

c Isolated yield is shown in parentheses.

Under the optimal reaction conditions, we initially examined thianthrenium salts containing different substituents. Methyl, methoxy, and deuterated thianthrenium obtained the corresponding alkynes in high yields (Table 2, entries 1–3). Disubstituted salts can afford the target product in 71% yield (Table 2, entry 10). To investigate the chemoselectivity of the reaction, halogen-substituted thianthrenium salts were tested, and the corresponding products were obtained in moderate yields (Table 2, entries 4–6). Delightfully, the disubstituted substrates containing C(sp^2^)–X bonds (X = F, Cl, and Br) can also be successfully converted into the thianthrenium desired alkynones in good yields (Table 2, entries 7–9). However, the reaction become messy when iodide containing substrate was tested. In addition, halogen-modified trisubstituted aryl thianthrene salts were also compatible with this reaction system, yielding the targeted product in 60% yield (Table 2, entry 11). This indicates that the catalytic system has good chemoselectivity and provides the possibility of further structural modifications.

**Table 2:**
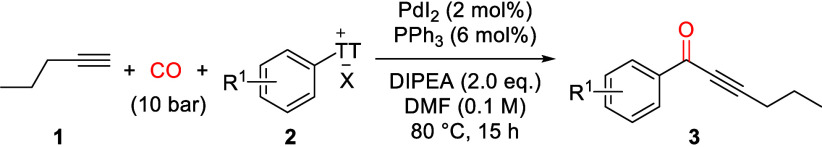

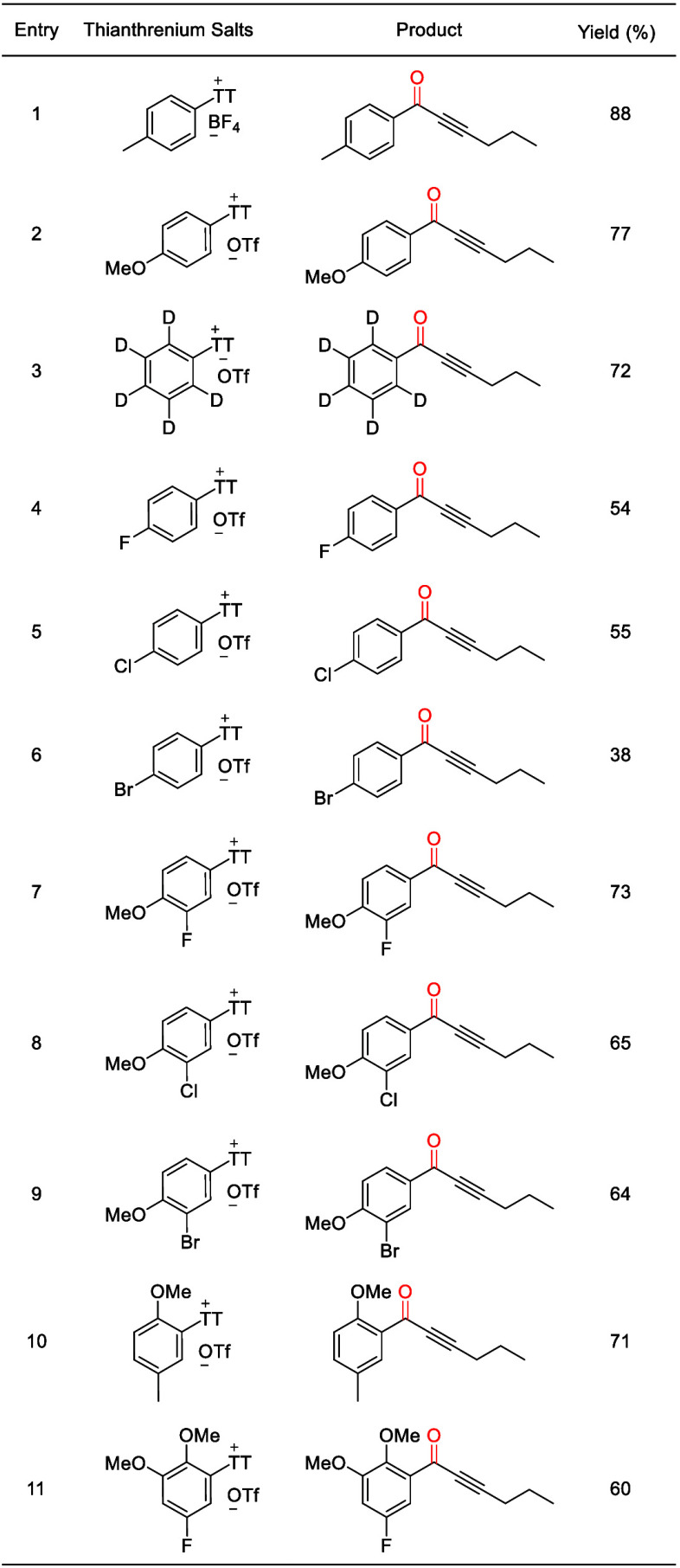
Substrate Scope of Aryl Thianthrenium Salts^a^

a Reaction conditions: **1** (0.2 mmol), **2** (0.3 mmol), PdI_2_ (2 mol %), PPh_3_ (6 mol %), DIPEA (2.0 equiv), CO (10 bar), DMF (0.1 M), 80 °C, 15 h. yields of isolated products are shown. TT = thianthrene.

Subsequently, we examined several aliphatic alkynes (Table 3). Simple straight-chain alkynes showed good reactivity to the corresponding products in good yields (Table 3, entries 1–4, 7). Cyclohexylacetylene and cyclopropylacetylene were also used as substrates and transformed into the corresponding acetylenic ketones in high yields (Table 3, entries 5–6). Here, no compounds related with ring opening of the cyclopropane group could be detected from the reaction mixture. In addition, phenylacetylene was also amenable to these reaction conditions, and the target product was obtained in good yield (Table 3, entry 8). Notably, benzylacetylene was used as a substrate in this catalytic system as well, and the corresponding furanone was generated in good yield with two molecules installed (Table 4, entry 1). Under optimal conditions, the reaction of benzylacetylene with aryl thianthrenes containing different substituents all led to differently substituted furanones without problem (Table 4, entries 2–6).

**Table 3:**
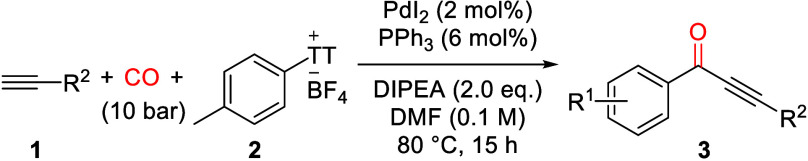

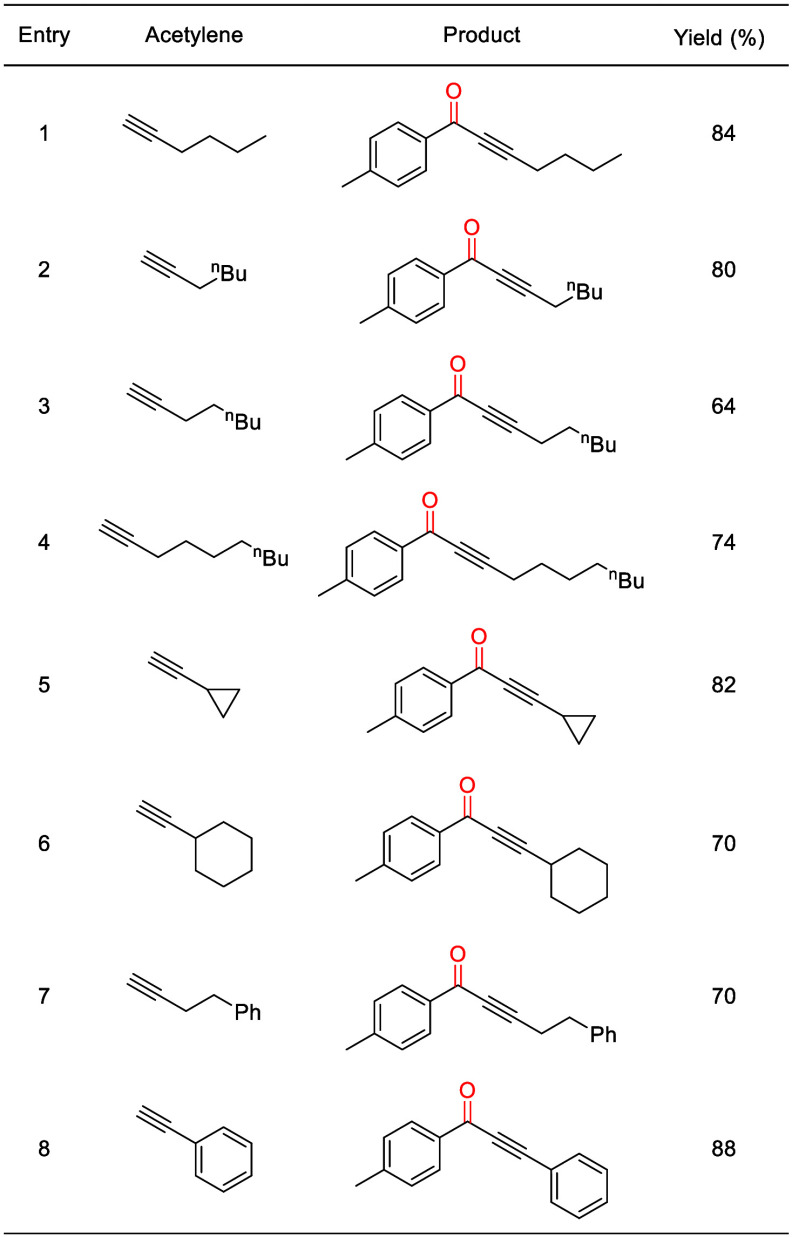
Substrate Scope of Acetylene^a^

a Reaction conditions: **1** (0.2 mmol), **2** (0.3 mmol), PdI_2_ (2 mol %), PPh_3_ (6 mol %), DIPEA (2.0 equiv), CO (10 bar), DMF (0.1 M), 80 °C, 15 h. yields of isolated products are shown. TT = thianthrene.

**Table 4:**
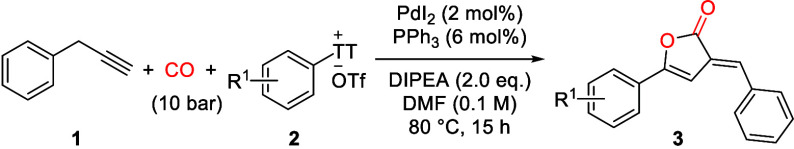

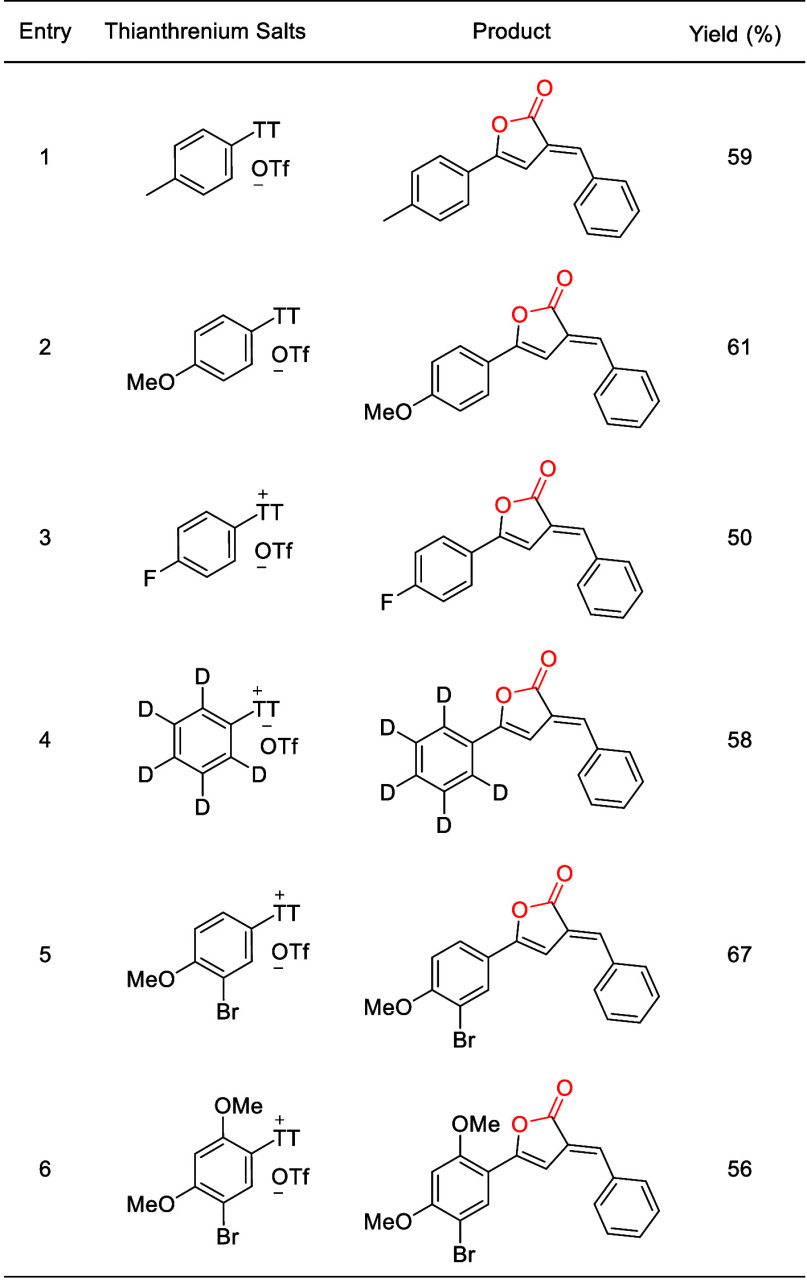
Substrate Scope of Acetylene^a^

a Reaction conditions: **1** (0.2 mmol), **2** (0.3 mmol), PdI_2_ (2 mol %), PPh_3_ (6 mol %), DIPEA (2.0 equiv), CO (10 bar), DMF (0.1 M), 80 °C, 15 h. yields of isolated products are shown. TT = thianthrene.

We performed several mechanistic experiments to illustrate the reaction mechanism. Adding TEMPO (2 equiv) or BHT (1–3 equiv) was added to the reaction under standard conditions, the reaction still progressed smoothly and provided the corresponding acetylenic ketones in 69–71% yields (Scheme 1a). In addition, 1,1-diphenylethylene (1,1-DPE) was added to the standard reaction mixture, which provided the desired acetylenic ketone **3a** in 65% yield (Scheme 1b). Therefore, we can rule out the possibility of free radical intermediate involvement.

**Scheme 1:**
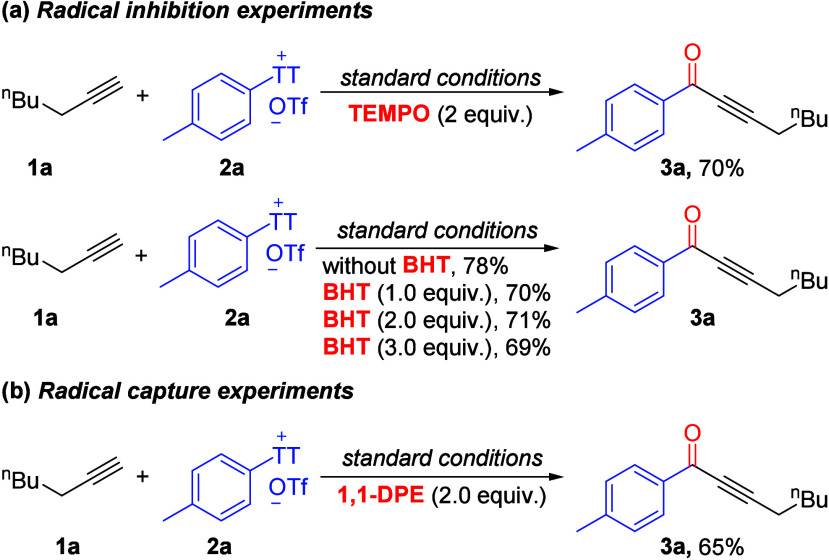
Control Experiments

A possible reaction mechanism was introduced based on the above results and related reports^25^ (Scheme 2). Initially, the active Pd(0) complex **A** was produced from the palladium precursor, which generated the palladium complex **B** by oxidative addition with an aryl thianthrenium salt. After that, the CO coordinates and inserts into complex **B** to produce intermediate **C**. Then terminal alkynes undergo a ligand exchange reaction with intermediate **C** to generate alkynyl palladium complex **D**. Finally, reductive elimination from complex **D** to give the target product and meanwhile regenerate complex **A** for the next catalytic cycle (cycle I). In the case of benzylacetylene, the produced alkynone will isomerize to the corresponding allene in the presence of a base. Then it is activated by palladium to give intermediate E which will give complex F after the coordination and insertion of the second molecule of CO. Finally, reductive elimination will release the isolated furanone product and regenerate palladium for the next catalytic cycle (cycle II).

**Scheme 2:**
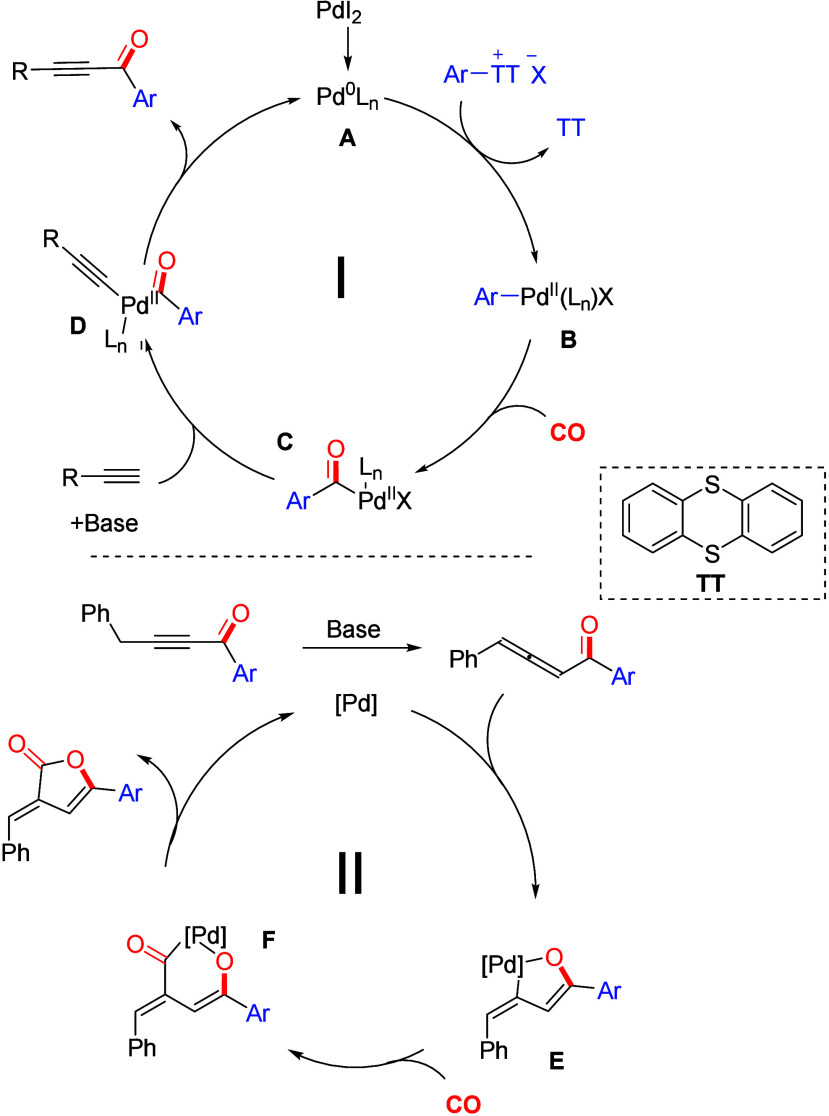
Proposed Mechanism

In conclusion, we developed a new palladium-catalyzed carbonylative Sonogashira coupling reaction of aliphatic alkynes and benzylacetylenes with aryl thianthrenium salts. In general, moderate to good yields of the desired alkynones can be produced selectively. Additionally, good yields of furanones can be obtained using benzylacetylene as the feedstock. Among the obtained products, a range of halogen-containing acetylenic ketones and furanones were formed, which also provide opportunities for further transformations.

## Data Availability

The data underlying this study are available in the published article and its Supporting Information.

## Supporting Information Available

General comments, general procedure, optimization details, analytic data, and NMR spectra (PDF)
