# Supplementary material for: Palladium-Catalyzed Carbonylative Sonogashira Coupling of Aliphatic Alkynes and Aryl Thianthrenium Salts to Alkynones and Furanones
Source: Org Lett. 2024 Dec 9;26(50):10702–7. doi: 10.1021/acs.orglett.4c03519 (PMC11667721; doi:10.1021/acs.orglett.4c03519)
Supplement: Supplementary file 1 — ol4c03519_si_001.pdf [file ol4c03519_si_001.pdf]

Supporting Information

Palladium-Catalyzed Carbonylative Sonogashira Coupling of Aliphatic Alkynes and Aryl Thianthrenium Salts to Alkynones and Furanones

Yan-Hua Zhao,[†] Xing-Wei Gu,[†] Xiao-Feng Wu[*†,#]

[†]Leibniz-Institut für Katalyse e.V., Albert-Einstein-Straße 29a, 18059 Rostock, Germany

Dalian National Laboratory for Clean Energy, Dalian Institute of Chemical Physics, Chinese Academy of Sciences, Dalian 116023, Liaoning, China, E-mail: xwu2020@dicp.ac.cn.

Contents

1. General Experimental S2
2. General procedure for the synthesis of thianthrenium salt. S2
3. General Procedure for the carbonylation. S4
4. Characterization and procedure of the products S4
5. Control experiments S11
6. Reference S12
7. NMR spectra of products S14

1. General Experimental

Reagents and solvents: Unless otherwise noted, the chemicals were commercially available from Sigma-Aldrich, TCI or Alfa Aesar and were used without further purification. Dioxane bought from Alfa Aesar, HPLC grade, 99% min, packaged under argon in resealable ChemSeal bottles. The reaction does not require the glovebox.

Purification: The products were isolated from the reaction mixture by column chromatography on silica gel 60, 0.063-0.2 mm, 70-230 mesh (Merck). Gradient flash chromatography was conducted eluting with PE/EA, PE refers to pentane and EA refers to ethyl acetate, they were listed as volume/volume ratios.

Data collection: GC-yields were calculated using hexadecane as internal standard. GC analysis was performed on an Agilent HP-7890A instrument with FID detector and HP-5 capillary column (polydimethylsiloxane with 5% phenyl groups, 30 m, 0.32 mm i.d., 0.25 µm film thickness) using argon as carrier gas. High resolution mass spectra (HRMS) were recorded on Agilent 6210. NMR spectra were recorded on Bruker Avance 300 and Bruker ARX 400 spectrometers. Chemical shifts (ppm) are given relative to solvent: references for $CDCl_3$ were 7.26 ppm (^{1}H NMR) and 77.00 ppm (^{13}C NMR). All measurements were carried out at room temperature unless otherwise stated.

2. General procedure for the synthesis of aryl sulfonium salts.

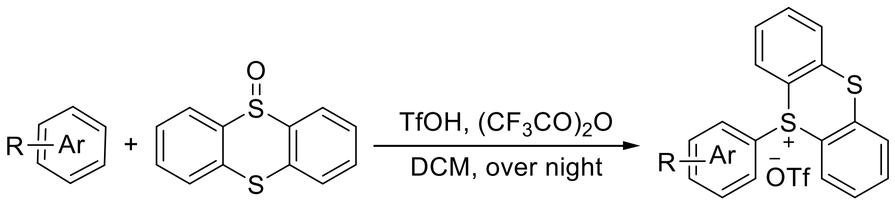

Aryl thianthrenium triflates were prepared by known literature procedure.[1] Thianthrene S-oxide (1.1 equiv.), arene (1 equiv.) and DCM (0.2M) were added to a 100 mL Schlenk tube under a nitrogen atmosphere, equipped with a magnetic stir bar. The reaction mixture was then cooled to -40 °C and stirred at this temperature. Tf_2O (1.2 equiv.) was added dropwise. The reaction mixture was stirred at -40 °C for 30 min, and then allowed to stir at room temperature for 12 h, neutralized by a saturated aqueous $NaHCO_3$ solution, and extracted with DCM. The combined organic layers were dried over anhydrous Na_2SO_4 and concentrated to dryness under reduced pressure. The crude product was purified by crystallization from DCM / diethyl ether system to afford the aryl thianthrenium triflate.

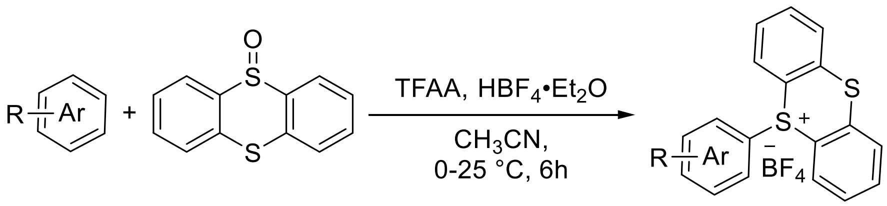

The tetrafluoroborate thianthrenium salts were synthesized according to the literature procedure.[2] Under ambient atmosphere, a 100.0 mL round-bottom flask equipped with a magnetic stir bar was charged with arene (1.0 equiv.) and MeCN (0.44 M). Trifluoroacetic anhydride (2.0 equiv.) was added at ambient temperature while stirring. After cooling to 0°C, thianthrene S-oxide (1.0 equiv.) was added in one portion, followed by the addition of $HBF_4{\cdot}OEt_2$ (2.2 equiv.) in one portion. The mixture was stirred at 0 °C for 1 h, then at ambient temperature for 5 h, neutralized by a saturated aqueous $NaHCO_3$ solution. The reaction mixture diluted with DCM and the layers were separated.

The organic phase was washed with aqueous $NaBF_4$ solution (10%), and with water. The organic phase was dried over Na2SO4, filtered, and the solvent was removed under reduced pressure. The residue was purified by chromatography on silica gel eluting with DCM/MeOH (v/v) to afford products.

3. General Procedure for the carbonylation.

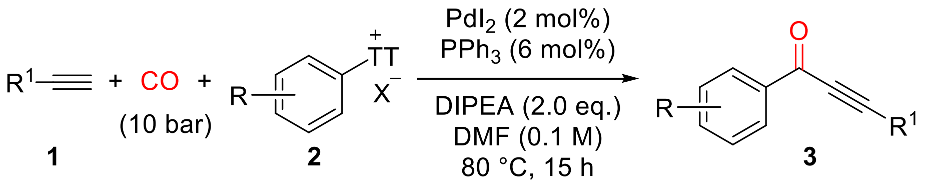

A 4 mL snap vial was charged with PdI_2 (0.004 mmol, 2 mol %), PPh_3 (0.012 mmol, 6 mol%), aryl sulfonium salts (0.3 mmol, 1.5 equiv.) and closed with a rubber-based septum. The vial was evacuated and backfilled with argon. Degassed DMF (2.0 mL), DIPEA (0.4 mmol, 2.0 equiv.), and terminal alkynes (0.2 mmol, 1.0 equiv.) were added via syringe. The vial was then connected to atmosphere with a cannula and transferred into a 300 mL Parr 4560 series autoclave, under argon counterflow. The closed autoclave was flushed three times with nitrogen (~ 5 bar), three times with CO (~ 5 bar), and 10 bar of carbon monoxide (measured by pressure meter) was charged. The autoclave was then placed into an aluminum block on a magnetic stirrer. The reaction mixture was stirred (500 rpm) at 80 ℃ 15 h. The crude product was purified by silica gel chromatography (pentane/EA) to afford the corresponding product.

1 mmol scale: a 12 mL snap vial was charged with PdI_2 (2 mol %), PPh_3 (6 mol%), aryl sulfonium salt 2a (1.5 equiv.) and closed with a rubber-based septum. The vial was evacuated and backfilled with argon. Degassed DMF (6.0 mL), DIPEA (2.0 equiv.), and terminal alkyne 1a (1 mmol, 1.0 equiv.) were added via syringe. The vial was then connected to atmosphere with a cannula and transferred into a 300 mL Parr 4560 series autoclave, under argon counterflow. The closed autoclave was flushed three times with nitrogen (~ 5 bar), three times with CO (~ 5 bar), and 10 bar of carbon monoxide (measured by pressure meter) was charged. The autoclave was then placed into an aluminum block on a magnetic stirrer. The reaction mixture was stirred (500 rpm) at 80 ℃ 15 h. The crude product was purified by silica gel chromatography (pentane/EA) to afford the corresponding product 3a in 85% yield (158.1 mg).

4. Characterization and procedure of the products

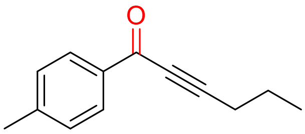

1-(*p*-tolyl) hex-2-yn-1-one[3]

Yellow oil (32.8mg, 88% yield), purified by column chromatography (SiO_2, Pentane/EA= 50:1).

^{1}H NMR (400 MHz, CDCl3) δ 7.99 – 7.92 (m, 2H), 7.23 – 7.15 (m, 2H), 2.40 (t, *J* = 7.0 Hz, 2H), 2.35 (s, 3H), 1.64 (dt, *J* = 14.5, 7.3 Hz, 2H), 1.01 (t, J = 7.4 Hz, 3H).

^{13}C NMR (101 MHz, $CDCl_3$) δ 178.0, 144.9, 134.6, 129.6, 129.2, 96.1, 79.8, 21.7, 21.4, 21.1, 13.6.

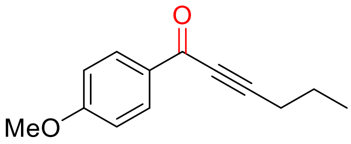

1-(4-methoxyphenyl) hex-2-yn-1-one

Yellow oil (31.1 mg, 77% yield), purified by column chromatography (SiO_2, Pentane/EA= 50:1).

^{1}H NMR (300 MHz, $CDCl_3$) δ 8.09 – 7.98 (m, 2H), 6.92 – 6.81 (m, 2H), 3.80 (s, 3H), 2.39 (t, *J* = 7.0 Hz, 2H), 1.71 – 1.53 (m, 2H), 1.00 (t, *J* = 7.4 Hz, 3H).

^{13}C NMR (75 MHz, $CDCl_3$) δ 176.9, 164.2, 131.8, 130.3, 113.7, 95.7, 79.7, 55.5, 21.4, 21.1, 13.6.

HRMS (ESI-TOF): m/z calcd. for $C_{13}H_{15}O_2$ [M+H $^+$] 203.1067, found 203.1071.

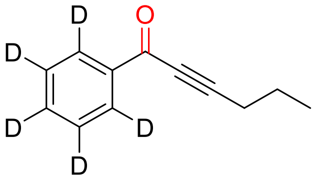

1-(phenyl-*d*$_5$) hex-2-yn-1-one

Yellow oil (25.7 mg, 72% yield), purified by column chromatography (SiO_2, Pentane/EA= 50:1).

^{1}H NMR (400 MHz, $CDCl_3$) δ 2.41 (t, *J* = 7.0 Hz, 2H), 1.64 (h, *J* = 7.3 Hz, 2H), 1.01 (t, *J* = 7.4 Hz, 3H).

^{13}C NMR (101 MHz, $CDCl_3$) δ 178.2, 136.7, 133.2 (d, *J* = 24.6 Hz), 129.4 – 128.8 (m), 128.3 – 127.5 (m), 96.6, 79.8, 21.3, 21.1, 13.6.

HRMS (ESI-TOF): m/z calcd. for $C_{12}H_8D_5O$ [M+H $^+$] 178.1275, found 178.1270.

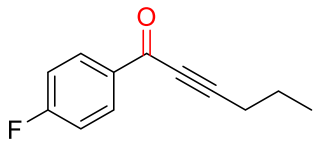

1-(4-fluorophenyl) hex-2-yn-1-one[4]

Yellow oil (20.7 mg, 54% yield), purified by column chromatography (SiO_2, Pentane/EA= 50:1).

^{1}H NMR (400 MHz, $CDCl_3$) δ 8.20 – 8.13 (m, 2H), 7.18 – 7.10 (m, 2H), 2.48 (t, *J* = 7.0 Hz, 2H), 1.71 (h, *J* = 7.3 Hz, 2H), 1.08 (t, *J* = 7.4 Hz, 3H).

^{13}C NMR (101 MHz, $CDCl_3$) δ 176.6, 166.3 (d, *J* = 256.0 Hz), 133.4 (d, *J* = 2.9 Hz), 132.2 (d, *J* = 9.6 Hz), 115.7 (d, *J* = 22.2 Hz), 96.9, 79.5, 21.3, 21.1, 13.6.

^{19}F NMR (376 MHz, $CDCl_3$) δ -103.7 – -103.8 (m).

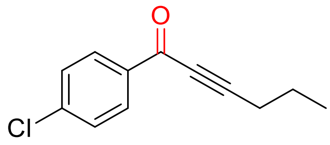

1-(4-chlorophenyl) hex-2-yn-1-one[5]

Yellow oil (22.7 mg, 55% yield), purified by column chromatography (SiO_2, Pentane/EA= 50:1).

^{1}H NMR (400 MHz, $CDCl_3$) δ 8.04 – 7.96 (m, 2H), 7.41 – 7.33 (m, 2H), 2.41 (t, *J* = 7.0 Hz, 2H), 1.71 – 1.57 (m, 2H), 1.01 (t, *J* = 7.4 Hz, 3H).

^{13}C NMR (101 MHz, $CDCl_3$) δ 176.9, 140.4, 135.3, 130.8, 128.8, 97.2, 79.5, 21.3, 21.1, 13.6.

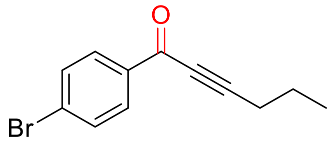

1-(4-bromophenyl) hex-2-yn-1-one[4]

Yellow oil (18.9 mg, 38% yield), purified by column chromatography (SiO_2, Pentane/EA= 50:1).

^{1}H NMR (400 MHz, $CDCl_3$) δ 8.01 – 7.97 (m, 2H), 7.63 – 7.59 (m, 2H), 2.48 (t, *J* = 7.1 Hz, 2H), 1.71 (h, *J* = 7.3 Hz, 2H), 1.08 (t, *J* = 7.4 Hz, 3H).

^{13}C NMR (101 MHz, $CDCl_3$) δ 177.1, 135.7, 131.8, 130.9, 129.3, 97.3, 79.5, 21.3, 21.1, 13.6.

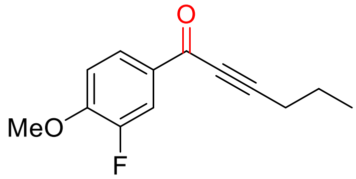

1-(3-fluoro-4-methoxyphenyl) hex-2-yn-1-one

Yellow oil (32.1 mg, 73% yield), purified by column chromatography (SiO_2, Pentane/EA= 20:1).

^{1}H NMR (400 MHz, $CDCl_3$) δ 7.96 – 7.88 (m, 1H), 7.82 (dd, *J* = 11.6, 2.1 Hz, 1H), 7.00 (t, *J* = 8.3 Hz, 1H), 3.95 (s, 3H), 2.46 (t, *J* = 7.0 Hz, 2H), 1.76 – 1.62 (m, 2H), 1.07 (t, *J* = 7.4 Hz, 3H).

^{13}C NMR (101 MHz, $CDCl_3$) δ 176.0 (d, J = 2.3 Hz), 152.6 (d, J = 11.0 Hz), 151.8 (d, J = 248.2 Hz), 130.4 (d, J = 5.4 Hz), 127.1 (d, J = 3.2 Hz), 116.6 (d, J = 19.4 Hz), 112.2 (d, J = 1.9 Hz), 96.4, 79.3, 56.3, 21.3, 21.1, 13.6.

^{19}F NMR (376 MHz, $CDCl_3$) δ -134.2 (dd, J = 11.6, 7.9 Hz).

HRMS (ESI-TOF): m/z calcd. for $C_{13}H_{14}FO_2$ [M+H $^+$] 221.0972, found 221.0972.

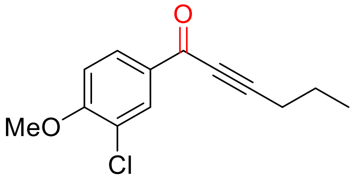

1-(3-chloro-4-methoxyphenyl) hex-2-yn-1-one

Yellow oil (30.7 mg, 65% yield), purified by column chromatography (SiO_2, Pentane/EA= 20:1).

^{1}H NMR (400 MHz, $CDCl_3$) δ 8.13 (d, J = 2.1 Hz, 1H), 8.03 (dd, J = 8.6, 2.1 Hz, 1H), 6.97 (d, J = 8.7 Hz, 1H), 3.97 (s, 3H), 2.47 (t, J = 7.0 Hz, 2H), 1.76 – 1.63 (m, 2H), 1.07 (t, J = 7.4 Hz, 3H).

^{13}C NMR (101 MHz, $CDCl_3$) δ 175.8, 159.4, 131.6, 130.7, 130.0, 122.8, 111.2, 96.7, 79.3, 56.4, 21.3, 21.1, 13.6.

HRMS (ESI-TOF): m/z calcd. for $C_{13}H_{14}ClO_2$ [M+H $^+$] 237.0677, found 237.0683.

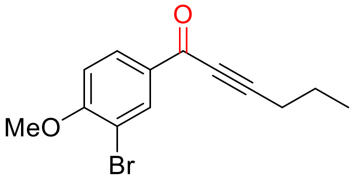

1-(3-bromo-4-methoxyphenyl) hex-2-yn-1-one

Yellow oil (38.1 mg, 64% yield), purified by column chromatography (SiO_2, Pentane/EA= 20:1).

^{1}H NMR (400 MHz, $CDCl_3$) δ 8.31 (d, J = 2.2 Hz, 1H), 8.07 (dd, J = 8.6, 2.1 Hz, 1H), 6.94 (d, J = 8.7 Hz, 1H), 3.97 (s, 3H), 2.47 (t, J = 7.0 Hz, 2H), 1.70 (h, J = 7.3 Hz, 2H), 1.08 (t, J = 7.4 Hz, 3H).

^{13}C NMR (101 MHz, $CDCl_3$) δ 175.7, 160.2, 134.8, 131.2, 130.7, 111.8, 111.0, 96.7, 79.3, 56.5, 21.3, 21.1, 13.6.

HRMS (ESI-TOF): m/z calcd. for $C_{13}H_{14}BrO_2$ [M+H $^+$] 281.0172, found 281.0176.

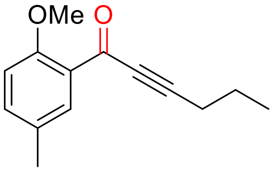

1-(2-methoxy-5-methylphenyl) hex-2-yn-1-one

Yellow oil (30.7 mg, 71% yield), purified by column chromatography (SiO_2, Pentane/EA= 20:1).

^{1}H NMR (300 MHz, $CDCl_3$) δ 7.82 – 7.74 (m, 1H), 7.31 – 7.26 (m, 1H), 6.86 (d, J = 8.5 Hz, 1H), 3.87 (s, 3H), 2.43 (t, J = 7.0 Hz, 2H), 2.31 (s, 3H), 1.75 – 1.57 (m, 2H), 1.05 (t, J = 7.4 Hz, 3H).

^{13}C NMR (75 MHz, $CDCl_3$) δ 177.2, 157.6, 135.2, 132.9, 129.4, 126.5, 112.2, 95.0, 82.0, 55.9, 21.3, 21.2, 20.2, 13.5.

HRMS (ESI-TOF): m/z calcd. for $C_{14}H_{17}O_2$ [M+H $^+$] 217.1223, found 217.1226.

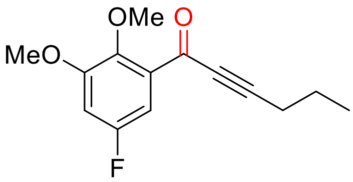

1-(5-fluoro-2,3-dimethoxyphenyl) hex-2-yn-1-one

Yellow oil (33.9 mg, 60% yield), purified by column chromatography (SiO_2, Pentane/EA= 20:1).

^{1}H NMR (300 MHz, $CDCl_3$) δ 7.38 (d, *J* = 6.9 Hz, 1H), 6.55 (d, *J* = 12.1 Hz, 1H), 3.86 (s, 3H), 3.82 (s, 3H), 2.38 (td, *J* = 7.0, 0.8 Hz, 2H), 1.60 (h, *J* = 7.2 Hz, 2H), 0.99 (t, *J* = 7.4 Hz, 3H).

^{13}C NMR (75 MHz, $CDCl_3$) δ 173.0 (d, *J* = 1.3 Hz), 158.3 (d, *J* = 257.0 Hz), 154.9 (d, *J* = 10.3 Hz), 145.1 (d, *J* = 2.2 Hz), 117.2 (d, *J* = 8.5 Hz), 111.6 (d, *J* = 2.0 Hz), 100.2 (d, *J* = 28.3 Hz), 96.0 (d, *J* = 4.5 Hz), 81.6, 56.5, 56.3, 21.3, 21.2, 13.5.

^{19}F NMR (282 MHz, $CDCl_3$) δ -115.7 (dd, *J* = 12.1, 6.9 Hz).

HRMS (ESI-TOF): m/z calcd. for $C_{14}H_{16}FO_3$ [M+H $^+$] 251.1078, found 251.1080.

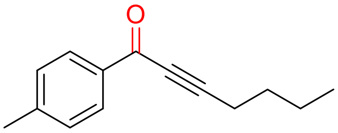

1-(*p*-tolyl) hept-2-yn-1-one

Yellow oil (33.7 mg, 84% yield), purified by column chromatography (SiO_2, Pentane/EA= 50:1).

^{1}H NMR (300 MHz, $CDCl_3$) δ 8.07 – 7.97 (m, 2H), 7.26 (dt, *J* = 8.0, 0.7 Hz, 2H), 2.49 (t, *J* = 7.0 Hz, 2H), 2.42 (s, 3H), 1.73 – 1.57 (m, 2H), 1.56 – 1.44 (m, 2H), 0.96 (t, *J* = 7.3 Hz, 3H).

^{13}C NMR (75 MHz, $CDCl_3$) δ 177.9, 144.8, 134.6, 129.6, 129.2, 96.2, 79.7, 29.8, 22.0, 21.7, 18.8, 13.5.

HRMS (ESI-TOF): m/z calcd. for $C_{14}H_{17}O$ [M+H $^+$] 201.1274, found 201.1275.

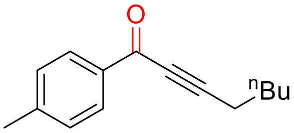

1-(*p*-tolyl) oct-2-yn-1-one[6]

Yellow oil (34.4 mg, 80% yield), purified by column chromatography (SiO_2, Pentane/EA= 50:1).

^{1}H NMR (400 MHz, $CDCl_3$) δ 8.07 – 7.98 (m, 2H), 7.30 – 7.20 (m, 2H), 2.48 (t, *J* = 7.1 Hz, 2H), 2.42 (s, 3H), 1.73 – 1.61 (m, 2H), 1.51 – 1.41 (m, 2H), 1.40 – 1.32 (m, 2H), 0.92 (t, *J* = 7.2 Hz, 3H).

^{13}C NMR (101 MHz, $CDCl_3$) δ 177.9, 144.8, 134.6, 129.6, 129.1, 96.3, 79.7, 31.1, 27.5, 22.1, 21.7, 19.1, 13.9.

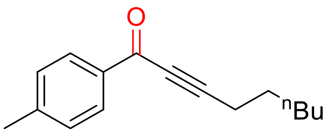

1-(*p*-tolyl) non-2-yn-1-one[7]

Yellow oil (30.0 mg, 64% yield), purified by column chromatography (SiO_2, Pentane/EA= 50:1).

^{1}H NMR (300 MHz, $CDCl_3$) δ 8.05 – 8.00 (m, 2H), 7.29 – 7.22 (m, 2H), 2.48 (t, *J* = 7.1 Hz, 2H), 2.42 (s, 3H), 1.74 – 1.59 (m, 2H), 1.55 – 1.42 (m, 2H), 1.38 – 1.24 (m, 4H), 0.96 – 0.85 (m, 3H).

^{13}C NMR (75 MHz, $CDCl_3$) δ 177.9, 144.8, 134.6, 129.6, 129.1, 96.3, 79.7, 31.2, 28.6, 27.8, 22.5, 21.7, 19.2, 14.0.

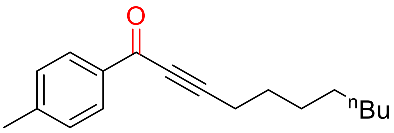

1-(*p*-tolyl) undec-2-yn-1-one

Yellow oil (37.6 mg, 74% yield), purified by column chromatography (SiO_2, Pentane/EA= 50:1).

^{1}H NMR (400 MHz, $CDCl_3$) δ 8.07 – 7.99 (m, 2H), 7.31 – 7.22 (m, 2H), 2.48 (t, *J* = 7.1 Hz, 2H), 2.42 (s, 3H), 1.72 – 1.61 (m, 2H), 1.52 – 1.39 (m, 2H), 1.34 – 1.24 (m, 8H), 0.95 – 0.82 (m, 3H).

^{13}C NMR (101 MHz, $CDCl_3$) δ 177.9, 144.8, 134.6, 129.6, 129.1, 96.3, 79.7, 31.8, 29.1, 29.0, 28.9, 27.8, 22.6, 21.7, 19.2, 14.0.

HRMS (ESI-TOF): m/z calcd. for $C_{18}H_{25}O$ [M+H $^{+}$] 257.1900, found 257.1900.

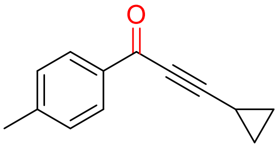

3-cyclopropyl-1-(*p*-tolyl) prop-2-yn-1-one[8]

Yellow oil (30.3 mg, 82% yield), purified by column chromatography (SiO_2, Pentane/EA= 50:1).

^{1}H NMR (400 MHz, $CDCl_3$) δ 7.96 – 7.87 (m, 2H), 7.22 – 7.12 (m, 2H), 2.34 (s, 3H), 1.51 – 1.40 (m, 1H), 1.02 – 0.89 (m, 4H).

^{13}C NMR (101 MHz, $CDCl_3$) δ 177.6, 144.7, 134.6, 129.5, 129.1, 100.4, 75.5, 21.7, 9.8, -0.1.

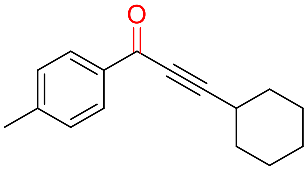

3-cyclohexyl-1-(*p*-tolyl) prop-2-yn-1-one[9]

Yellow oil (31.8 mg, 70% yield), purified by column chromatography (SiO_2, Pentane/EA= 50:1).

^{1}H NMR (400 MHz, $CDCl_3$) δ 8.05 – 8.00 (m, 2H), 7.29 – 7.23 (m, 2H), 2.74 – 2.60 (m, 1H), 2.42 (s, 3H), 1.98 – 1.87 (m, 2H), 1.82 – 1.71 (m, 2H), 1.67 – 1.33 (m, 6H).

^{13}C NMR (101 MHz, $CDCl_3$) δ 178.1, 144.8, 134.7, 129.6, 129.1, 99.8, 79.6, 31.7, 29.3, 25.6, 24.7, 21.7.

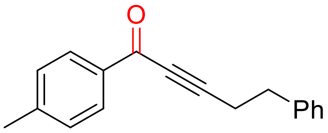

5-phenyl-1-(*p*-tolyl) pent-2-yn-1-one[10]

Yellow oil (34.7 mg, 70% yield), purified by column chromatography (SiO_2, Pentane/EA= 50:1).

^{1}H NMR (400 MHz, $CDCl_3$) δ 7.85 – 7.76 (m, 2H), 7.29 – 7.23 (m, 2H), 7.21 – 7.16 (m, 3H), 7.15 – 7.11 (m, 2H), 2.90 (t, *J* = 7.3 Hz, 2H), 2.77 – 2.68 (m, 2H), 2.33 (s, 3H).

^{13}C NMR (101 MHz, $CDCl_3$) δ 177.8, 144.9, 139.7, 134.4, 129.7, 129.1, 128.6, 128.5, 126.6, 94.8, 80.2, 33.9, 21.7, 21.3.

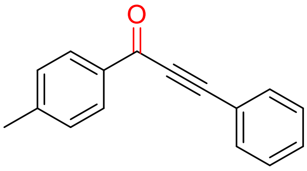

3-phenyl-1-(*p*-tolyl) prop-2-yn-1-one[7]

Yellow oil (39.0 mg, 88% yield; 95% purity), purified by column chromatography (SiO_2, Pentane/EA= 50:1).

^{1}H NMR (400 MHz, $CDCl_3$) δ 8.16 – 8.07 (m, 2H), 7.73 – 7.63 (m, 2H), 7.50 – 7.38 (m, 3H), 7.35 – 7.27 (m, 2H), 2.44 (s, 3H).

^{13}C NMR (101 MHz, $CDCl_3$) δ 177.6, 145.2, 134.5, 133.0, 130.6, 129.6, 129.3, 128.6, 120.2, 92.5, 86.9, 21.8.

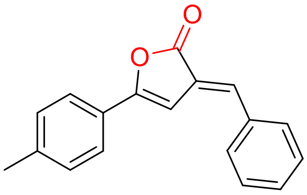

(*E*)-3-benzylidene-5-(*p*-tolyl) furan-2(3*H*)-one[11]

Yellow Solid (31.3 mg, 59% yield), purified by column chromatography (SiO_2, Pentane/EA= 50:1).

^{1}H NMR (400 MHz, $CDCl_3$) δ 7.61 – 7.51 (m, 4H), 7.43 – 7.29 (m, 4H), 7.21 – 7.13 (m, 2H), 6.80 (d, *J* = 1.0 Hz, 1H), 2.32 (s, 3H).

^{13}C NMR (101 MHz, $CDCl_3$) δ 169.5, 157.2, 141.0, 135.2, 134.7, 130.6, 130.1, 130.0, 129.6, 129.0, 125.5, 125.3, 99.0, 21.6.

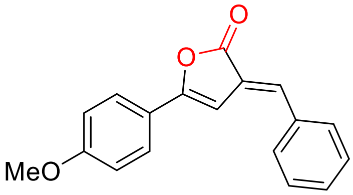

(*E*)-3-benzylidene-5-(4-methoxyphenyl) furan-2(3*H*)-one[11]

Yellow Solid (33.8 mg, 61% yield; 95% purity), purified by column chromatography (SiO_2, Pentane/EA= 20:1).

^{1}H NMR (400 MHz, $CDCl_3$) δ 7.75 – 7.68 (m, 2H), 7.66 – 7.58 (m, 2H), 7.50 – 7.36 (m, 4H), 7.01 – 6.91 (m, 2H), 6.80 (d, *J* = 1.0 Hz, 1H), 3.86 (s, 3H).

^{13}C NMR (101 MHz, $CDCl_3$) δ 169.6, 161.5, 156.9, 135.4, 133.9, 129.9, 129.4, 129.0, 127.0, 125.6, 120.7, 114.4, 98.0, 55.4.

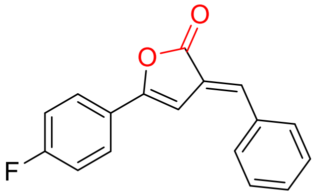

(*E*)-3-benzylidene-5-(4-fluorophenyl) furan-2(3*H*)-one[11]

Yellow Solid (26.6 mg, 50% yield; 95% purity), purified by column chromatography (SiO_2, Pentane/EA= 50:1).

^{1}H NMR (400 MHz, $CDCl_3$) δ 7.72 – 7.62 (m, 2H), 7.59 – 7.50 (m, 2H), 7.44 – 7.29 (m, 4H), 7.11 – 6.99 (m, 2H), 6.79 (s, 1H).

^{13}C NMR (101 MHz, $CDCl_3$) δ 169.2, 163.9 (d, *J* = 252.3 Hz), 155.9 (d, *J* = 1.7 Hz), 135.5 (d, *J* = 1.7 Hz), 135.1, 130.3, 130.0, 129.1, 127.4 (d, *J* = 8.5 Hz), 125.3, 124.4 (d, *J* = 3.6 Hz), 116.2 (d, *J* = 22.3 Hz), 99.5 (d, *J* = 2.3 Hz).

^{19}F NMR (376 MHz, $CDCl_3$) δ -107.4 – -109.8 (m).

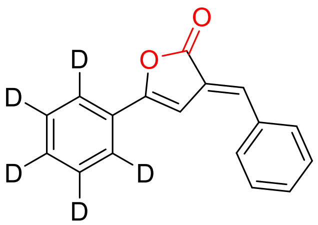

(*E*)-3-benzylidene-5-(phenyl-*d*$_5$) furan-2(3*H*)-one

Yellow Solid (29.5 mg, 58% yield; 95% purity), purified by column chromatography (SiO_2, Pentane/EA= 50:1).

^{1}H NMR (400 MHz, $CDCl_3$) δ 7.59 – 7.49 (m, 2H), 7.44 – 7.29 (m, 4H), 6.85 (d, *J* = 1.1 Hz, 1H).

^{13}C NMR (101 MHz, $CDCl_3$) δ 169.3, 156.9, 135.4, 135.1, 130.2, 130.1, 129.1, 128.6 – 128.0 (m), 127.8, 125.4, 125.3 – 124.5 (m), 99.8.

HRMS (ESI-TOF): m/z calcd. for $C_{17}H_8D_5O_2$ [M+H $^+$] 254.1224, found 254.1231.

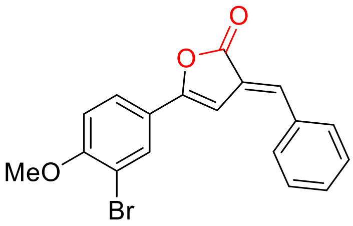

(*E*)-3-benzylidene-5-(3-bromo-4-methoxyphenyl) furan-2(3*H*)-one

Yellow Solid (47.9 mg, 67% yield), purified by column chromatography (SiO_2, Pentane/EA= 20:1).

^{1}H NMR (400 MHz, $CDCl_3$) δ 7.94 (d, *J* = 2.2 Hz, 1H), 7.72 – 7.58 (m, 3H), 7.51 – 7.37 (m, 4H), 6.95 (d, *J* = 8.7 Hz, 1H), 6.82 (d, *J* = 1.0 Hz, 1H), 3.95 (s, 3H).

^{13}C NMR (101 MHz, $CDCl_3$) δ 169.2, 157.5, 155.4, 135.1, 135.1, 130.3, 130.2, 130.0, 129.1, 125.9, 125.2, 122.0, 112.4, 111.8, 99.0, 56.4.

HRMS (ESI-TOF): m/z calcd. for $C_{18}H_{13}BrO_3Na$ [M+Na $^+$] 378.9940, found 378.9935.

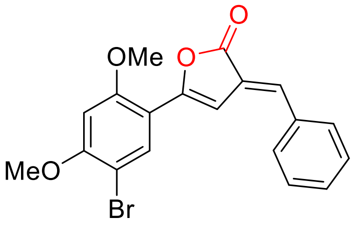

(*E*)-3-benzylidene-5-(5-bromo-2,4-dimethoxyphenyl) furan-2(3*H*)-one

Yellow Solid (43.3 mg, 56% yield; 95% purity), purified by column chromatography (SiO_2, Pentane/EA= 5:1).

^{1}H NMR (300 MHz, $CDCl_3$) δ 7.96 (s, 1H), 7.65 – 7.55 (m, 2H), 7.50 – 7.37 (m, 3H), 7.33 (s, 1H), 7.08 (d, *J* = 1.1 Hz, 1H), 6.50 (s, 1H), 4.01 (s, 3H), 3.95 (s, 3H).

^{13}C NMR (75 MHz, $CDCl_3$) δ 169.0, 158.7, 157.9, 152.2, 135.4, 134.3, 131.6, 130.1, 129.9, 129.0, 125.8, 110.9, 103.7, 102.6, 96.0, 56.4, 56.0.

HRMS (ESI-TOF): m/z calcd. for $C_{19}H_{16}BrO_4$ [M+H $^+$] 387.0227, found 387.0225.

5. Control experiments

5.1 Radical inhibition experiments

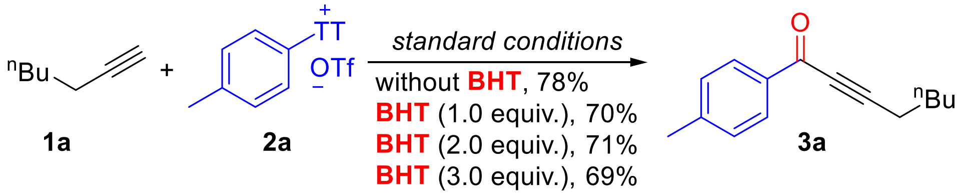

Scheme S1. Radical inhibition experiments

A 4 mL snap vial equipped with a magnetic stir bar was charged with PdI_2 (0.004 mmol, 2 mol %), PPh_3 (0.012 mmol, 6 mol%), aryl sulfonium salts (0.3 mmol, 1.5 equiv.) and closed with a rubber-based septum. Different amounts of 2,6-di-tert-butyl-4-methylphenol (BHT) (1 – 3 equiv.) were added sequentially to the corresponding vials. The vial was evacuated and backfilled with argon. Degassed DMF (2.0 mL), DIPEA (0.4 mmol, 2.0 equiv.), and hept-1-yne (0.2 mmol, 1.0 equiv.) were added via syringe. The vial was then connected to atmosphere with a cannula and transferred into a 300 mL Parr 4560 series autoclave, under argon counterflow. The closed autoclave was flushed three times with nitrogen (~ 5 bar), three times with CO (~ 5 bar), and 10 bar of carbon monoxide (measured by pressure meter) was charged. The autoclave was then placed into an aluminum block on a magnetic stirrer. The reaction mixture was stirred (500 rpm) at 80 °C 15 h. And a proper amount of solvent was taken for GC analysis. The result is shown above.

As BHT was added to the reaction system, the reaction was gradually inhibited.

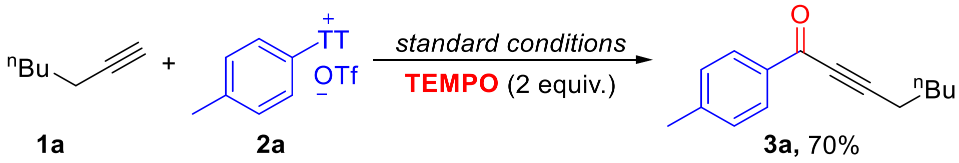

Scheme S4. Radical capture experiment

A 4 mL snap vial equipped with a magnetic stir bar was charged PdI_2 (0.004 mmol, 2 mol %), PPh_3 (0.012 mmol, 6 mol%), aryl sulfonium salts (0.3 mmol, 1.5 equiv.), 2,2,6,6-tetramethylpiperidine-1-oxyl (TEMPO) (2 equiv.) and closed with a rubber-based septum. The vial was evacuated and backfilled with argon. Degassed DMF (2.0 mL), DIPEA (0.4 mmol, 2.0 equiv.), and hept-1-yne (0.2 mmol, 1.0 equiv.) were added via syringe. The vial was then connected to atmosphere with a cannula and transferred into a 300 mL Parr 4560 series autoclave, under argon counterflow. The closed autoclave was flushed three times with nitrogen (~ 5 bar), three times with CO (~ 5 bar), and 10 bar of carbon monoxide (measured by pressure meter) was charged. The autoclave was then placed into an aluminum block on a magnetic stirrer. The reaction mixture was stirred (500 rpm) at 80 °C 15 h. And a proper amount of solvent was taken for GC analysis. The result is shown above.

5.2 Radical capture experiment

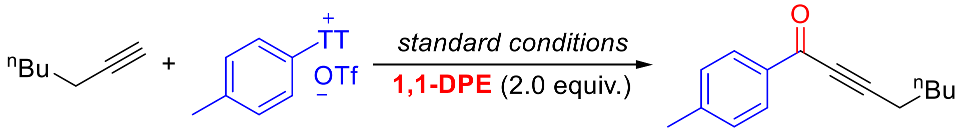

A 4 mL snap vial equipped with a magnetic stir bar was charged PdI_2 (0.004 mmol, 2 mol %), PPh_3 (0.012 mmol, 6 mol%), aryl sulfonium salts (0.3 mmol, 1.5 equiv.), and closed with a rubber-based septum. The vial was evacuated and backfilled with argon. Degassed DMF (2.0 mL), DIPEA (0.4 mmol, 2.0 equiv.), 1,1-DPE (2 equiv.) and hept-1-yne (0.2 mmol, 1.0 equiv.) were added via syringe. The vial was then connected to atmosphere with a cannula and transferred into a 300 mL Parr 4560 series autoclave, under argon counterflow. The closed autoclave was flushed three times with nitrogen (~ 5 bar), three times with CO (~ 5 bar), and 10 bar of carbon monoxide (measured by pressure meter) was charged. The autoclave was then placed into an aluminum block on a magnetic stirrer. The reaction mixture was stirred (500 rpm) at 80 °C 15 h. And a proper amount of solvent was taken for GC analysis. The result is shown above.

6. Reference

(1) (a) Gu, X.-W.; Zhao, Y.-H.; Wu, X.-F. [3 + 2] Cycloaddition of azides with arynes formed via C–H deprotonation of aryl sulfonium salts. *Green Chem.* **2023**, *25* (16), 6282-6286. (b) Tian, Z.-Y.; Lin, Z.-H.; Zhang, C.-P. Pd/Cu-Catalyzed C–H/C–H Cross Coupling of (Hetero)Arenes with Azoles through Arylsulfonium Intermediates. *Org. Lett.* **2021**, *23* (11), 4400-4405.

(2) (a) Li, J.; Chen, J.; Sang, R.; Ham, W.-S.; Plutschack, M. B.; Berger, F.; Chabbra, S.; Schnegg, A.; Genicot, C.; Ritter, T. Photoredox catalysis with aryl sulfonium salts enables site-selective late-stage fluorination. *Nat. Chem.* **2020**, *12* (1), 56-62. (b) Tang, Y.; Cai, Y.; Xie, Z.; Gao, Z.; Chen, X.; Yi, J. Multicomponent reactions to access S-aryl dithiocarbamates via an electron donor–acceptor complex under open-to-air conditions. *Org. Biomol. Chem.* **2024**, *22* (7), 1378-1385.

(3) Bakherad, M.; Keivanloo, A.; Bahramian, B.; Jajarmi, S. Synthesis of Ynones via Recyclable Polystyrene-Supported Palladium(0) Complex Catalyzed Acylation of Terminal Alkynes with Acyl Chlorides under Copper- and Solvent-Free Conditions. *Synlett* **2011**, *2011* (03), 311-314.

(4) Chu, X.-P.; Zhou, Q.-F.; Zhao, S.; Ge, F.-F.; Fu, M.; Chen, J.-P.; Lu, T. Synthesis and biological evaluation of 3-amino-2-pyrones as selective cyclooxygenase-1 (COX-1) inhibitors. *Chin. Chem. Lett.* **2013**, *24* (2), 120-122.

(5) Kandasamy, M.; Ganesan, B.; Hung, M.-Y.; Lin, W.-Y. Fast and Efficient Continuous Flow Method for the Synthesis of Ynones and Pyrazoles. *Eur. J. Org. Chem.* **2019**, *2019* (20), 3183-3189.

(6) Xia, X.-F.; Zhao, M.; He, W.; Zou, L.; San, X.; Wang, D. Metal-Free Oxidative [5+1] Cyclization of 1,5-Enynes for the Synthesis of Pyrazine 1-Oxide. *Adv. Synth. Catal.* **2020**, *362* (17), 3621-3626.

(7) Chen, L.; Li, C.-J. A Remarkably Efficient Coupling of Acid Chlorides with Alkynes in Water. *Org. Lett.* **2004**, *6* (18), 3151-3153.

(8) Liu, K.; Cheng, S.-J.; Luo, G.; Ye, Z.-S. Synthesis of 1-Substituted Cyclopropylamines via Formal Tertiary Csp3–H Amination of Cyclopropanes. *Org. Lett.* **2021**, *23* (23), 9309-9314.

(9) Sarkar, R.; Gupta, A.; Jamatia, R.; Pal, A. K. Reduced graphene oxide supported copper oxide nanocomposites: An efficient heterogeneous and reusable catalyst for the synthesis of ynones, 1,3-diynes and 1,5-benzodiazepines in one-pot under sustainable reaction conditions. *Appl. Organomet. Chem.* **2020**, *34* (7), e5646.

(10) Blay, G.; Cardona, L.; Fernández, I.; Pedro, J. R. Cobalt(III) Complex Catalyzed Aerobic Oxidation of Propargylic Alcohols. *Synthesis* **2007**, *2007* (21), 3329-3332.

(11) Wu, X.-F.; Jiao, H.; Neumann, H.; Beller, M. Palladium-Catalyzed Carbonylative Coupling of Aryl Iodides and Benzyl Acetylenes to 3-Alkylidenefuran-2-ones under Mild Conditions and Its Density Functional Theory Modeling. *Chem. Eur. J.* **2012**, *18* (50), 16177-16185.

7. NMR spectra of products

240809.410.10.fid — Yanhua Zhao ZYH-291-2 — Au1H CDCl3 {C:\Bruker\TopSpin3.6.2} 2408 10 - 300 MHz

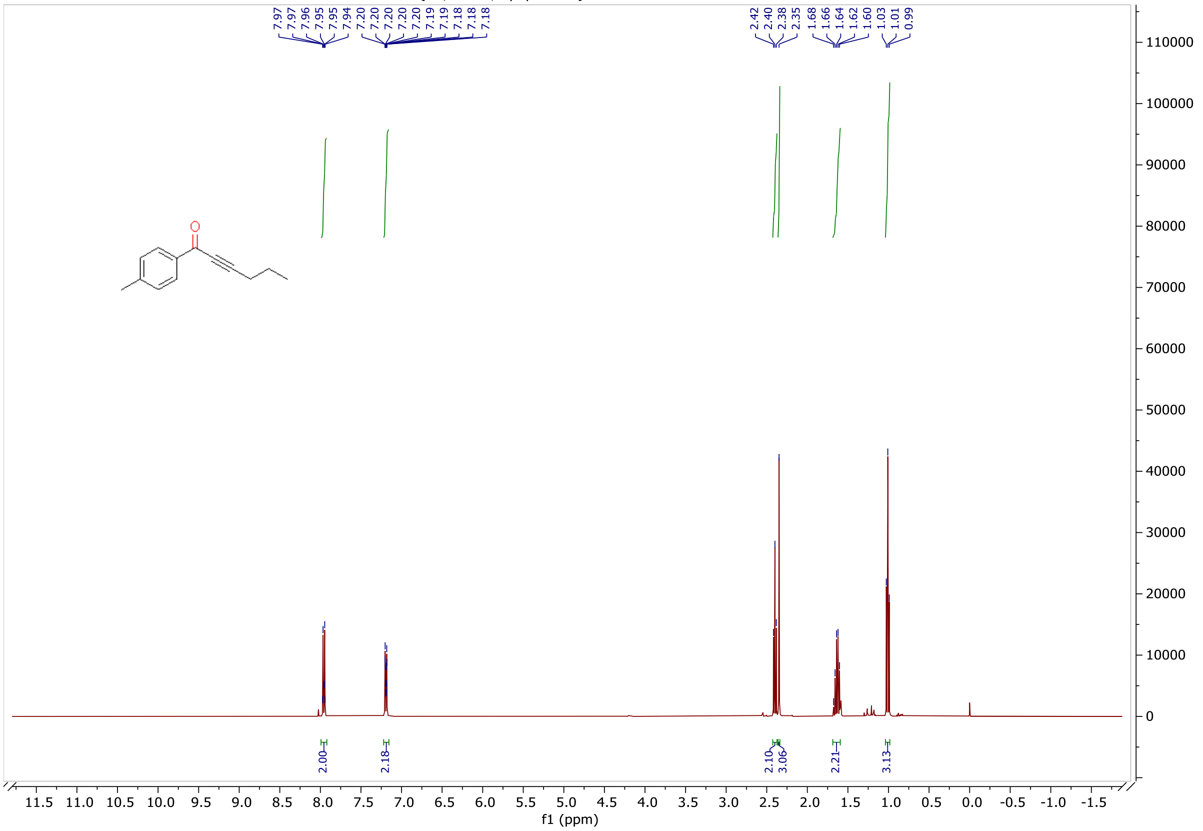

240809.410.11.fid — Yanhua Zhao ZYH-291-2 — Au13C CDCl3 {C:\Bruker\TopSpin3.6.2} 2408 10 - 75 MHz

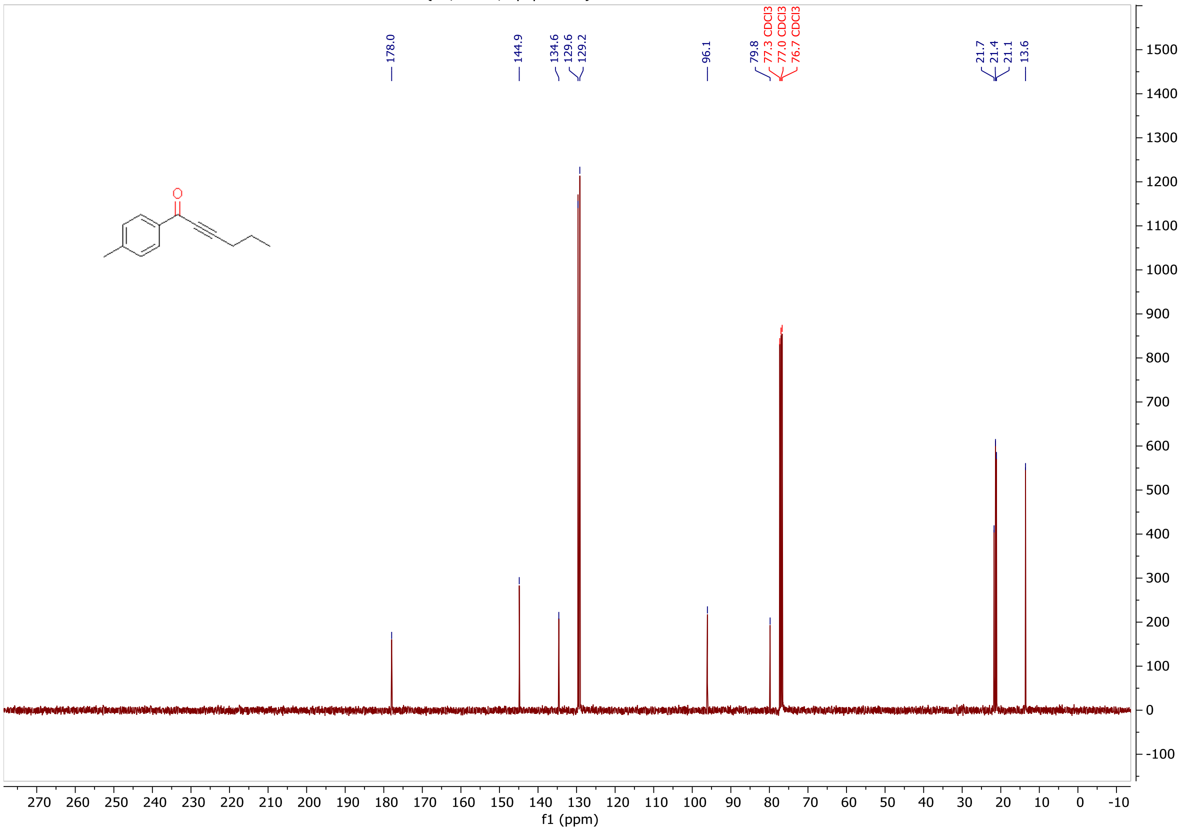

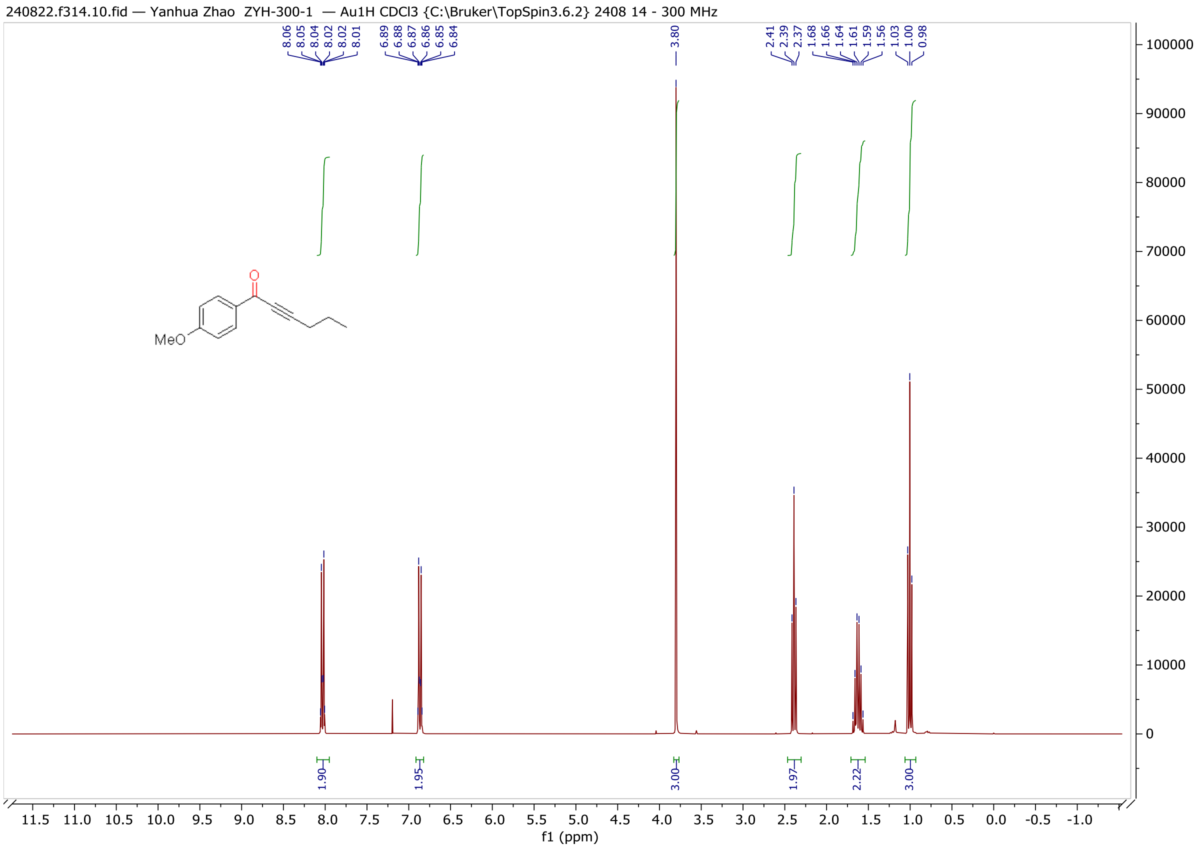
240822.f314.10.fid — Yanhua Zhao ZYH-300-1 — Au1H CDCl3 {C:\Bruker\TopSpin3.6.2} 2408 14 - 300 MHz
8.06 8.05 8.04 8.02 8.02 8.01
6.89 6.88 6.87 6.86 6.85 6.84
3.80
2.41 2.39 2.37 1.68 1.66 1.64 1.61 1.59 1.56 1.03 1.00 0.98
MeO
O
1.90
1.95
3.00
1.97
2.22
3.00
f1 (ppm)

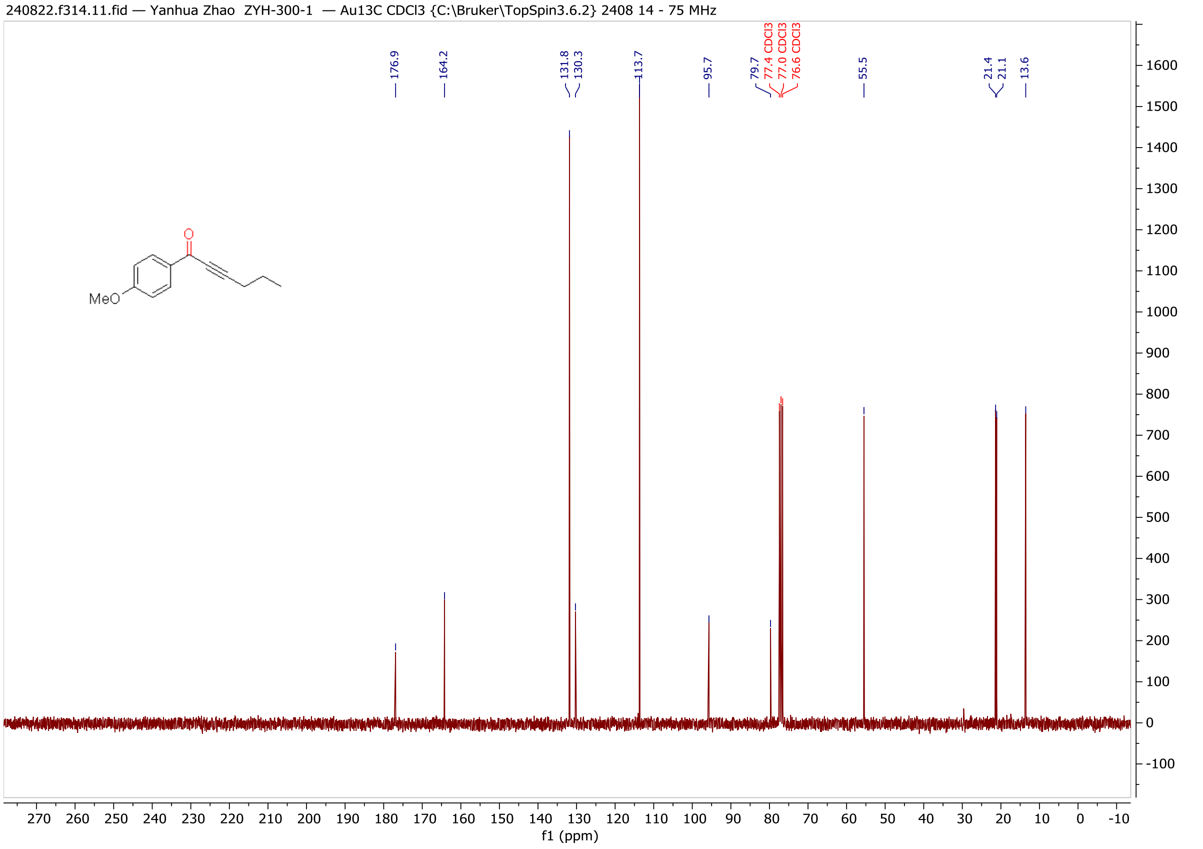
240822.f314.11.fid — Yanhua Zhao ZYH-300-1 — Au13C CDCl3 {C:\Bruker\TopSpin3.6.2} 2408 14 - 75 MHz
176.9
164.2
131.8 130.3
113.7
95.7
79.7 77.4 CDCl3 77.0 CDCl3 76.6 CDCl3
55.5
21.4 21.1
13.6
MeO
O
f1 (ppm)

240826.431.10.fid — Yanhua Zhao ZYH-300-6 — Au1H CDCl3 {C:\Bruker\TopSpin3.6.2} 2408 31 - 300 MHz

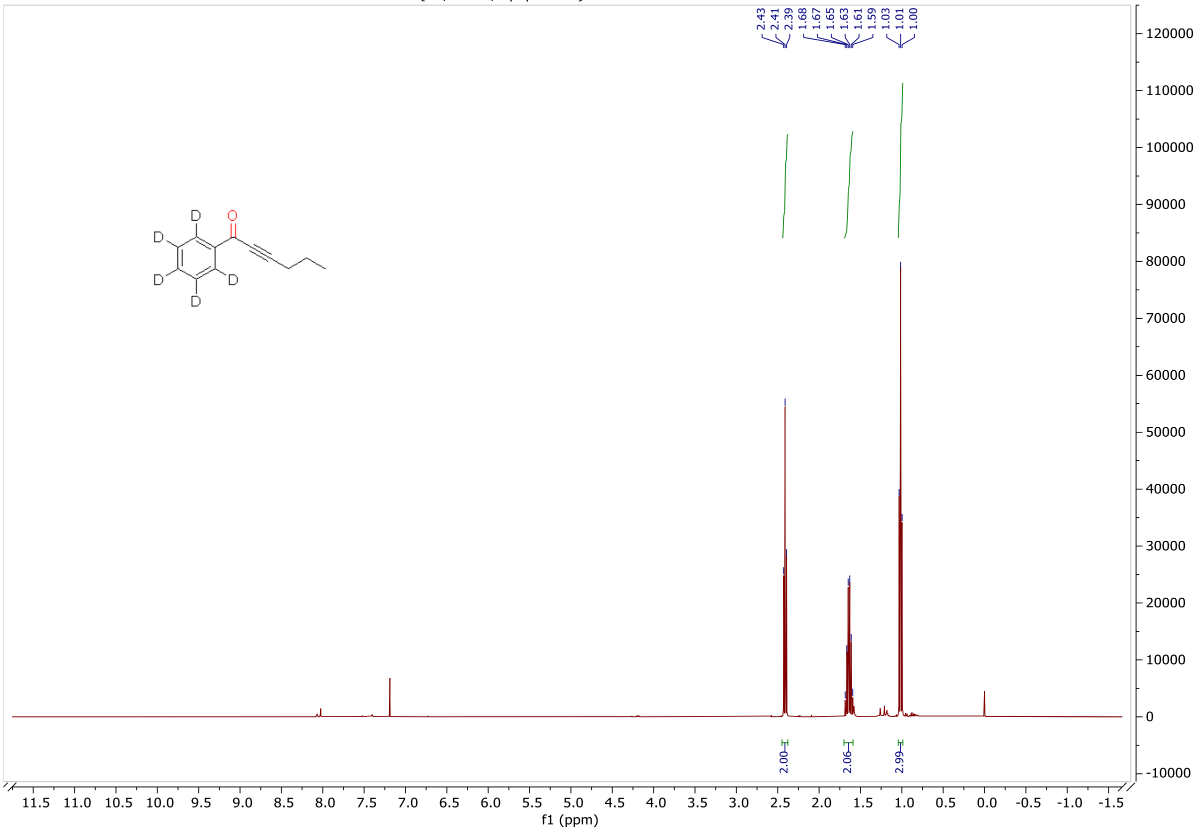

240826.431.11.fid — Yanhua Zhao ZYH-300-6 — Au13C CDCl3 {C:\Bruker\TopSpin3.6.2} 2408 31 - 75 MHz

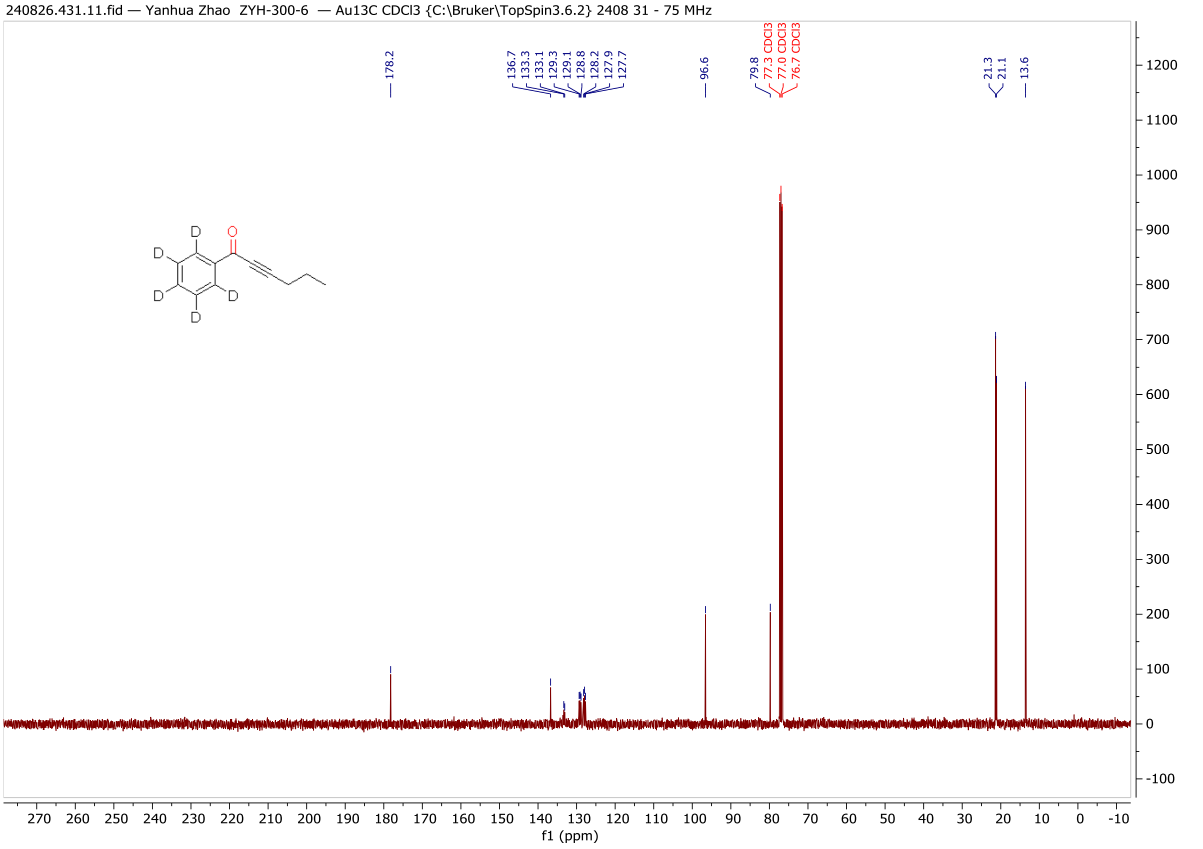

240826.428.10.fid — Yanhua Zhao ZYH-300-3 — Au1H CDCl3 {C:\Bruker\TopSpin3.6.2} 2408 28 - 300 MHz

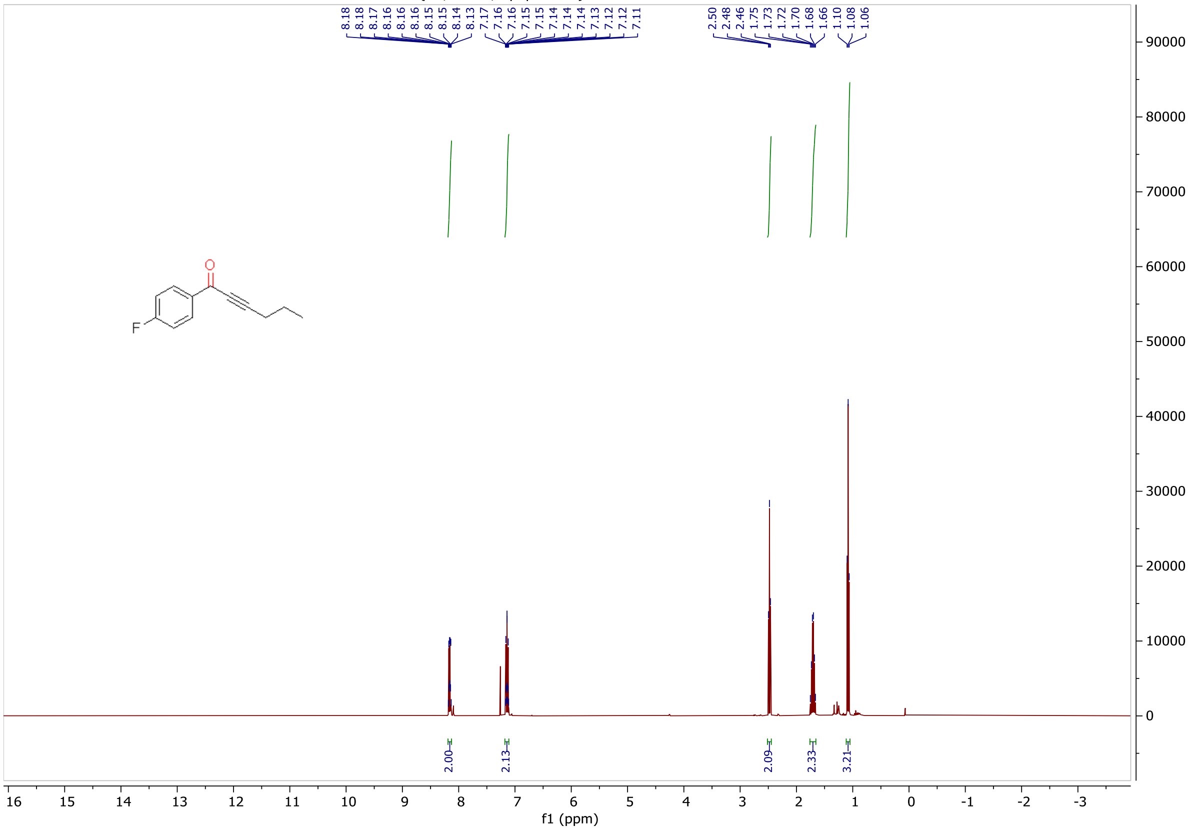

240826.428.11.fid — Yanhua Zhao ZYH-300-3 — Au13C CDCl3 {C:\Bruker\TopSpin3.6.2} 2408 28 - 75 MHz

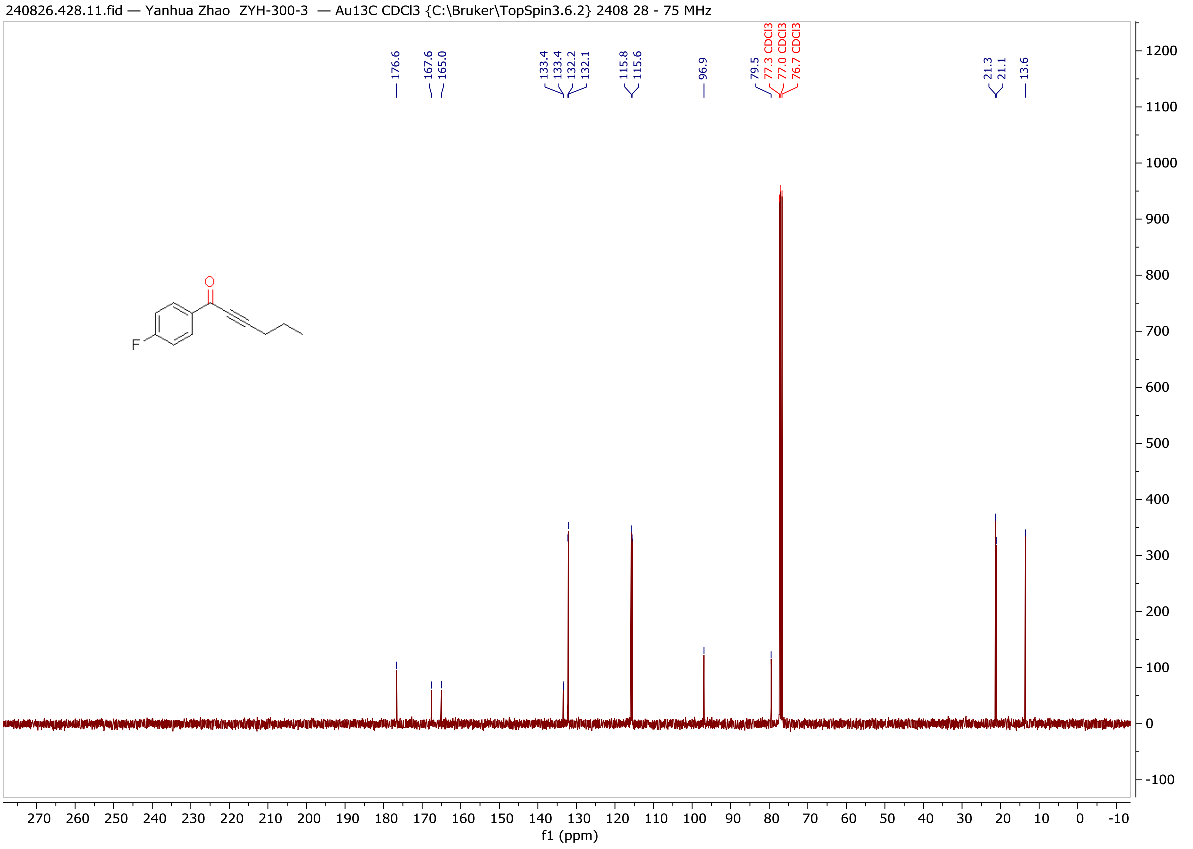

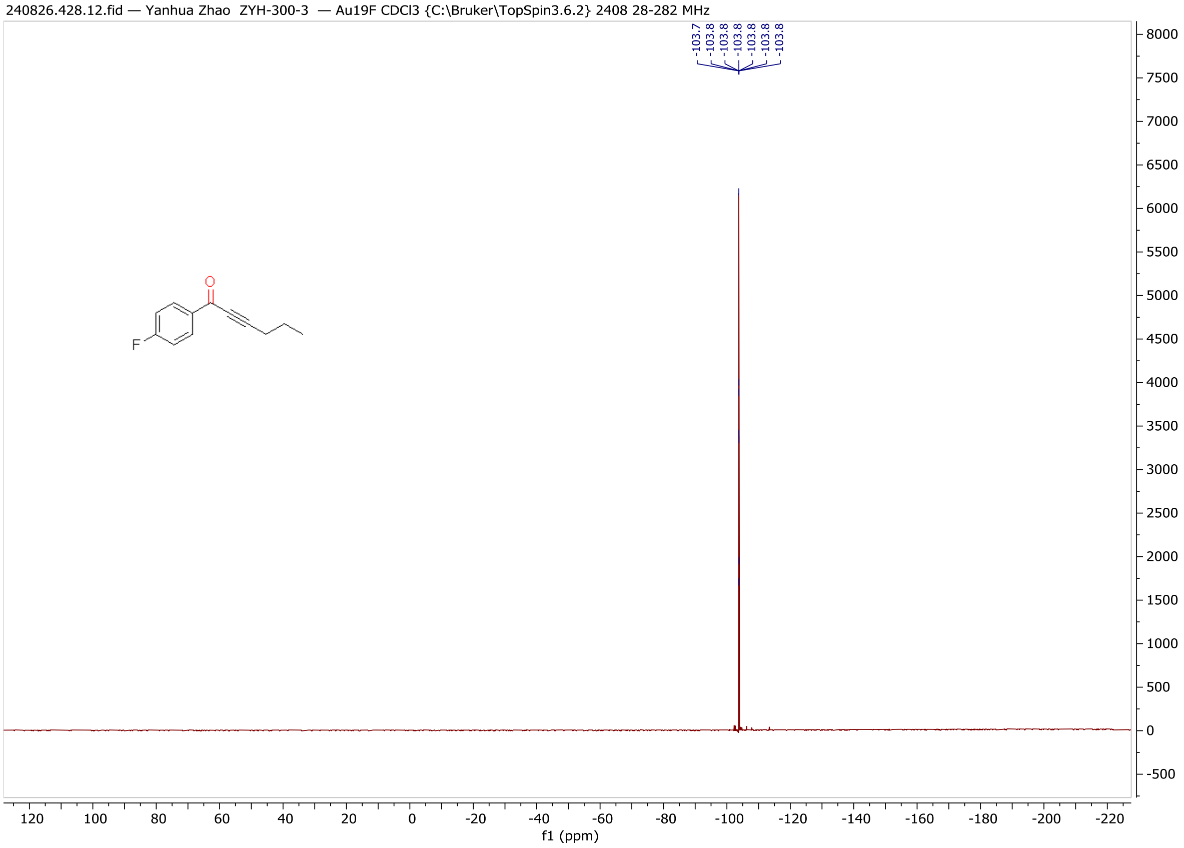

240826.428.12.fid — Yanhua Zhao ZYH-300-3 — Au19F CDCl3 {C:\Bruker\TopSpin3.6.2} 2408 28-282 MHz
-103.7
-103.8
-103.8
-103.8
-103.8
-103.8
-103.8
O
F
8000
7500
7000
6500
6000
5500
5000
4500
4000
3500
3000
2500
2000
1500
1000
500
0
-500
120
100
80
60
40
20
0
-20
-40
-60
-80
-100
-120
-140
-160
-180
-200
-220
f1 (ppm)

240826.429.10.fid — Yanhua Zhao ZYH-300-4 — Au1H CDCl3 {C:\Bruker\TopSpin3.6.2} 2408 29 - 300 MHz

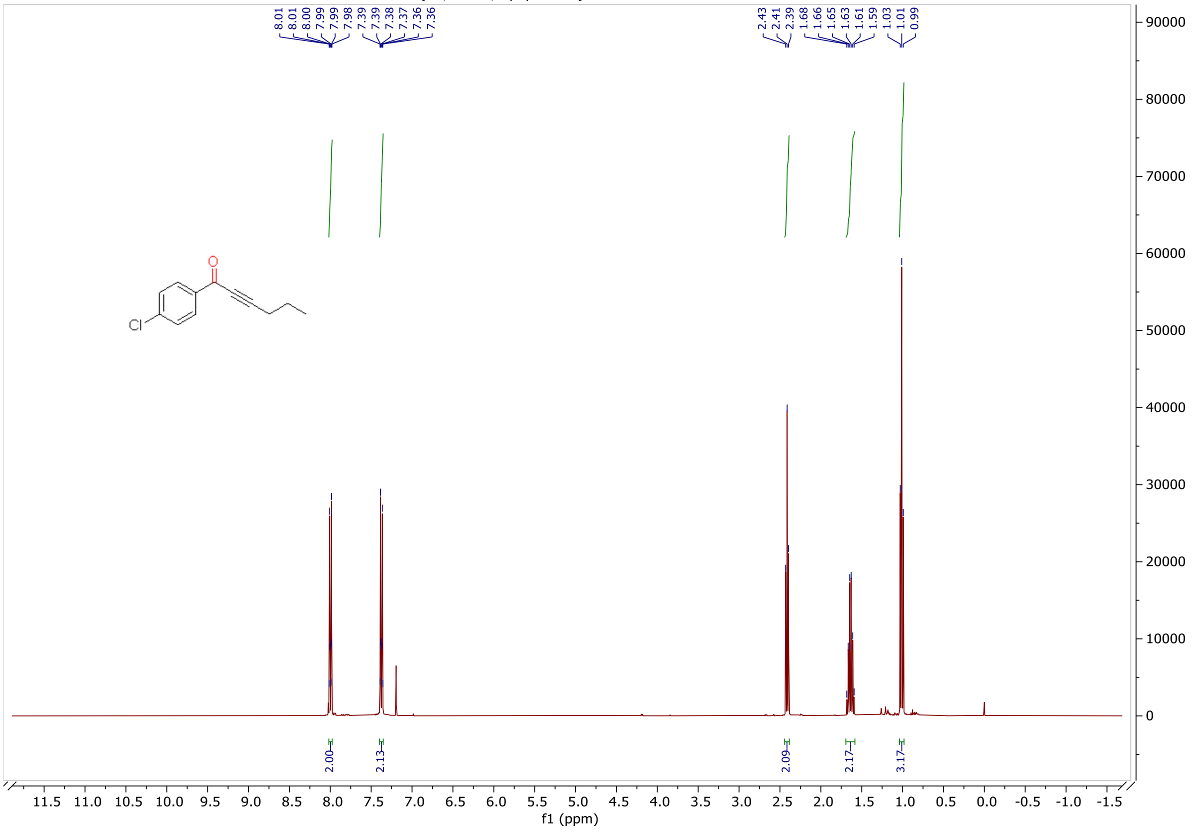

240826.429.11.fid — Yanhua Zhao ZYH-300-4 — Au13C CDCl3 {C:\Bruker\TopSpin3.6.2} 2408 29 - 75 MHz

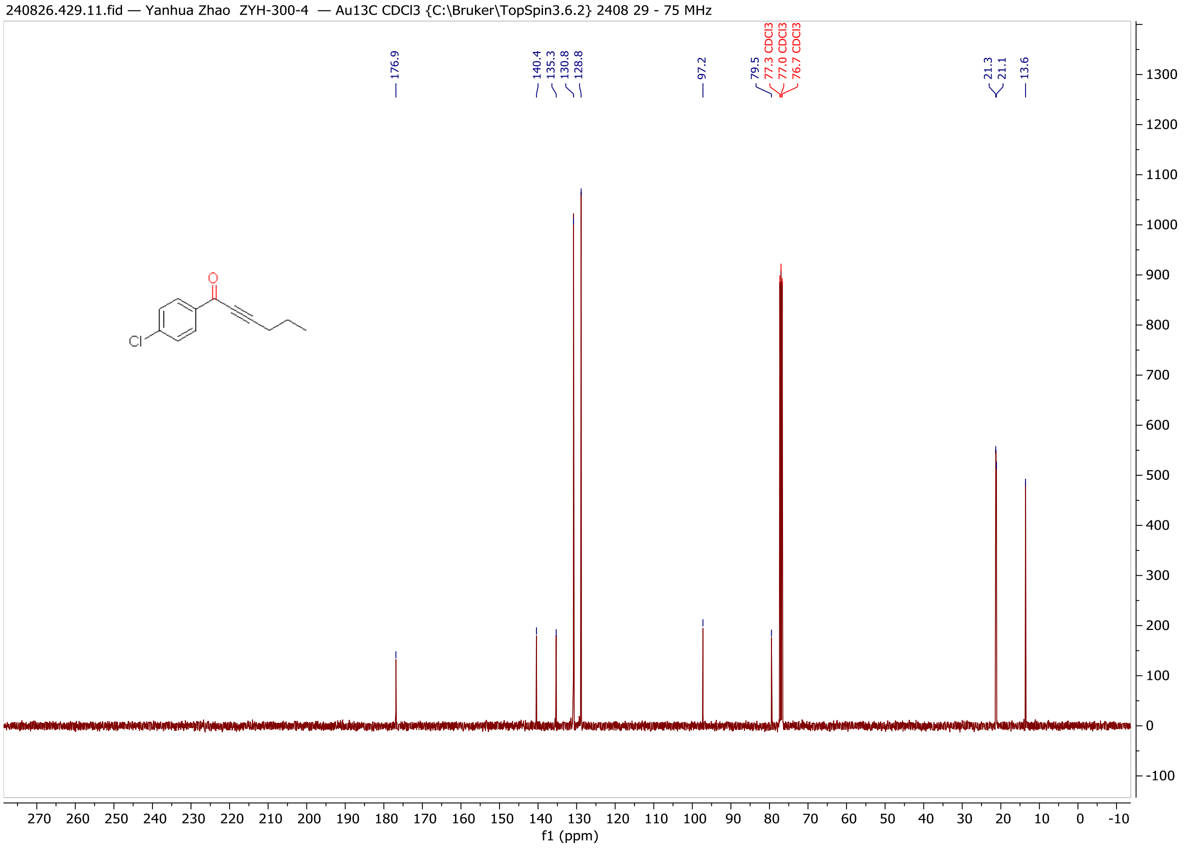

240826.430.10.fid — Yanhua Zhao ZYH-300-5 — Au1H CDCl3 {C:\Bruker\TopSpin3.6.2} 2408 30 - 300 MHz

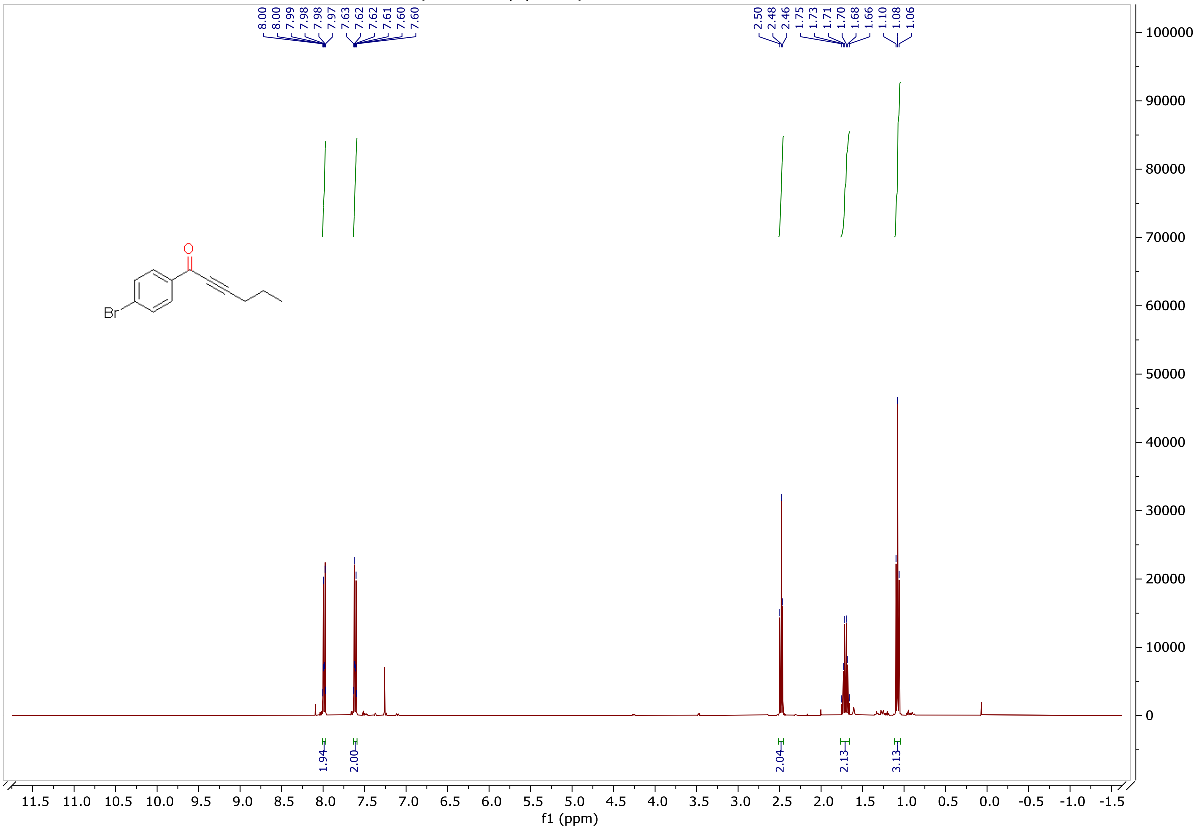

240826.430.11.fid — Yanhua Zhao ZYH-300-5 — Au13C CDCl3 {C:\Bruker\TopSpin3.6.2} 2408 30 - 75 MHz

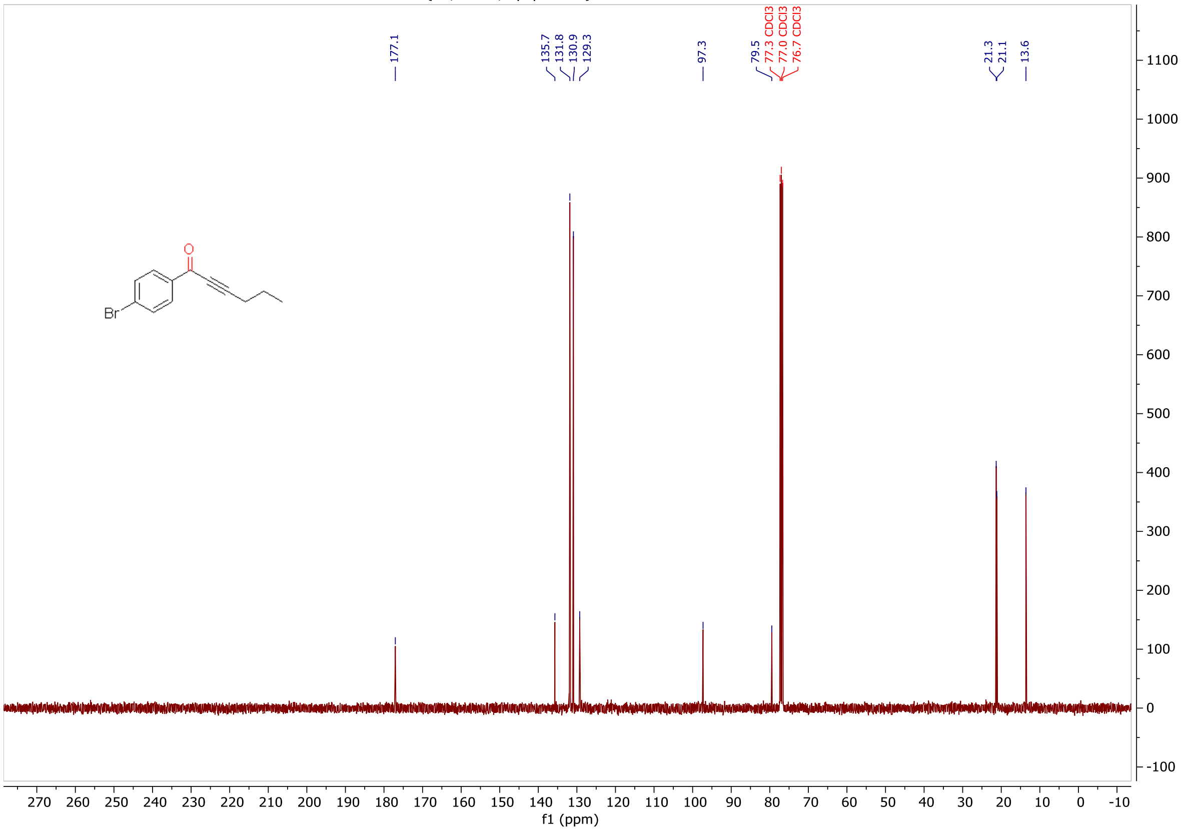

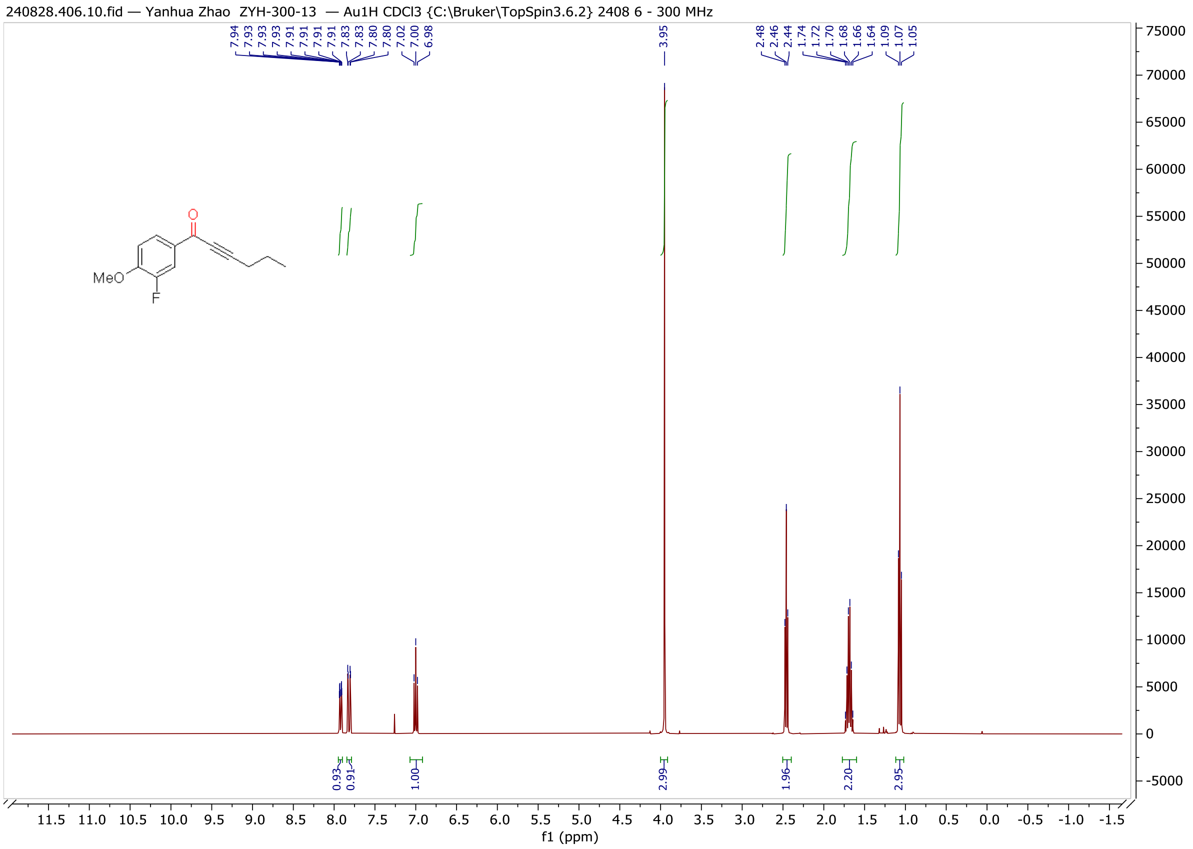
240828.406.10.fid — Yanhua Zhao ZYH-300-13 — Au1H CDCl3 {C:\Bruker\TopSpin3.6.2} 2408 6 - 300 MHz
7.94 7.93 7.93 7.93 7.91 7.91 7.91 7.91 7.83 7.83 7.80 7.80 7.02 7.00 6.98
3.95
2.48 2.46 2.44 1.74 1.72 1.70 1.68 1.66 1.64 1.09 1.07 1.05
MeO
F
O
0.93 0.91 1.00 2.99 1.96 2.20 2.95
f1 (ppm)

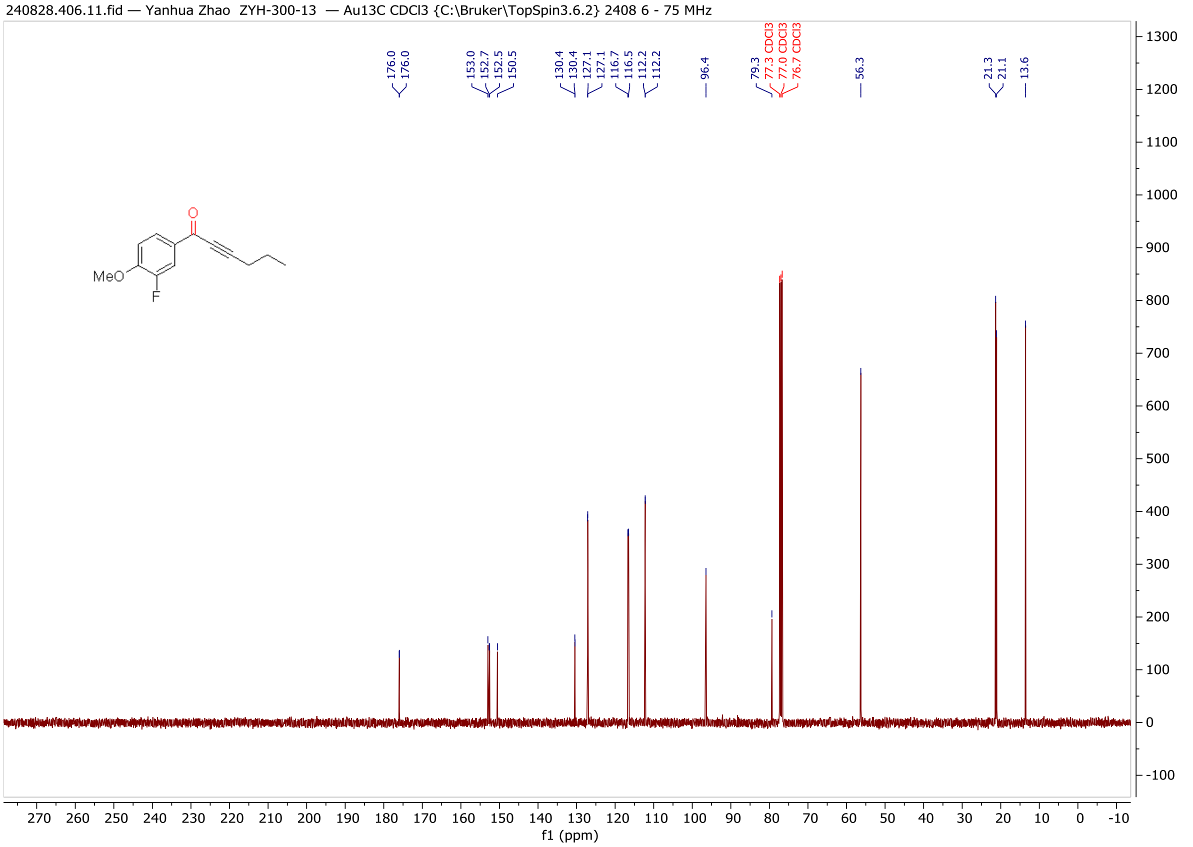
240828.406.11.fid — Yanhua Zhao ZYH-300-13 — Au13C CDCl3 {C:\Bruker\TopSpin3.6.2} 2408 6 - 75 MHz
176.0 176.0
153.0 152.7 152.5 150.5
130.4 130.4 127.1 127.1 116.7 116.5 112.2 112.2
96.4
79.3 77.3 CDCl3 77.0 CDCl3 76.7 CDCl3
56.3
21.3 21.1 13.6
MeO
F
O
f1 (ppm)

240828.406.12.fid — Yanhua Zhao ZYH-300-13 — Au19F CDCl3 {C:\Bruker\TopSpin3.6.2} 2408 6-282 MHz

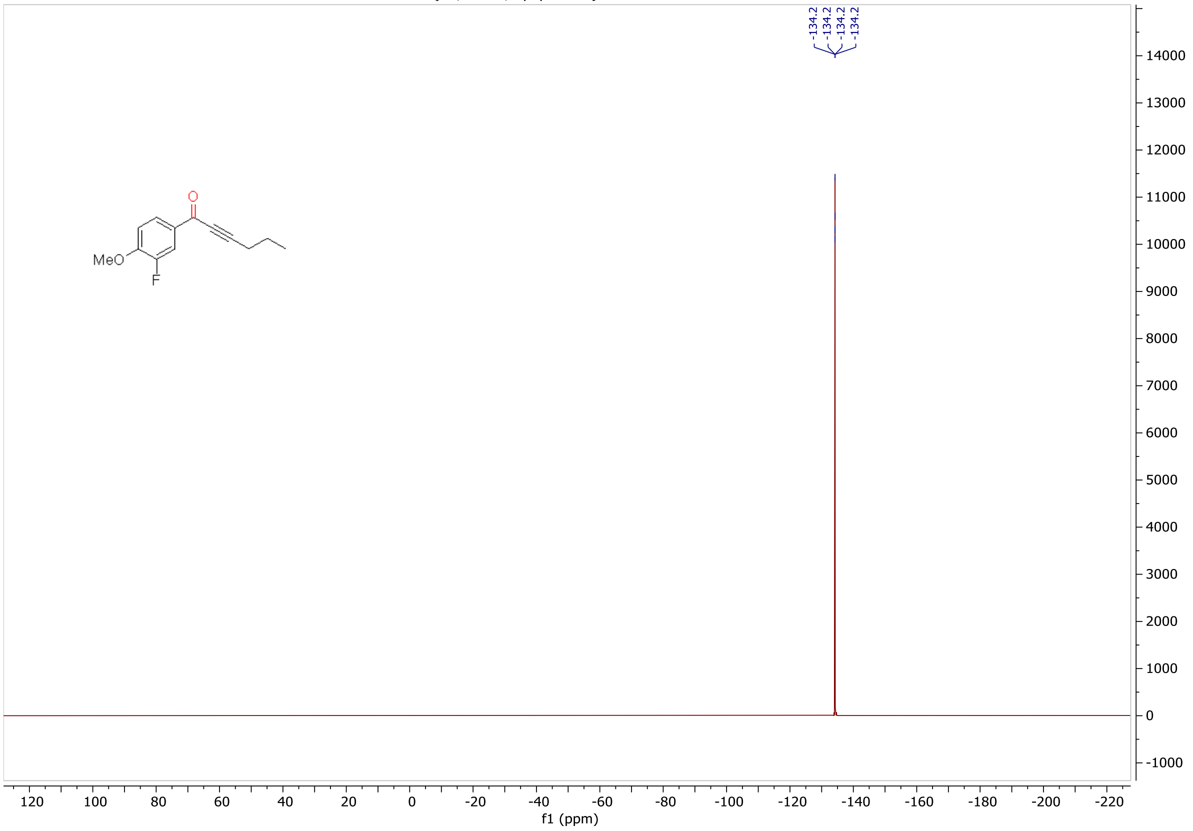

240828.410.10.fid — Yanhua Zhao ZYH-300-12 — Au1H CDCl3 {C:\Bruker\TopSpin3.6.2} 2408 10 - 300 MHz

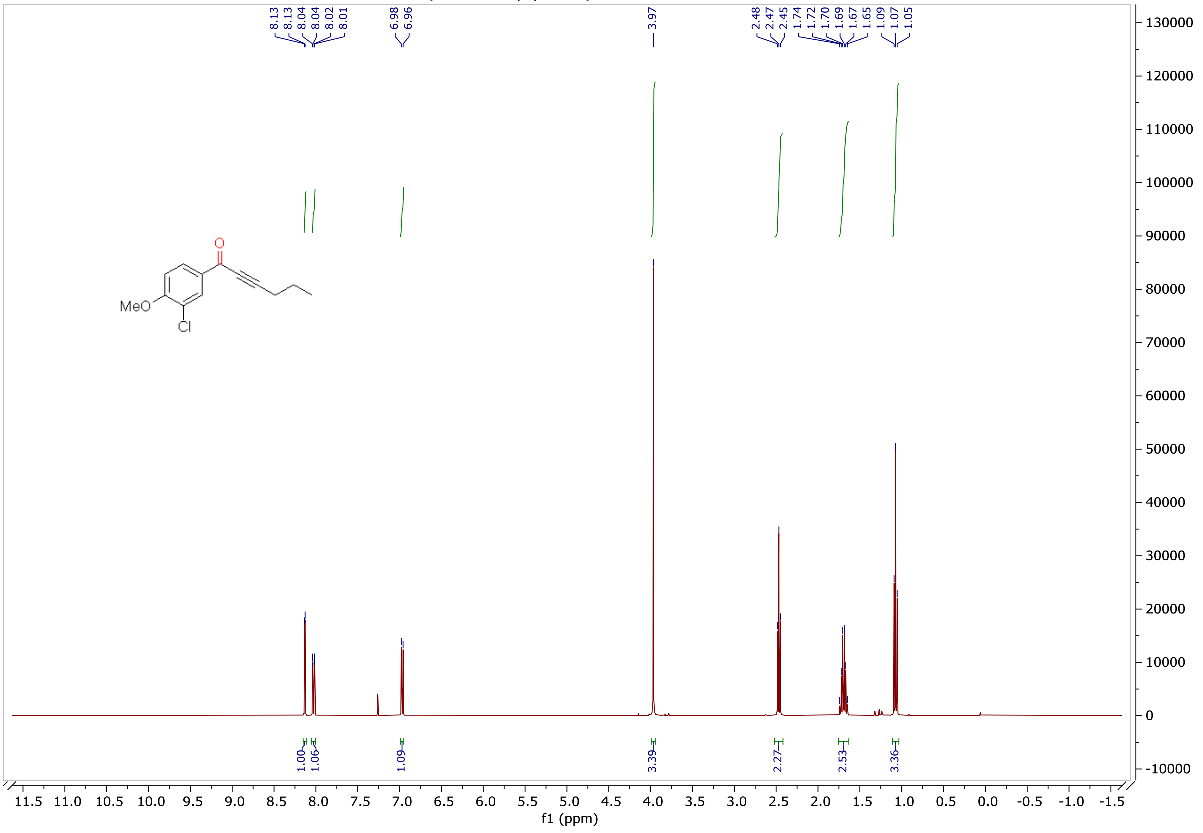

240828.410.11.fid — Yanhua Zhao ZYH-300-12 — Au13C CDCl3 {C:\Bruker\TopSpin3.6.2} 2408 10 - 75 MHz

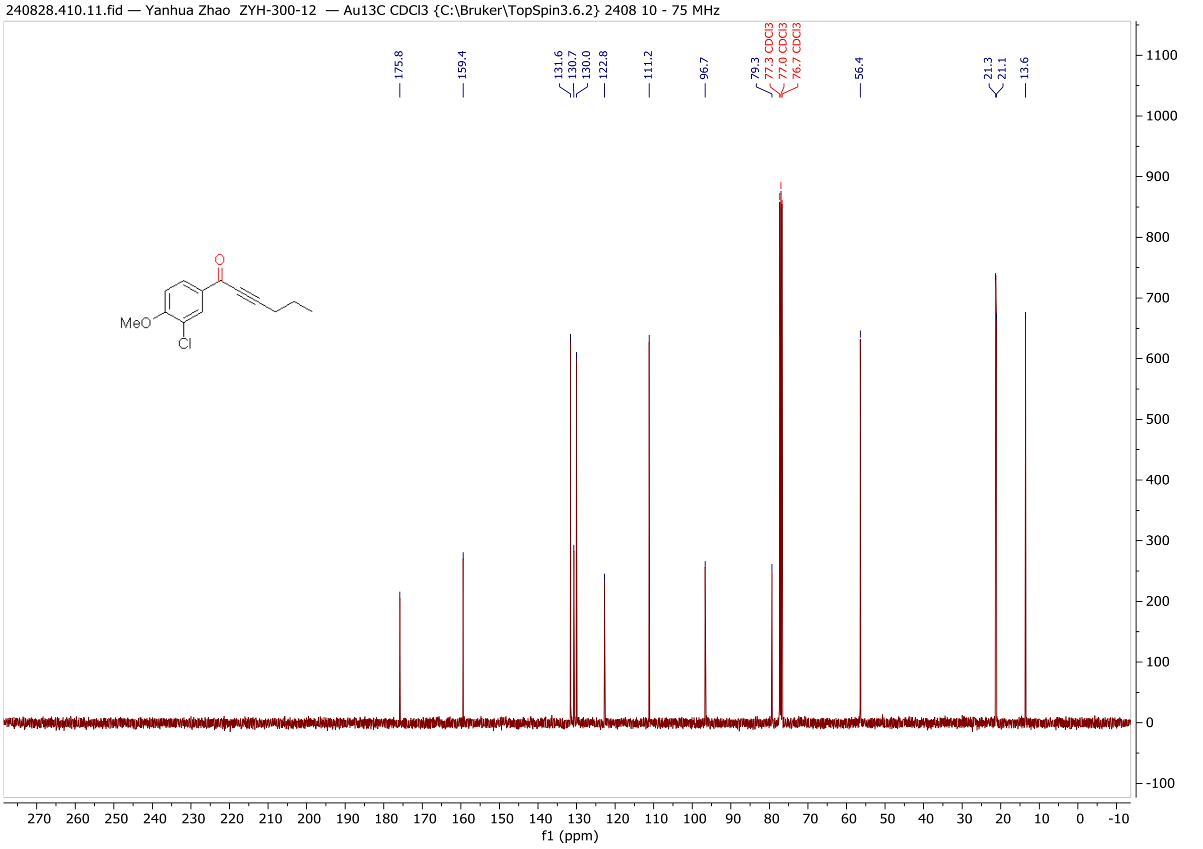

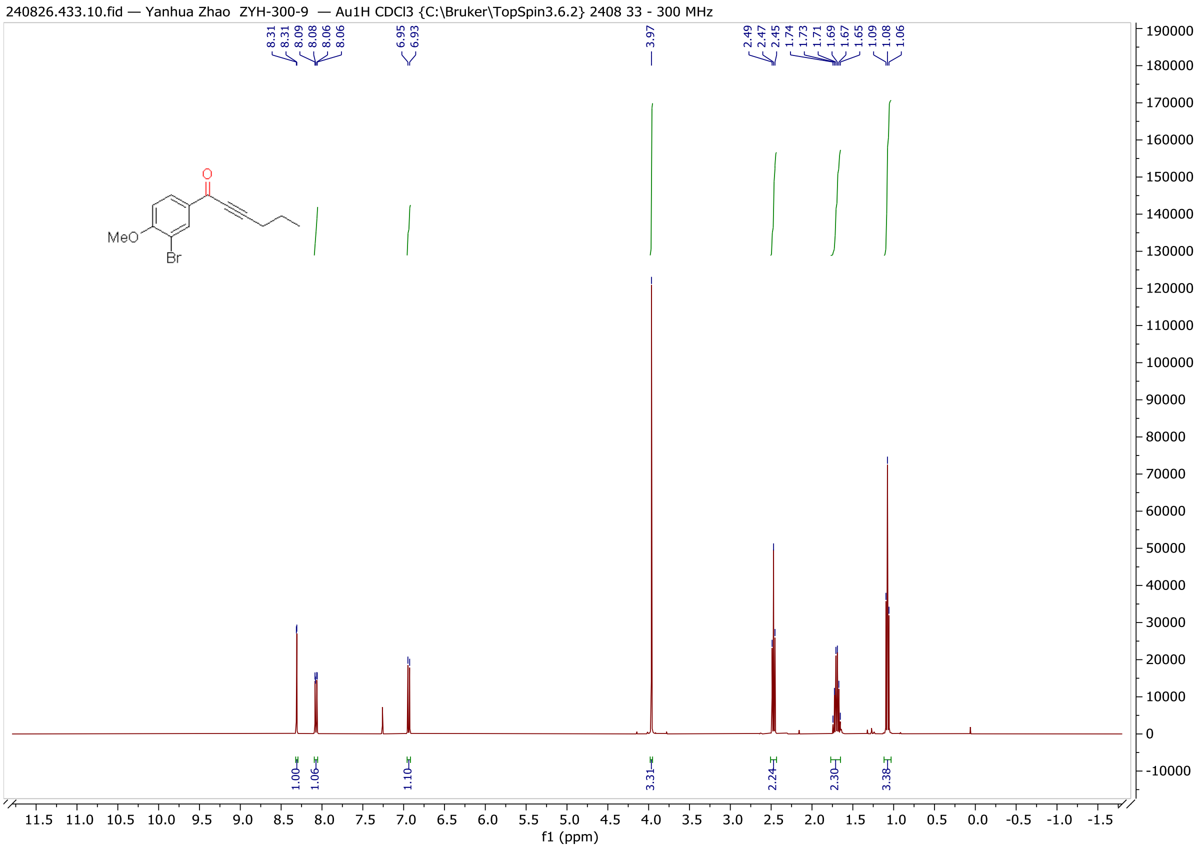
240826.433.10.fid — Yanhua Zhao ZYH-300-9 — Au1H CDCl3 {C:\Bruker\TopSpin3.6.2} 2408 33 - 300 MHz
8.31 8.31 8.09 8.08 8.06 8.06
6.95 6.93
3.97
2.49 2.47 2.45 1.74 1.73 1.71 1.69 1.67 1.65 1.09 1.08 1.06
MeO
Br
O
1.00 1.06 1.10 3.31 2.24 2.30 3.38
f1 (ppm)

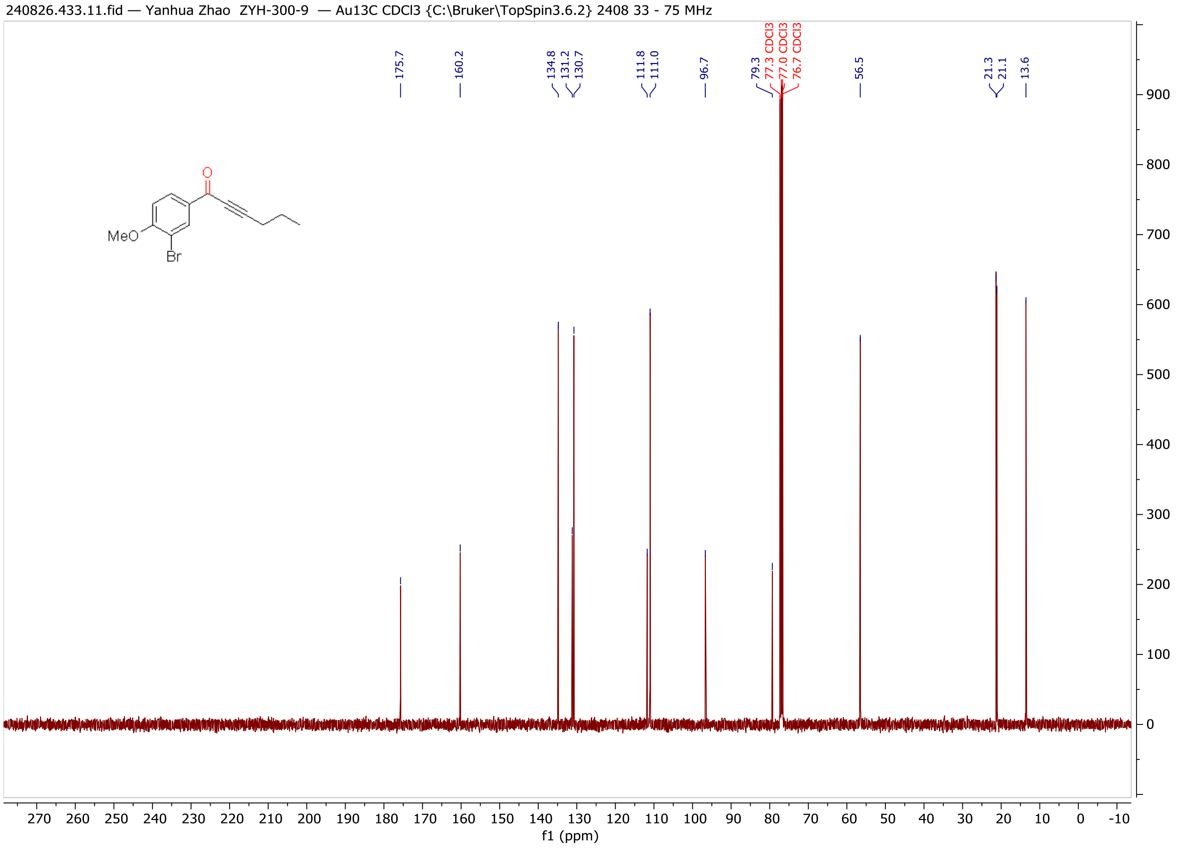
240826.433.11.fid — Yanhua Zhao ZYH-300-9 — Au13C CDCl3 {C:\Bruker\TopSpin3.6.2} 2408 33 - 75 MHz
175.7
160.2
134.8 131.2 130.7
111.8 111.0
96.7
79.3 77.3 CDCl3 77.0 CDCl3 76.7 CDCl3
56.5
21.3 21.1
13.6
MeO
Br
O
f1 (ppm)

240830.f308.10.fid — Yanhua Zhao ZYH-300-16 — Au1H CDCl3 {C:\Bruker\TopSpin3.6.2} 2408 8 - 300 MHz

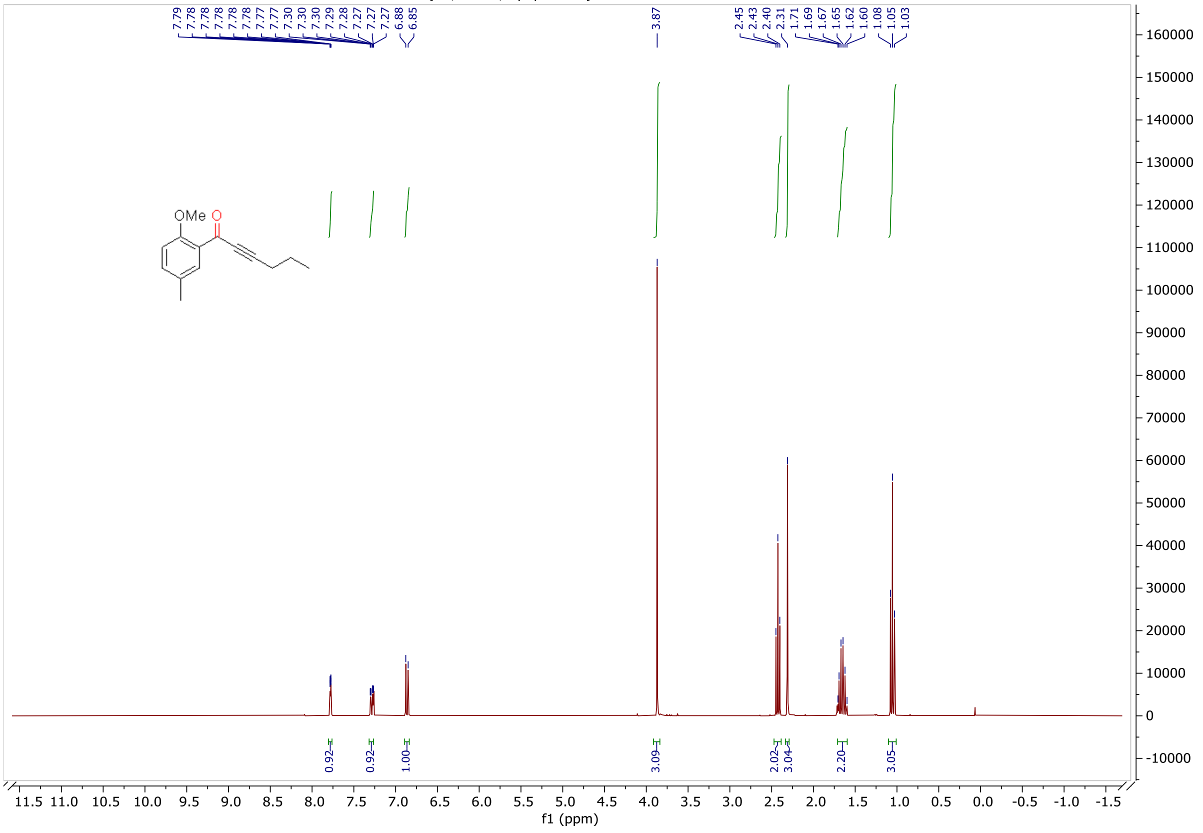

240830.f308.11.fid — Yanhua Zhao ZYH-300-16 — Au13C CDCl3 {C:\Bruker\TopSpin3.6.2} 2408 8 - 75 MHz

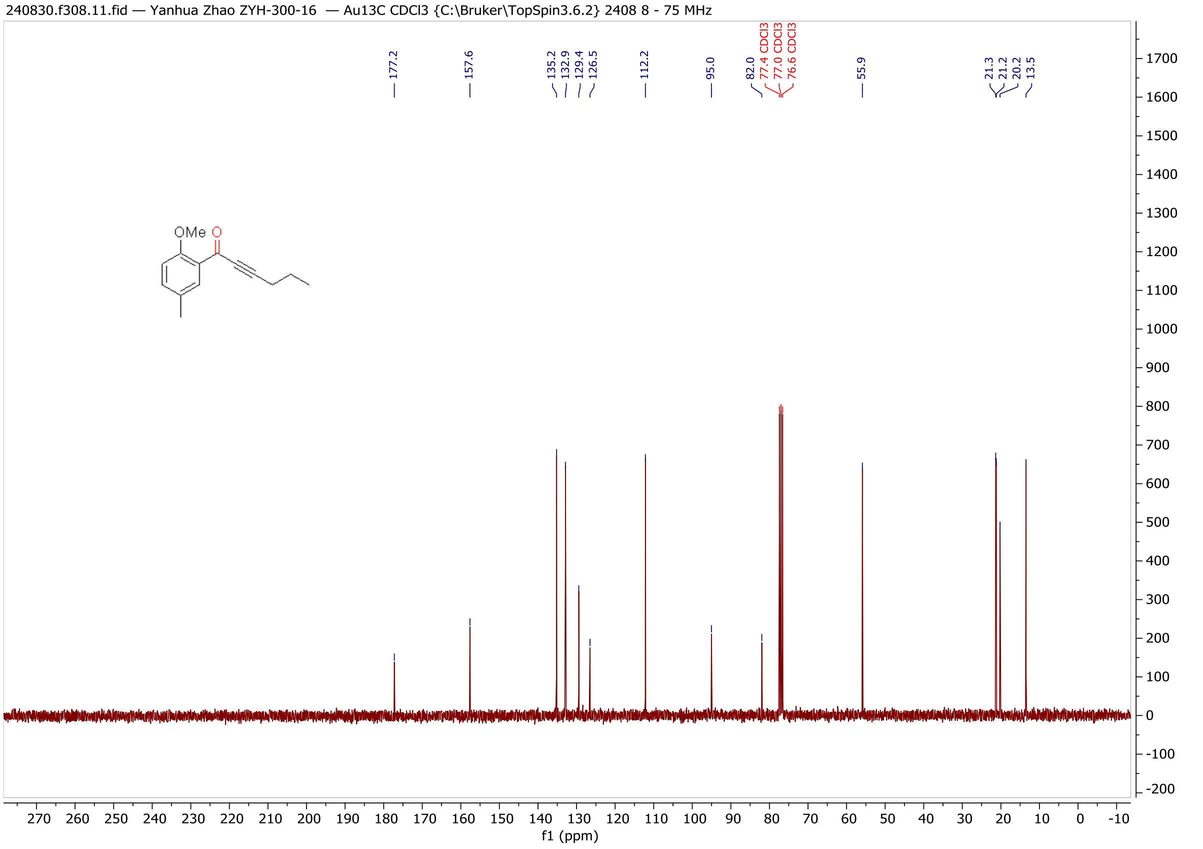

240917.f311.4.fid — Yanhua Zhao ZYH-302-1 — Au1H CDCl3 {C:\Bruker\TopSpin3.6.2} 2409 11 - 300 MHz

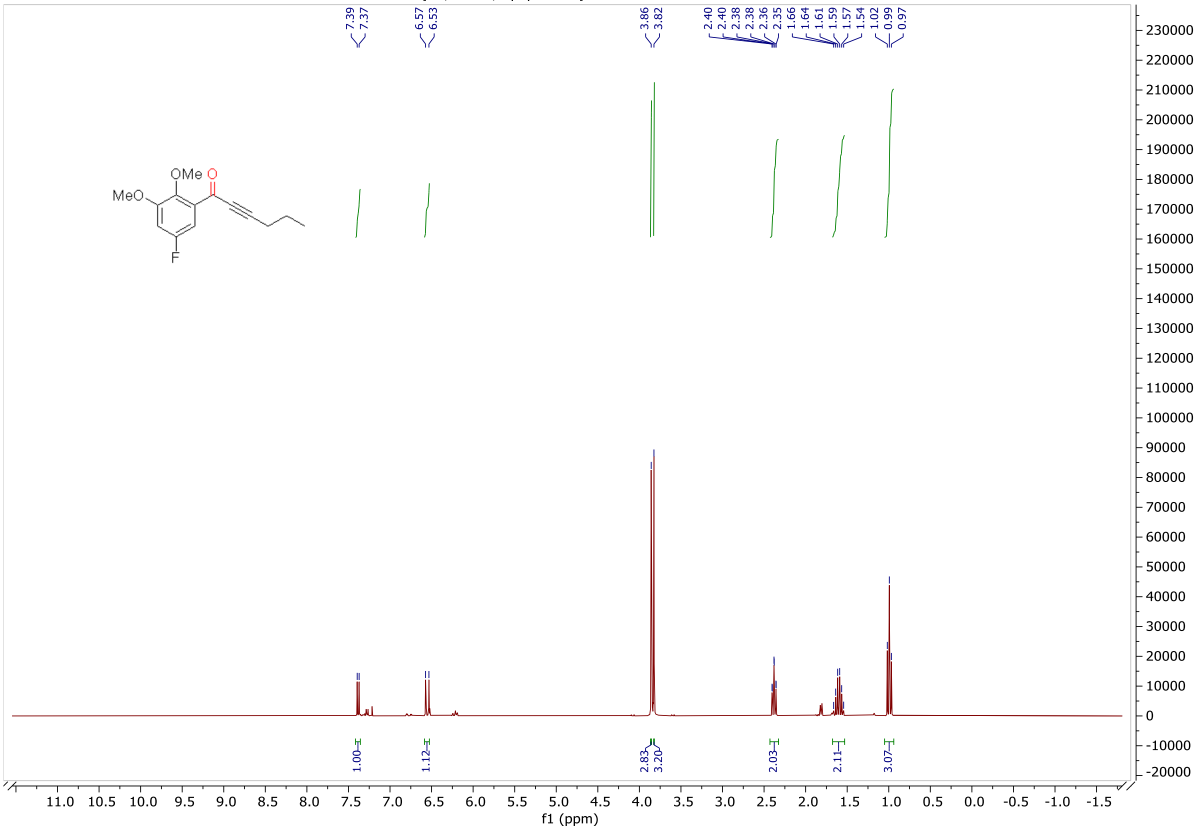

240917.f311.11.fid — Yanhua Zhao ZYH-302-1 — Au13C CDCl3 {C:\Bruker\TopSpin3.6.2} 2409 11 - 75 MHz

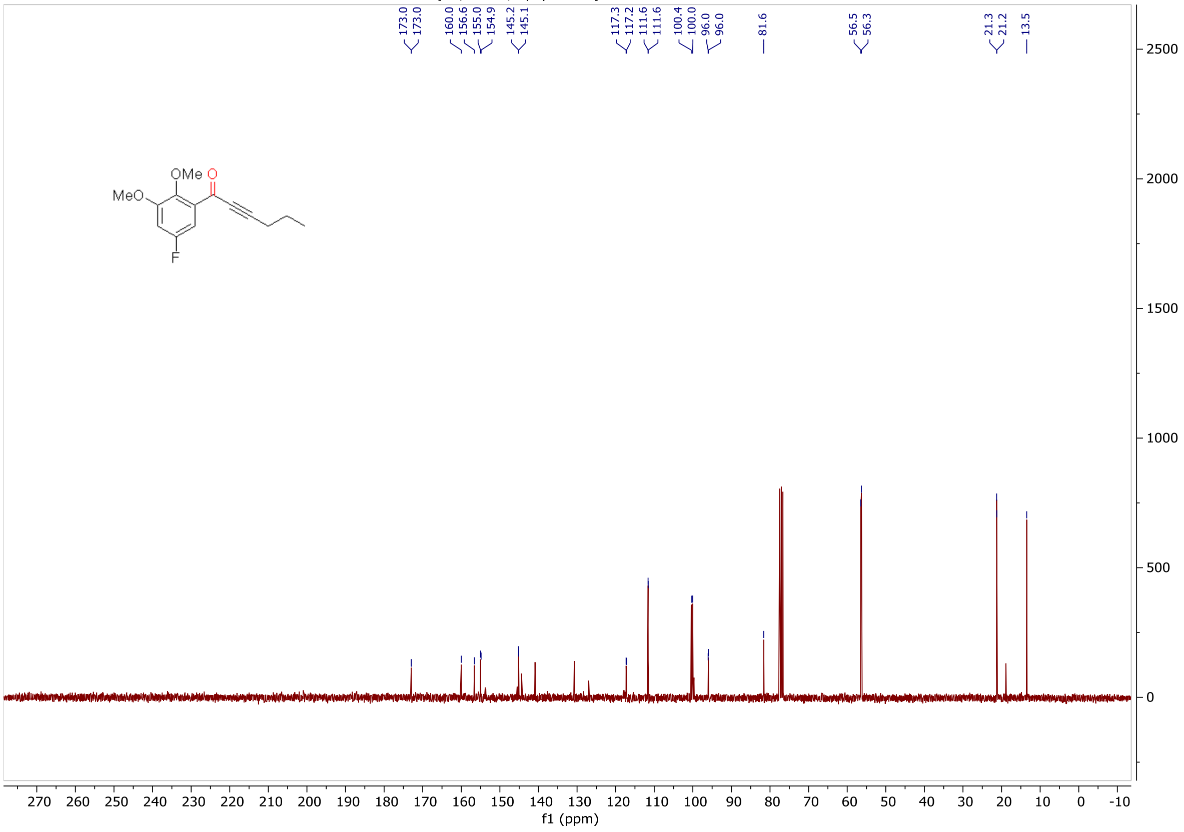

240917.f311.12.fid — Yanhua Zhao ZYH-302-1 — Au19F(H-gek) CDCl3 {C:\Bruker\TopSpin3.6.2} 2409 11-282 MHz

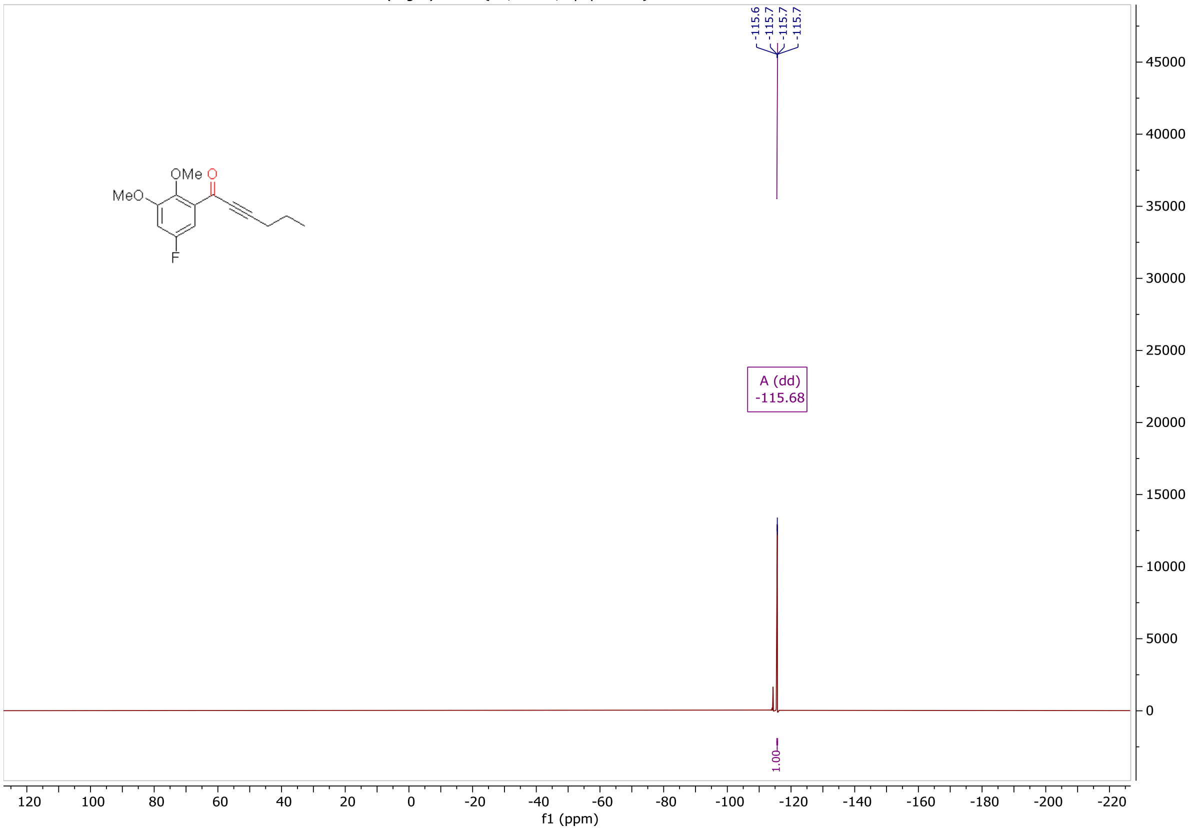

240813.f302.10.fid — Yanhua Zhao ZYH-291-6 — Au1H CDCl3 {C:\Bruker\TopSpin3.6.2} 2408 2 - 300 MHz

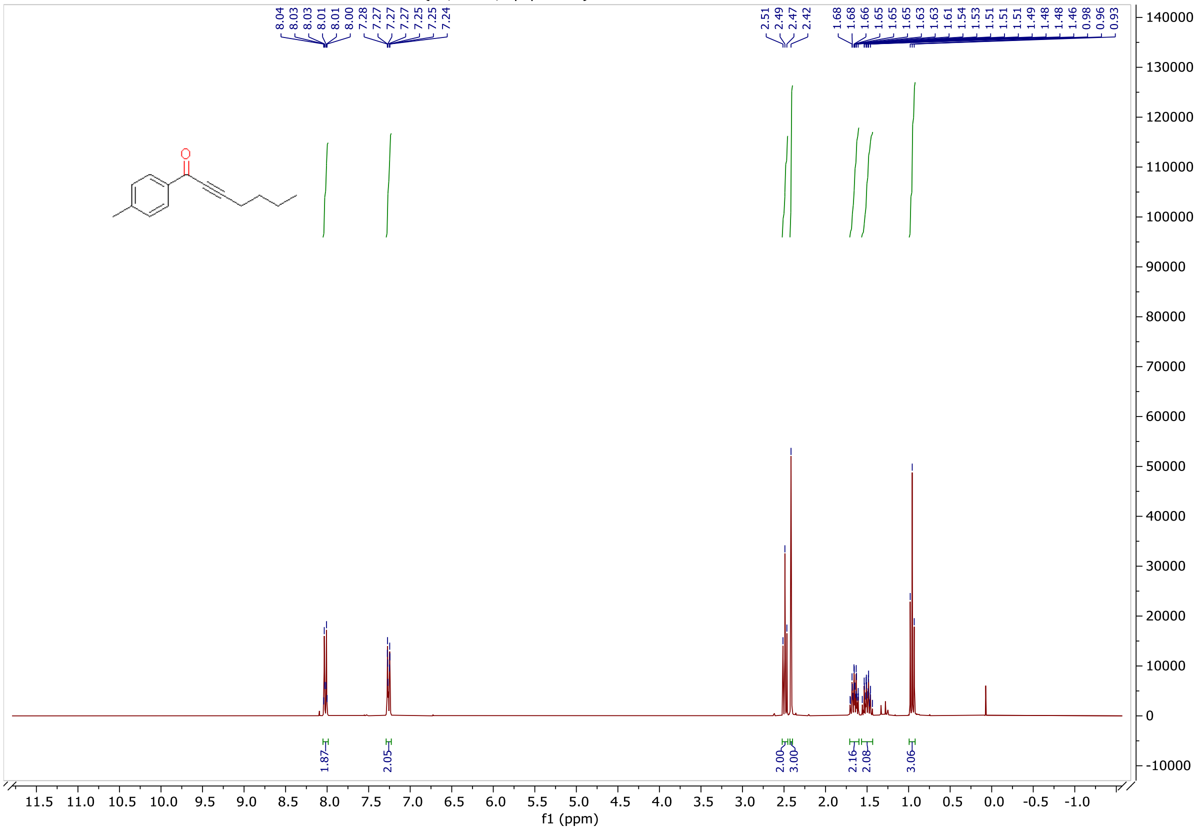

240813.f302.11.fid — Yanhua Zhao ZYH-291-6 — Au13C CDCl3 {C:\Bruker\TopSpin3.6.2} 2408 2 - 75 MHz

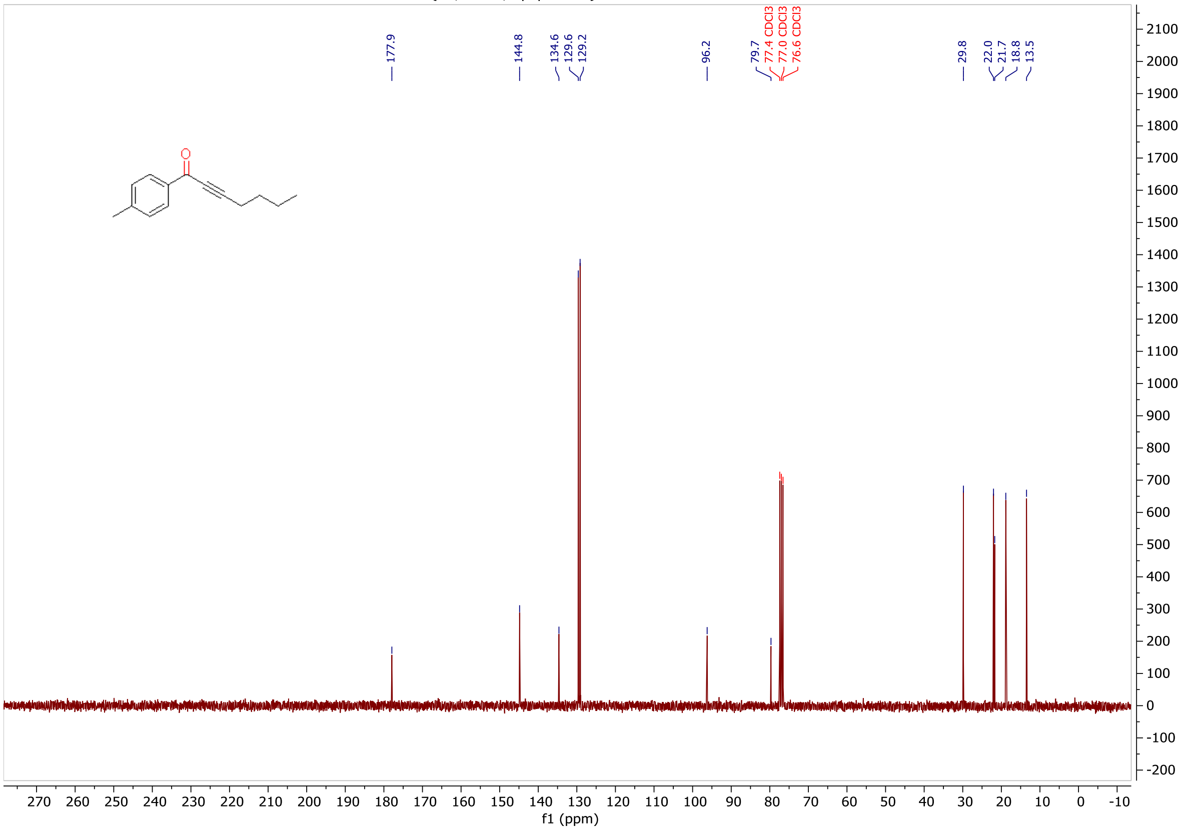

240603.401.10.fid — Yanhua Zhao ZYH-259-1 — Au1H CDCl3 {C:\Bruker\TopSpin3.6.2} 2406 1 - 300 MHz

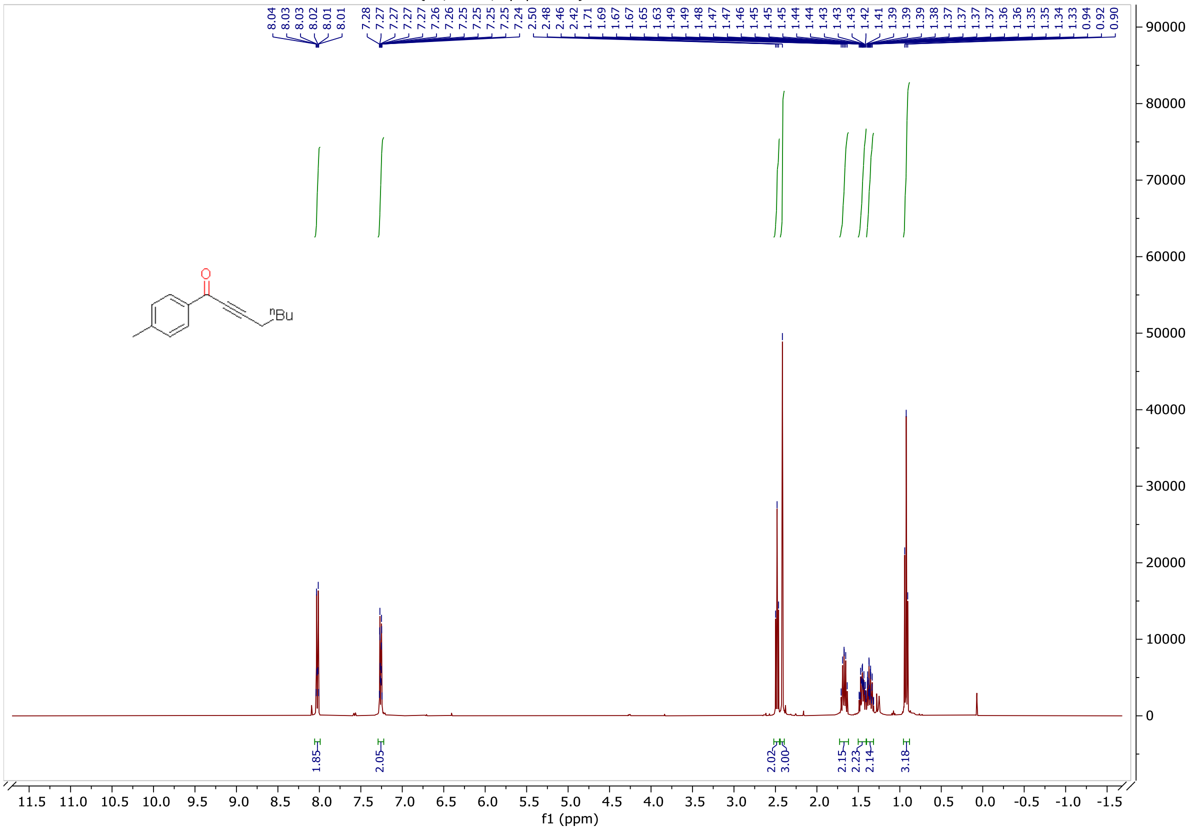

240603.401.11.fid — Yanhua Zhao ZYH-259-1 — Au13C CDCl3 {C:\Bruker\TopSpin3.6.2} 2406 1 - 300 MHz

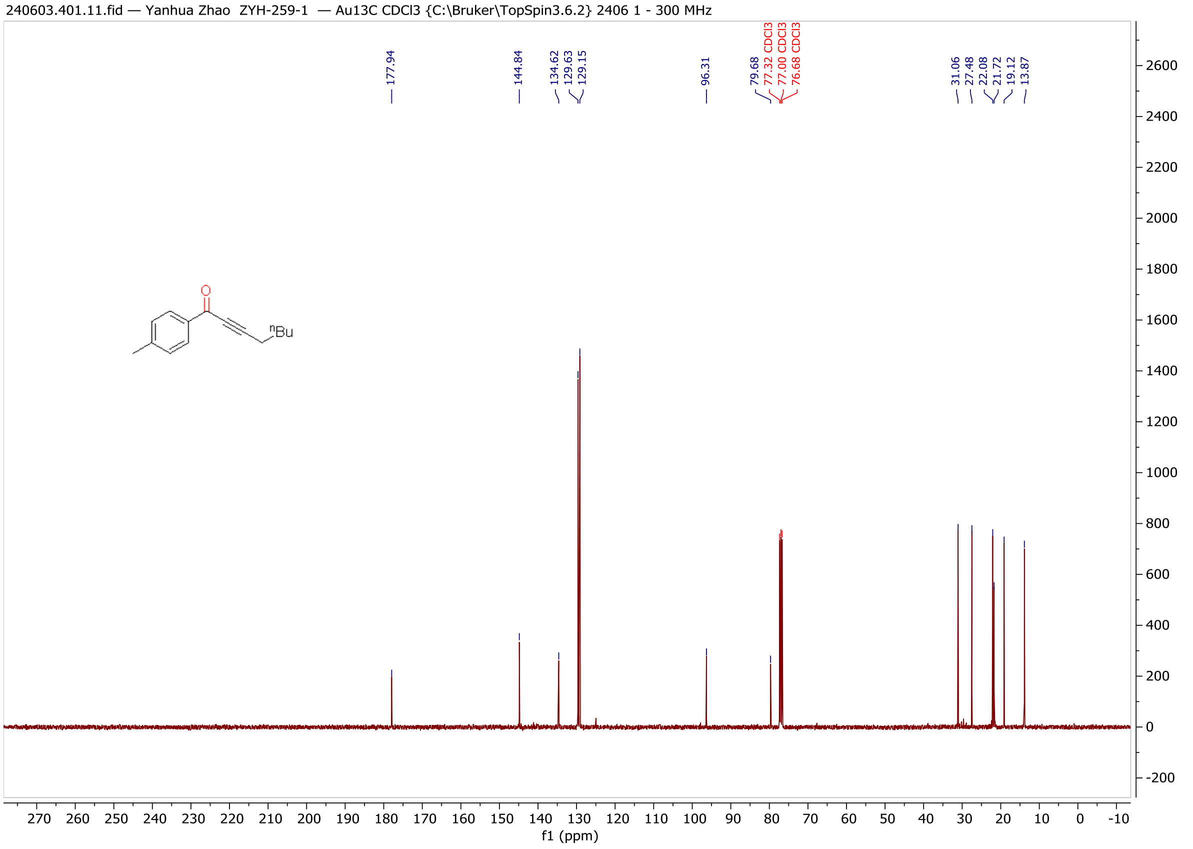

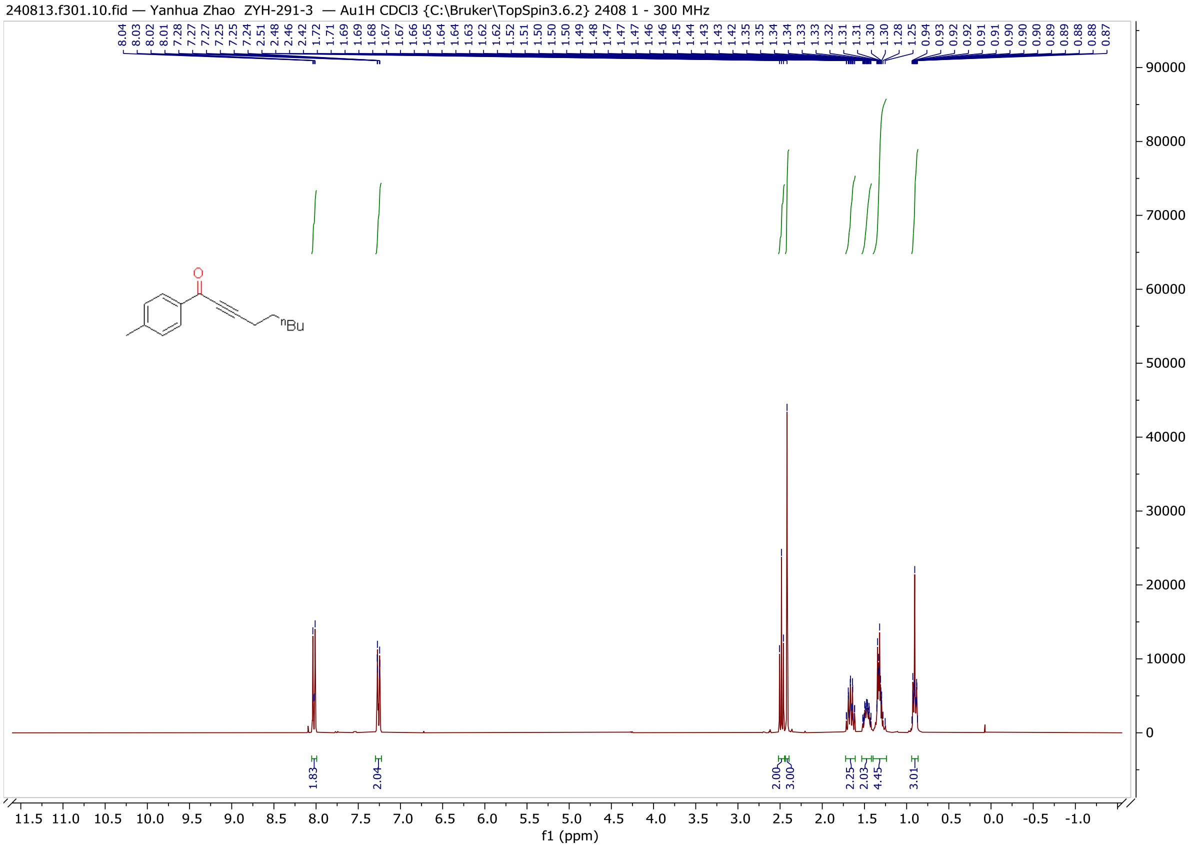
240813.f301.10.fid — Yanhua Zhao ZYH-291-3 — Au1H CDCl3 {C:\Bruker\TopSpin3.6.2} 2408 1 - 300 MHz
8.04 8.03 8.02 8.01 7.28 7.27 7.27 7.25 7.25 7.24 2.51 2.48 2.46 2.42 1.72 1.71 1.69 1.69 1.68 1.67 1.67 1.66 1.65 1.64 1.64 1.63 1.62 1.52 1.51 1.50 1.50 1.50 1.49 1.48 1.47 1.47 1.47 1.46 1.46 1.45 1.44 1.43 1.43 1.42 1.35 1.35 1.34 1.34 1.33 1.33 1.32 1.31 1.31 1.30 1.30 1.28 1.25 0.94 0.93 0.92 0.92 0.91 0.91 0.90 0.90 0.90 0.89 0.88 0.88 0.87
O
nBu
1.83
2.04
2.00
3.00
2.25
2.03
4.45
3.01
f1 (ppm)

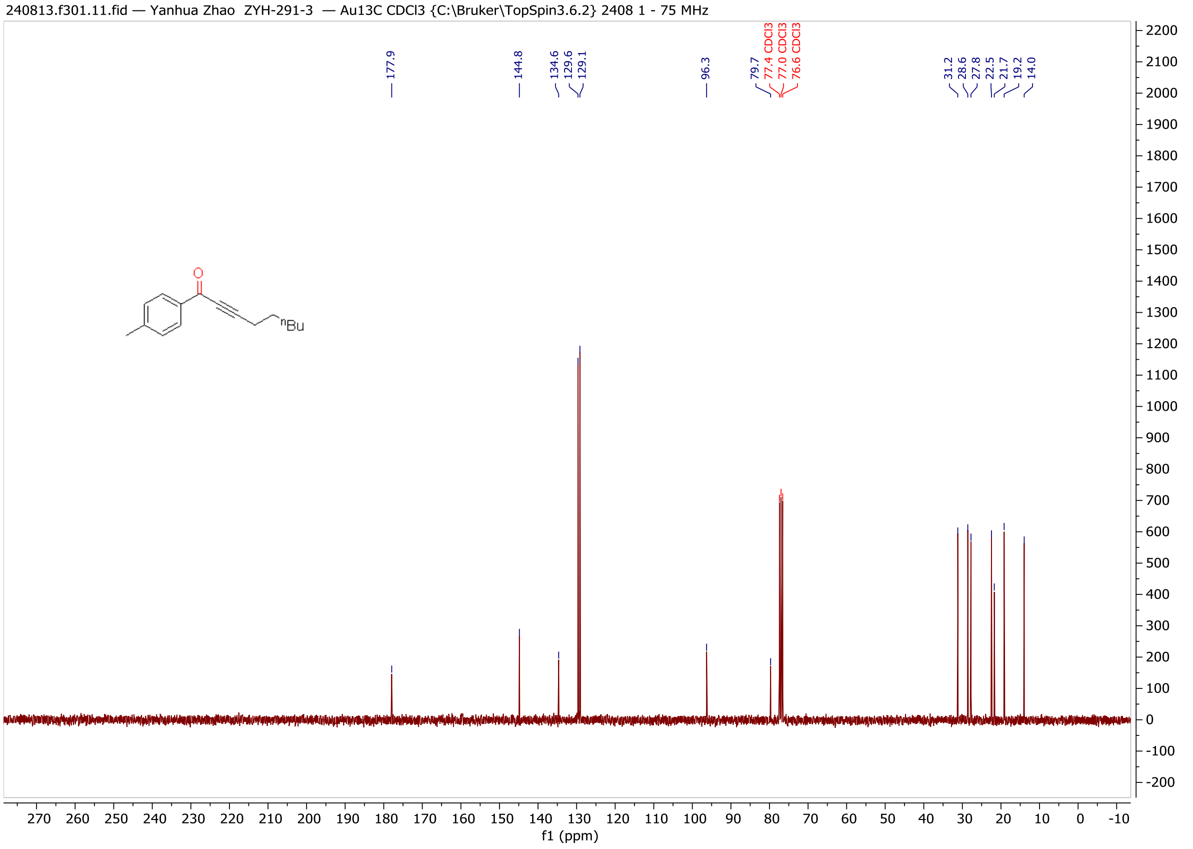
240813.f301.11.fid — Yanhua Zhao ZYH-291-3 — Au13C CDCl3 {C:\Bruker\TopSpin3.6.2} 2408 1 - 75 MHz
177.9
144.8
134.6
129.6
129.1
96.3
79.7
77.4 CDCl3
77.0 CDCl3
76.6 CDCl3
31.2
28.6
27.8
22.5
21.7
19.2
14.0
O
nBu
f1 (ppm)

240809.409.10.fid — Yanhua Zhao ZYH-291-1 — Au1H CDCl3 {C:\Bruker\TopSpin3.6.2} 2408 9 - 300 MHz

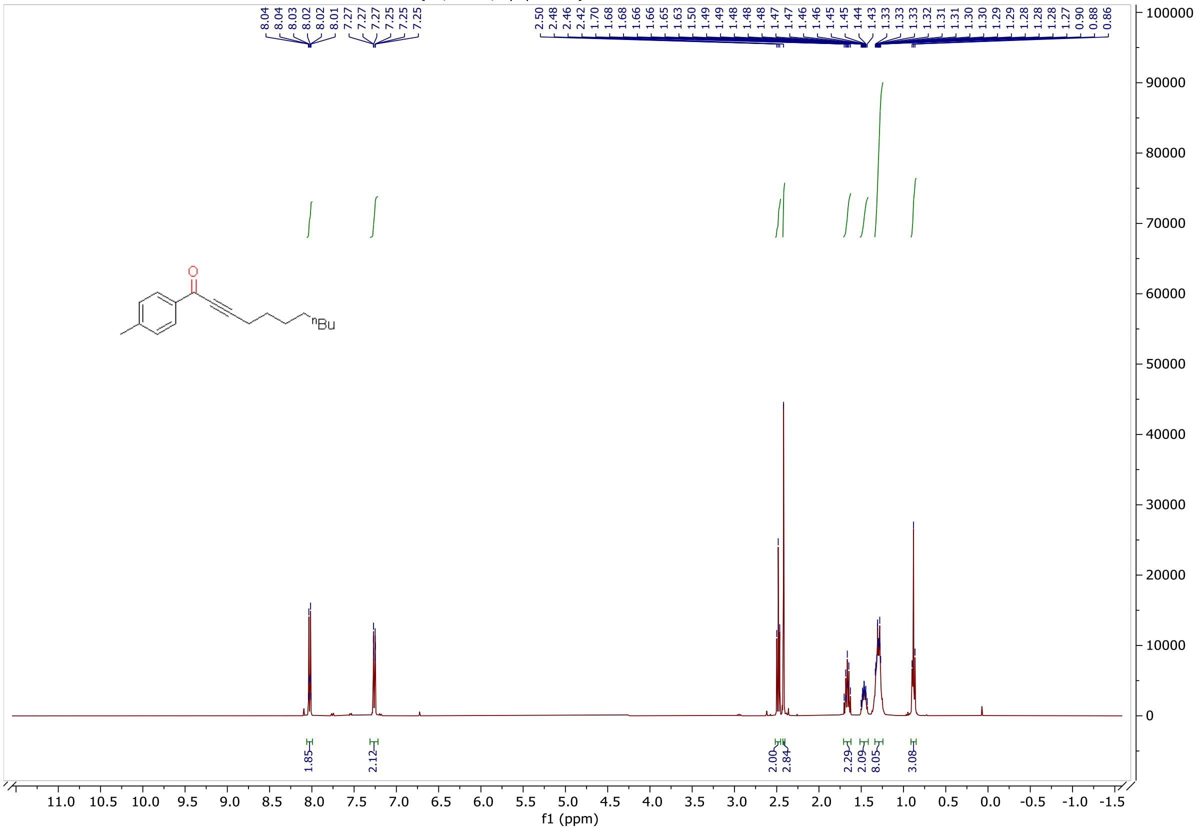

240809.409.11.fid — Yanhua Zhao ZYH-291-1 — Au13C CDCl3 {C:\Bruker\TopSpin3.6.2} 2408 9 - 75 MHz

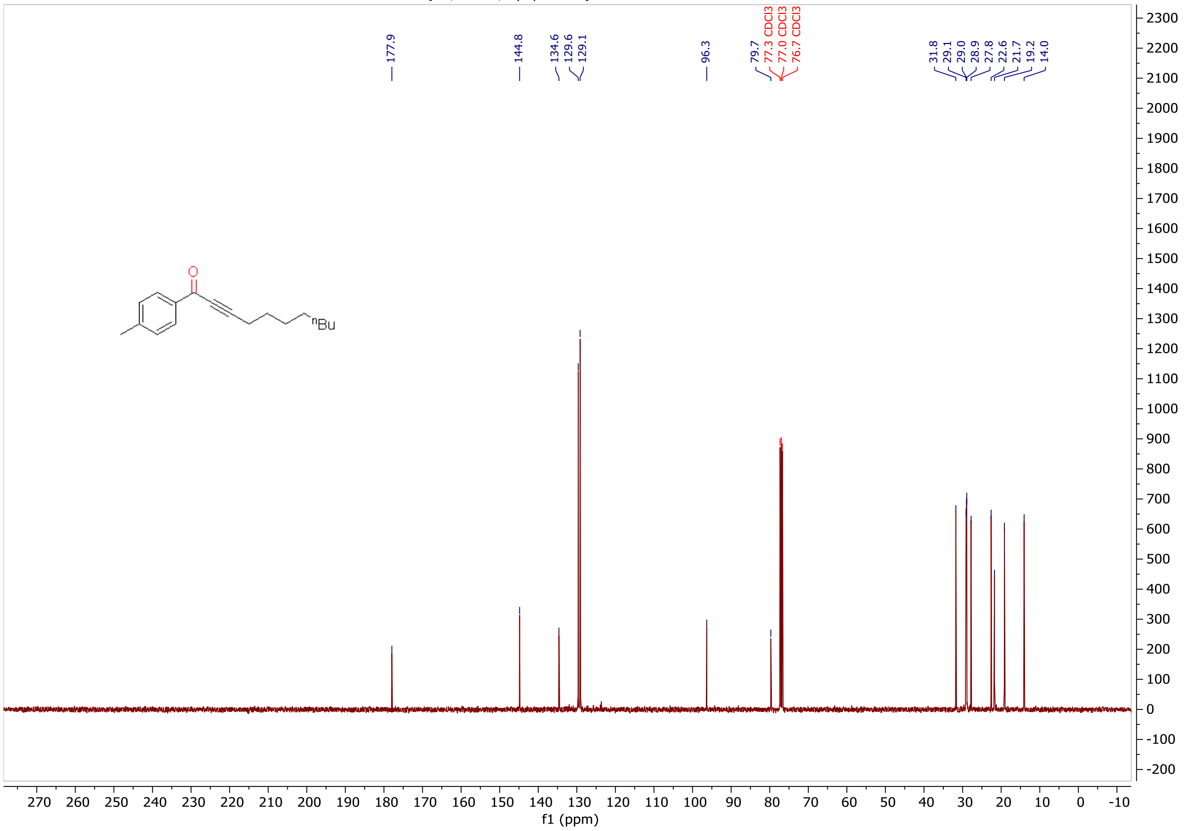

240904.414.10.fid — Yanhua Zhao ZYH-301-4 — Au1H CDCl3 {C:\Bruker\TopSpin3.6.2} 2409 14 - 300 MHz

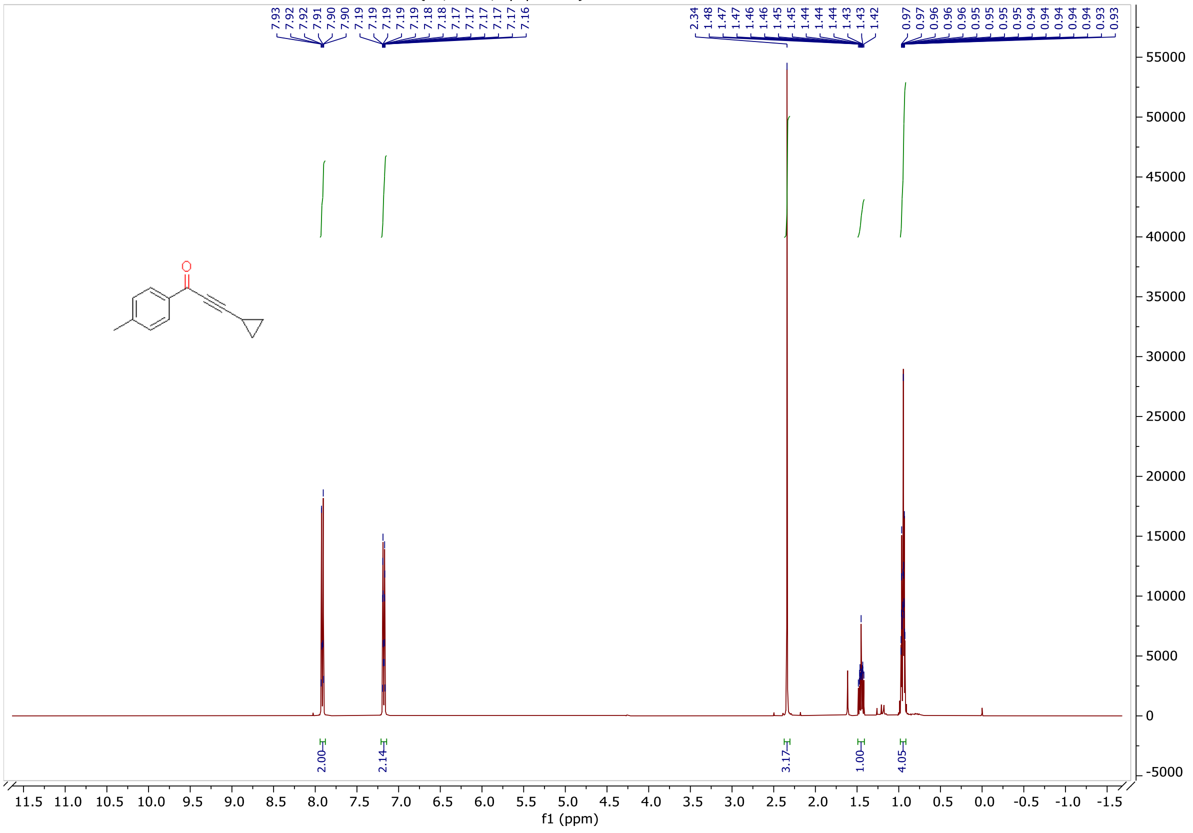

240904.414.11.fid — Yanhua Zhao ZYH-301-4 — Au13C CDCl3 {C:\Bruker\TopSpin3.6.2} 2409 14 - 75 MHz

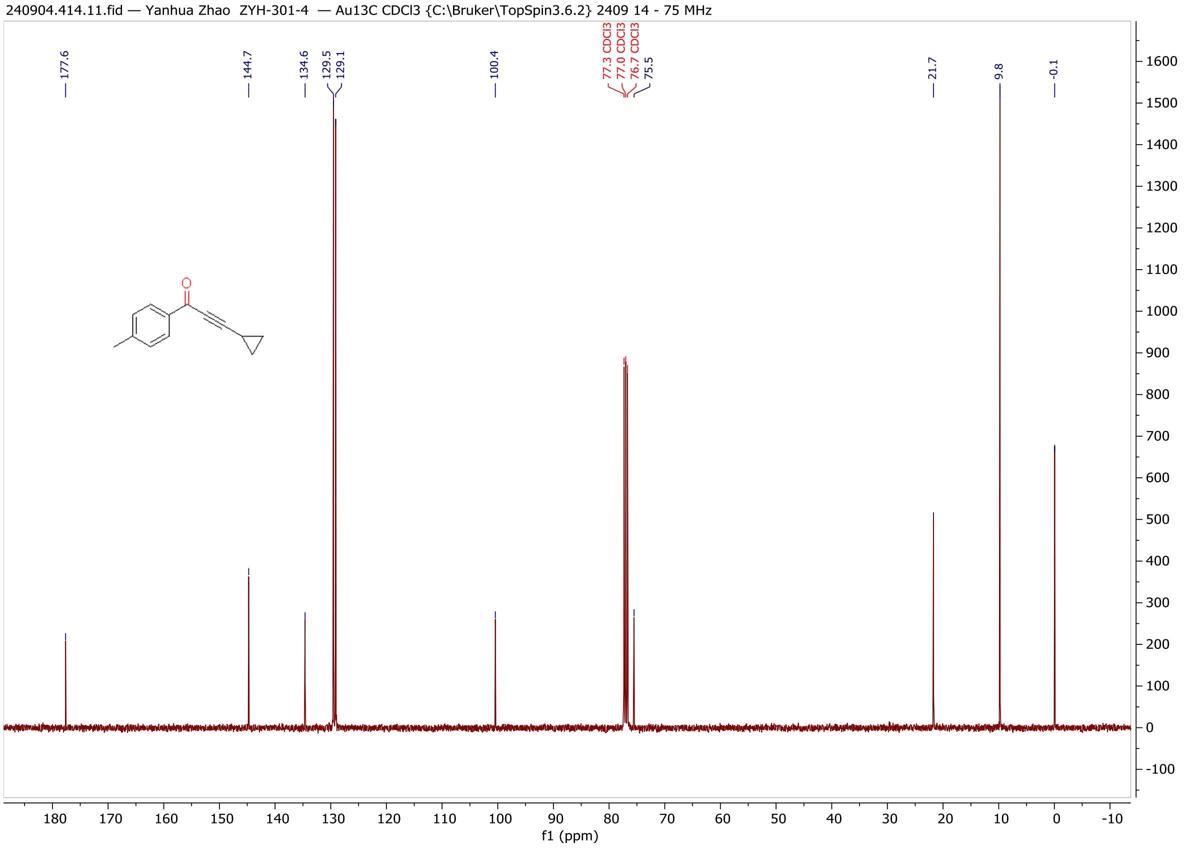

240823.410.10.fid — Yanhua Zhao ZYH-301-1 — Au1H CDCl3 {C:\Bruker\TopSpin3.6.2} 2408 10 - 300 MHz

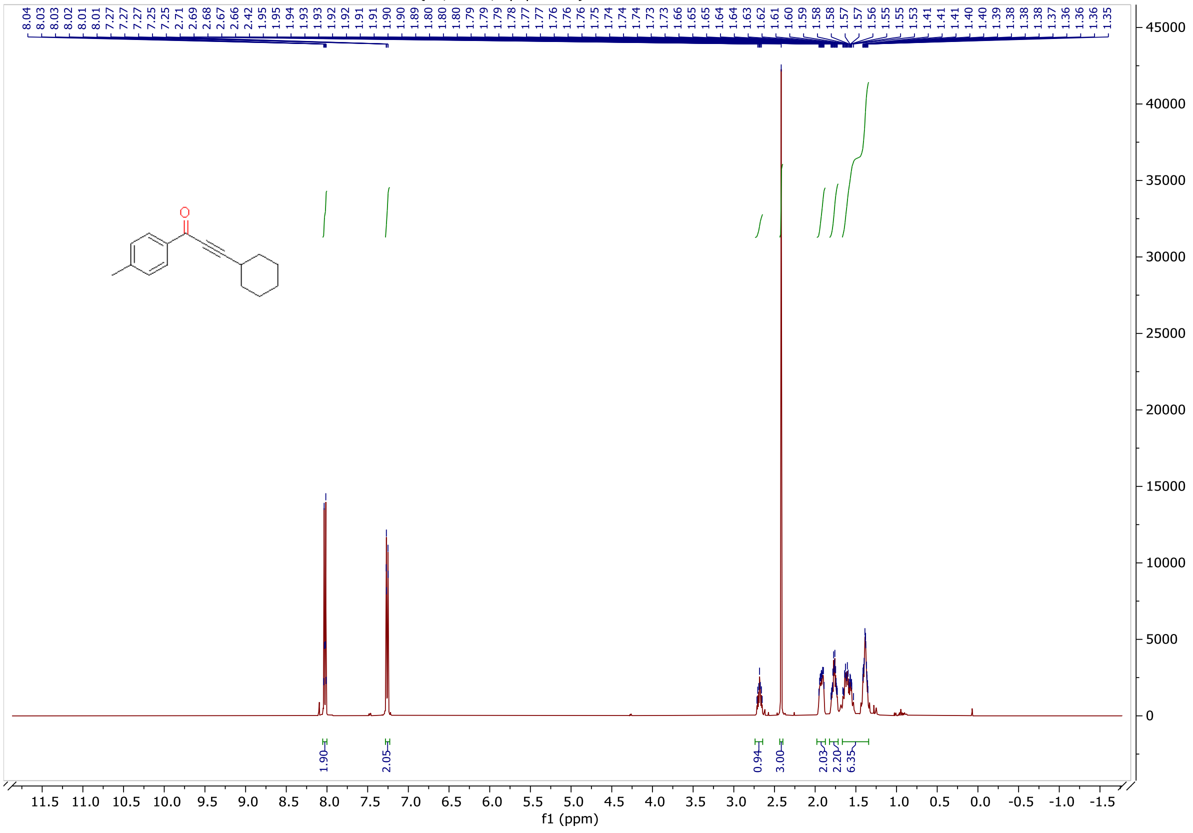

240823.410.11.fid — Yanhua Zhao ZYH-301-1 — Au13C CDCl3 {C:\Bruker\TopSpin3.6.2} 2408 10 - 75 MHz

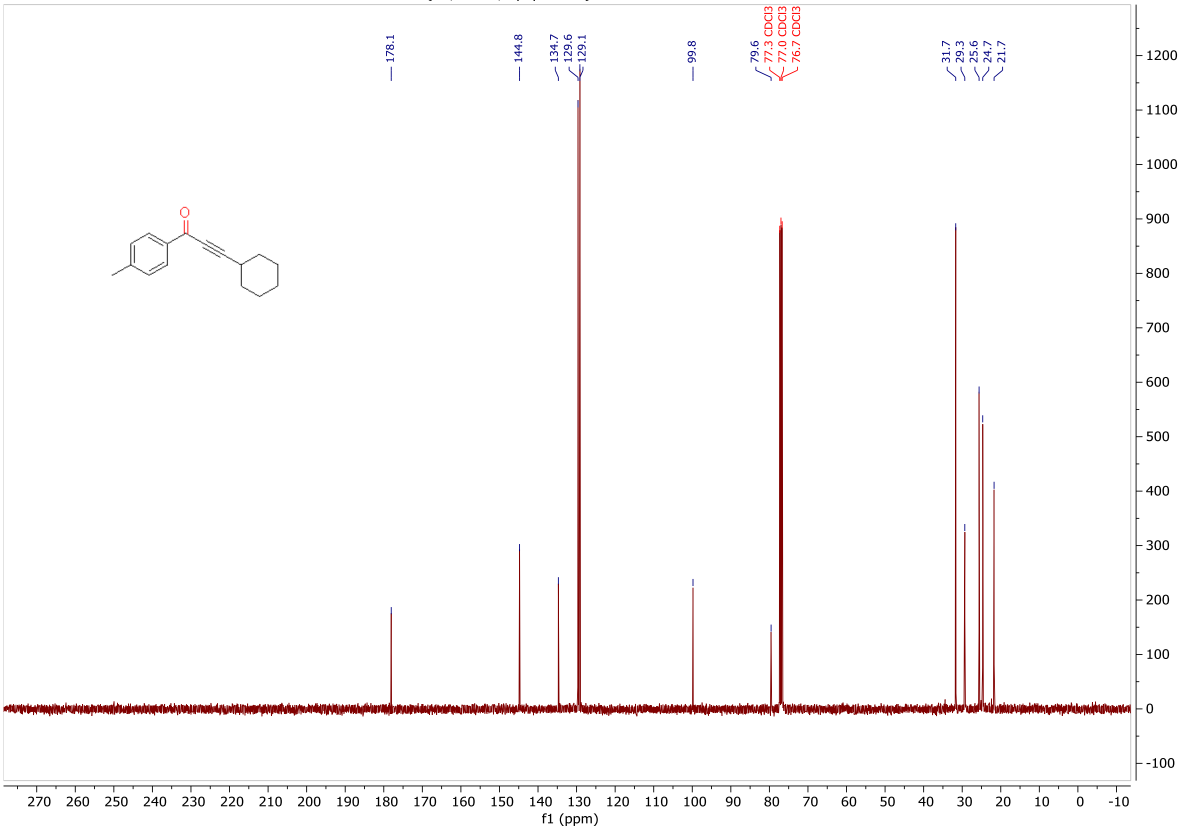

240905.419.10.fid — Yanhua Zhao ZYH-294-14 — Au1H CDCl3 {C:\Bruker\TopSpin3.6.2} 2409 19 - 300 MHz

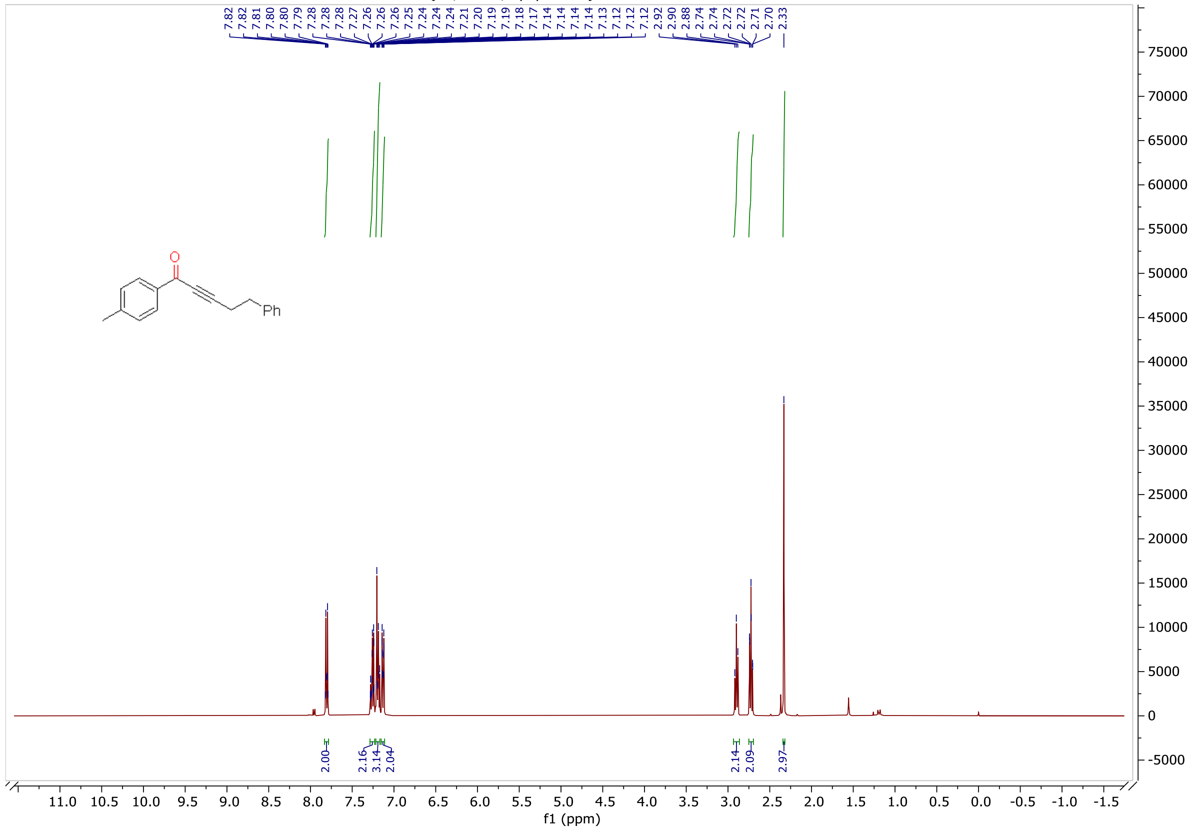

240905.419.11.fid — Yanhua Zhao ZYH-294-14 — Au13C CDCl3 {C:\Bruker\TopSpin3.6.2} 2409 19 - 75 MHz

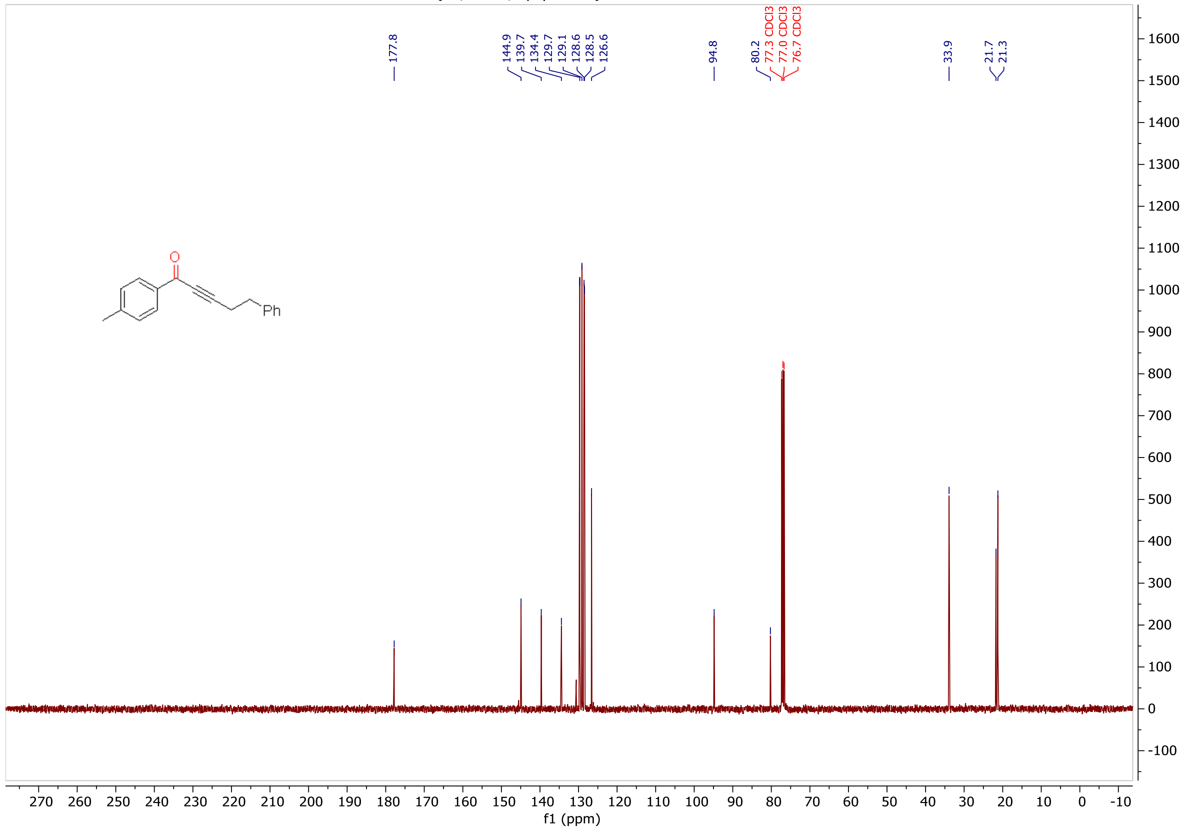

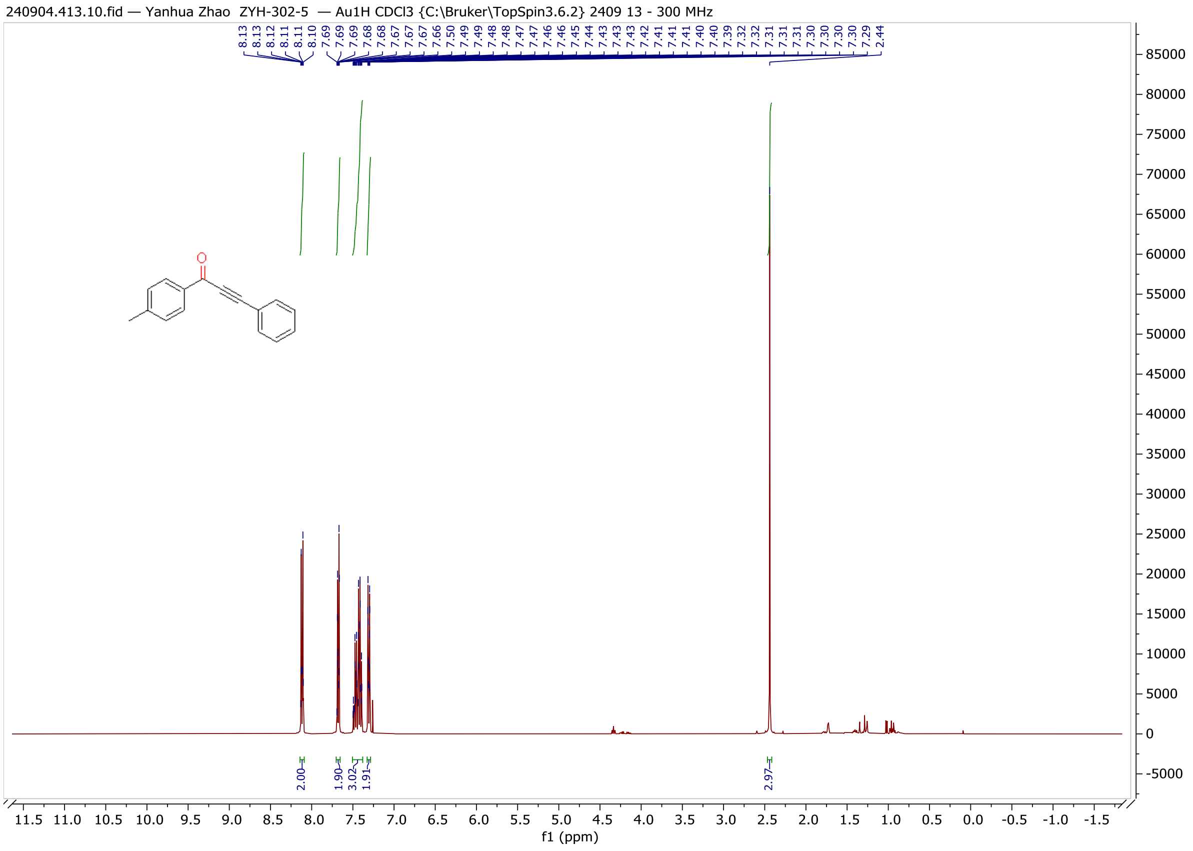

240904.413.10.fid — Yanhua Zhao ZYH-302-5 — Au1H CDCl3 {C:\Bruker\TopSpin3.6.2} 2409 13 - 300 MHz
8.13 8.13 8.12 8.11 8.11 8.10 7.69 7.69 7.69 7.68 7.68 7.67 7.67 7.67 7.66 7.50 7.49 7.49 7.48 7.48 7.47 7.47 7.46 7.46 7.45 7.44 7.43 7.43 7.43 7.42 7.41 7.41 7.41 7.40 7.40 7.39 7.32 7.32 7.31 7.31 7.31 7.30 7.30 7.30 7.29 2.44
O
2.00 1.90 3.02 1.91 2.97
f1 (ppm)

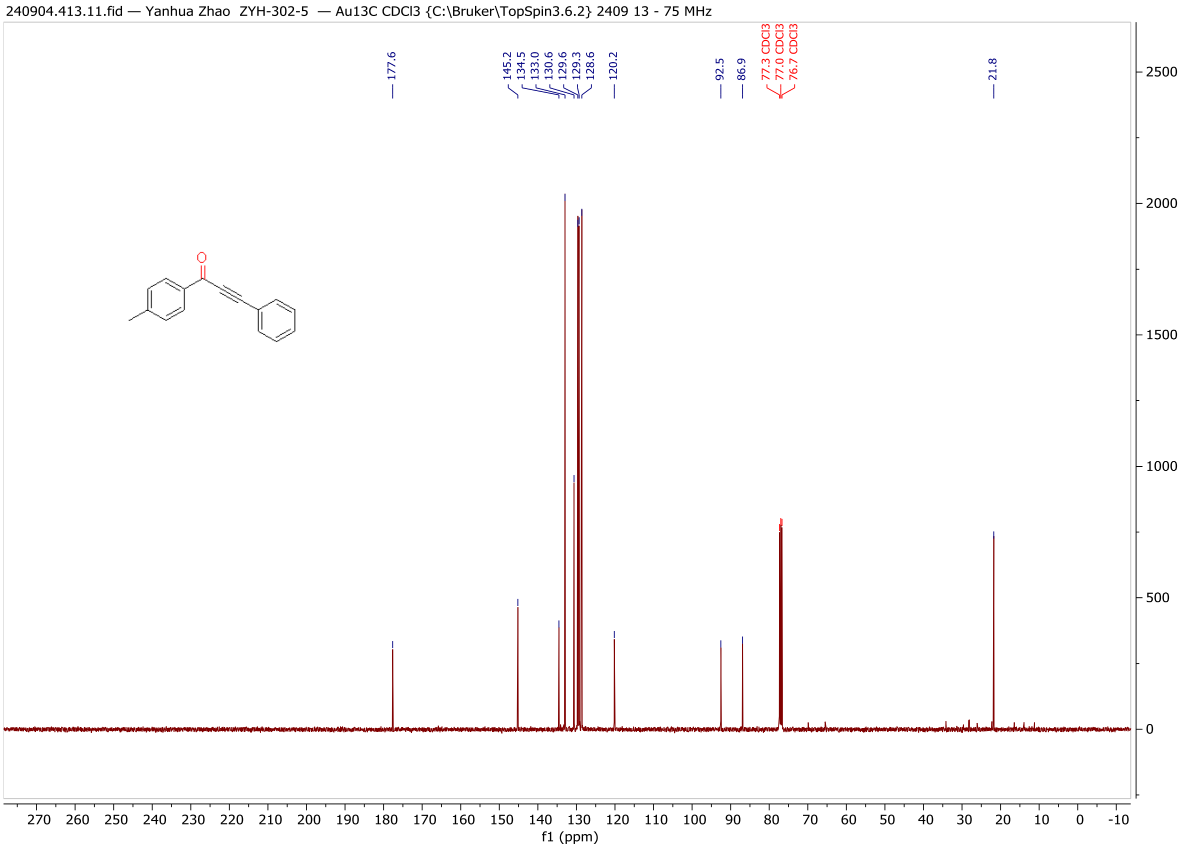

240904.413.11.fid — Yanhua Zhao ZYH-302-5 — Au13C CDCl3 {C:\Bruker\TopSpin3.6.2} 2409 13 - 75 MHz
177.6
145.2 134.5 133.0 130.6 129.6 129.3 128.6
120.2
92.5
86.9
77.3 CDCl3 77.0 CDCl3 76.7 CDCl3
21.8
O
f1 (ppm)

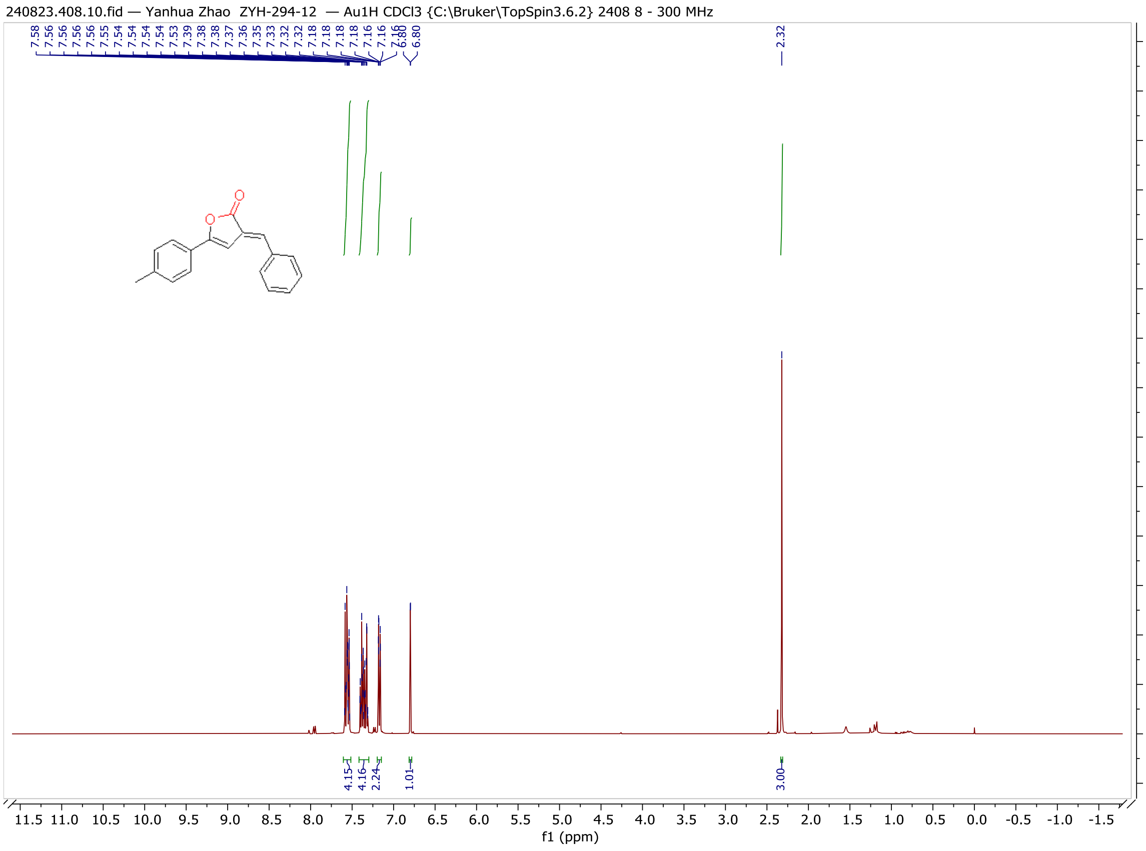
240823.408.10.fid — Yanhua Zhao ZYH-294-12 — Au1H CDCl3 {C:\Bruker\TopSpin3.6.2} 2408 8 - 300 MHz
7.58 7.56 7.56 7.56 7.56 7.55 7.54 7.54 7.54 7.54 7.53 7.39 7.38 7.38 7.38 7.37 7.36 7.35 7.33 7.32 7.32 7.32 7.18 7.18 7.18 7.18 7.16 7.16 7.16 6.80 6.80
2.32
O
O
4.15
4.16
2.24
1.01
3.00
f1 (ppm)

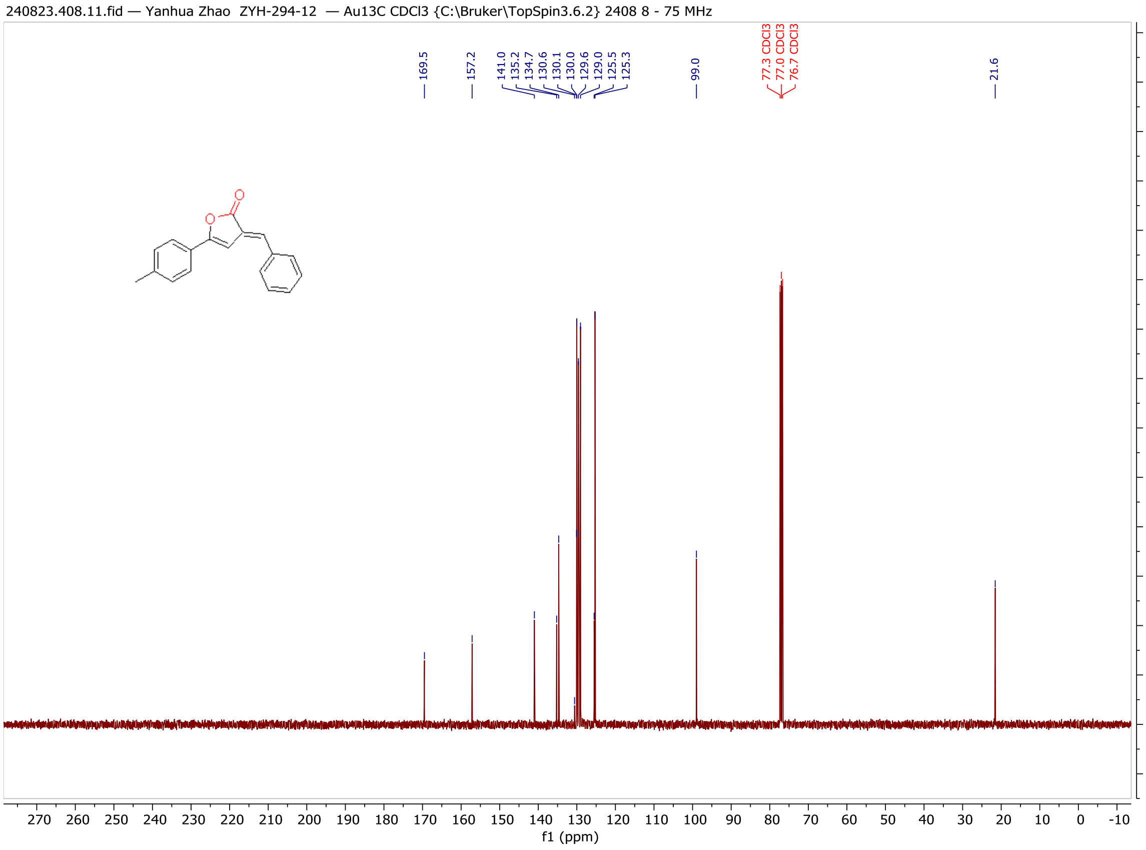
240823.408.11.fid — Yanhua Zhao ZYH-294-12 — Au13C CDCl3 {C:\Bruker\TopSpin3.6.2} 2408 8 - 75 MHz
169.5
157.2
141.0 135.2 134.7 130.6 130.1 130.0 129.6 129.0 125.5 125.3
99.0
77.3 CDCl3
77.0 CDCl3
76.7 CDCl3
21.6
O
O
f1 (ppm)

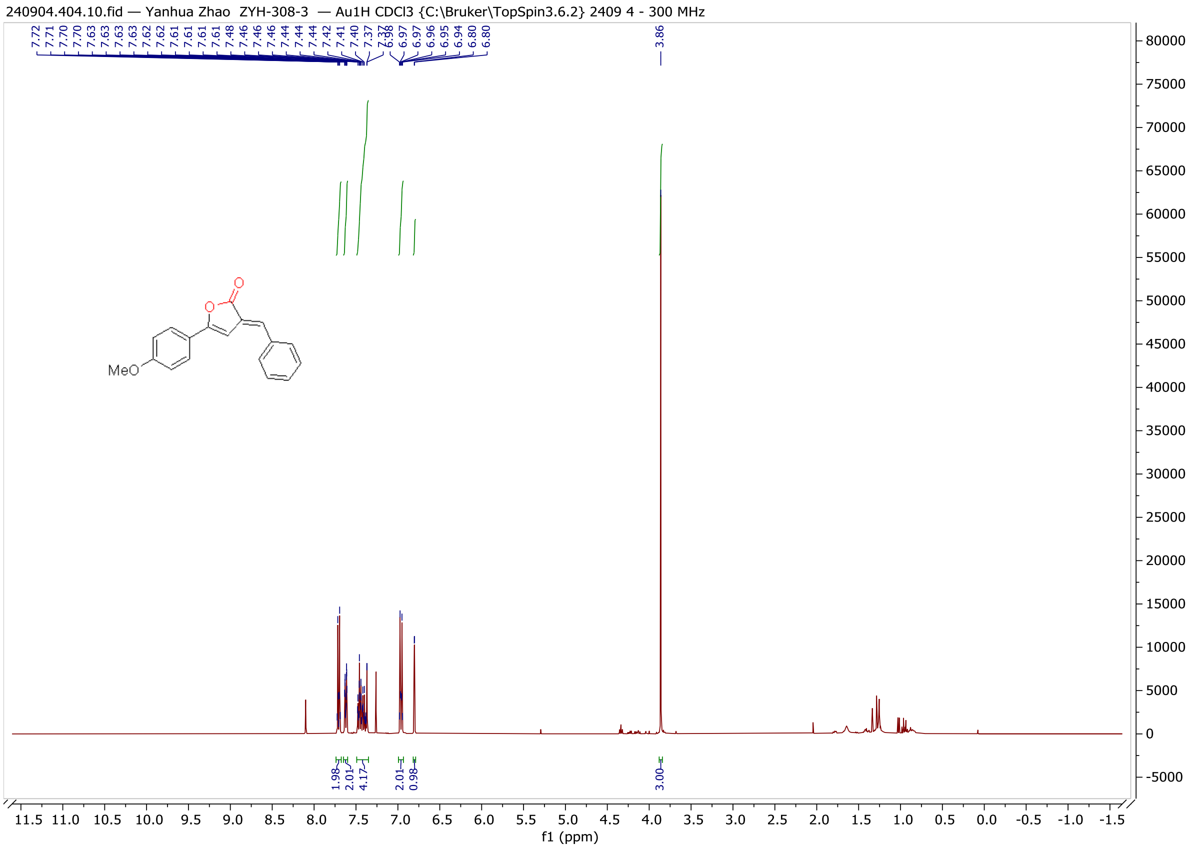
240904.404.10.fid — Yanhua Zhao ZYH-308-3 — Au1H CDCl3 {C:\Bruker\TopSpin3.6.2} 2409 4 - 300 MHz
MeO
3.86
f1 (ppm)

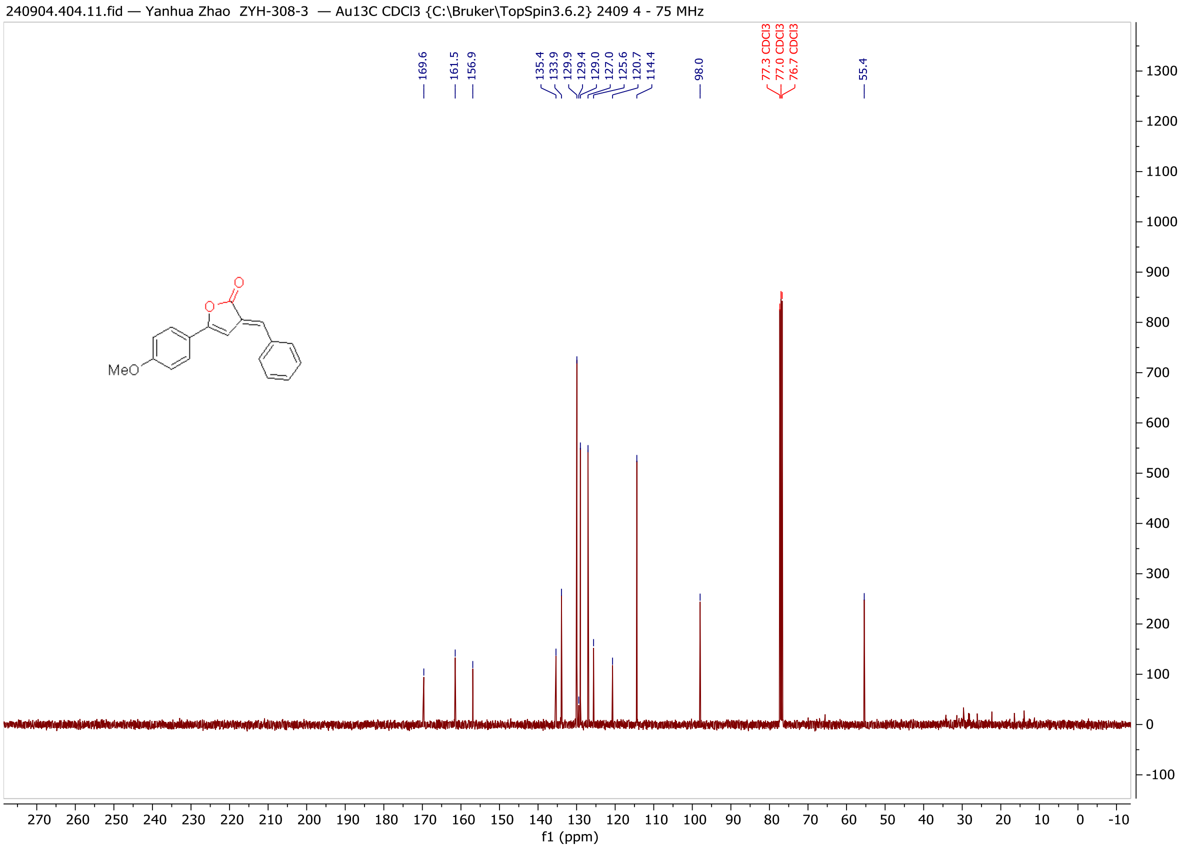
240904.404.11.fid — Yanhua Zhao ZYH-308-3 — Au13C CDCl3 {C:\Bruker\TopSpin3.6.2} 2409 4 - 75 MHz
169.6
161.5
156.9
135.4
133.9
129.9
129.4
129.0
127.0
125.6
120.7
114.4
98.0
77.3 CDCl3
77.0 CDCl3
76.7 CDCl3
55.4
MeO
f1 (ppm)

240904.403.10.fid — Yanhua Zhao ZYH-308-5 — Au1H CDCl3 {C:\Bruker\TopSpin3.6.2} 2409 3 - 300 MHz

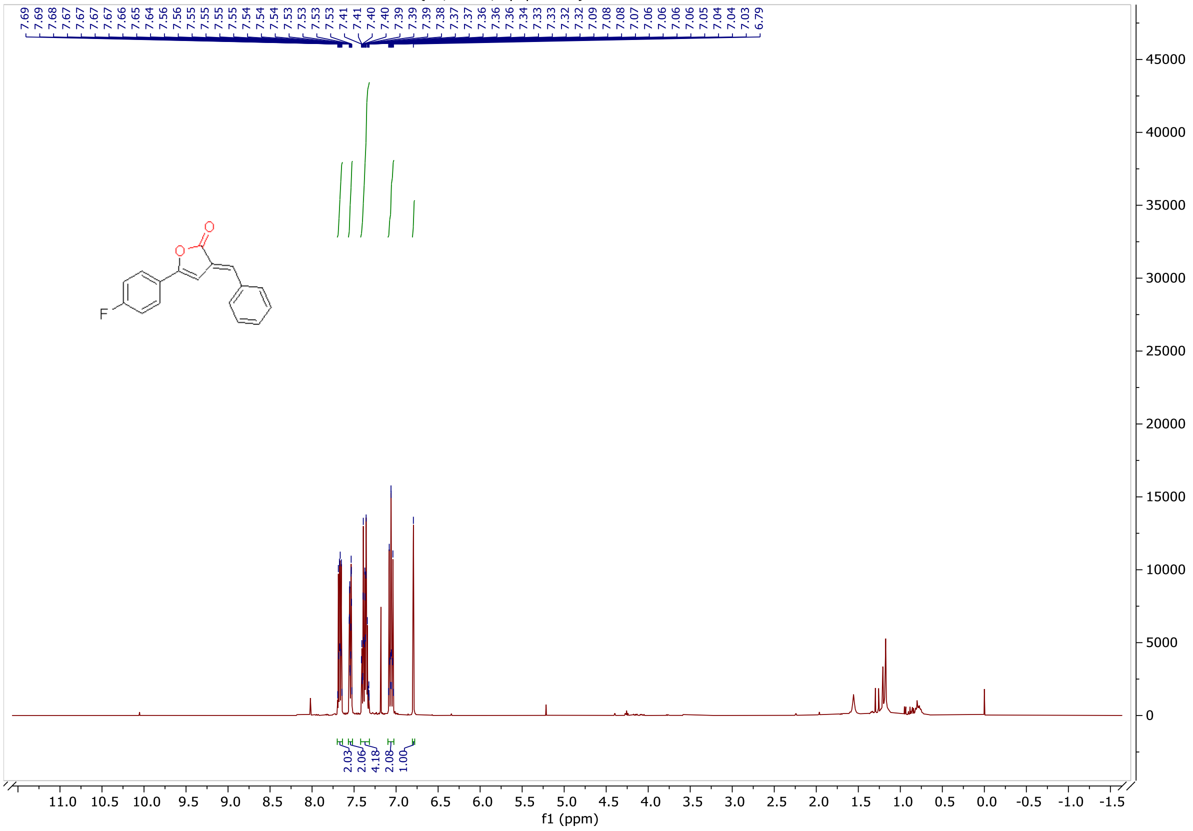

240904.403.11.fid — Yanhua Zhao ZYH-308-5 — Au13C CDCl3 {C:\Bruker\TopSpin3.6.2} 2409 3 - 75 MHz

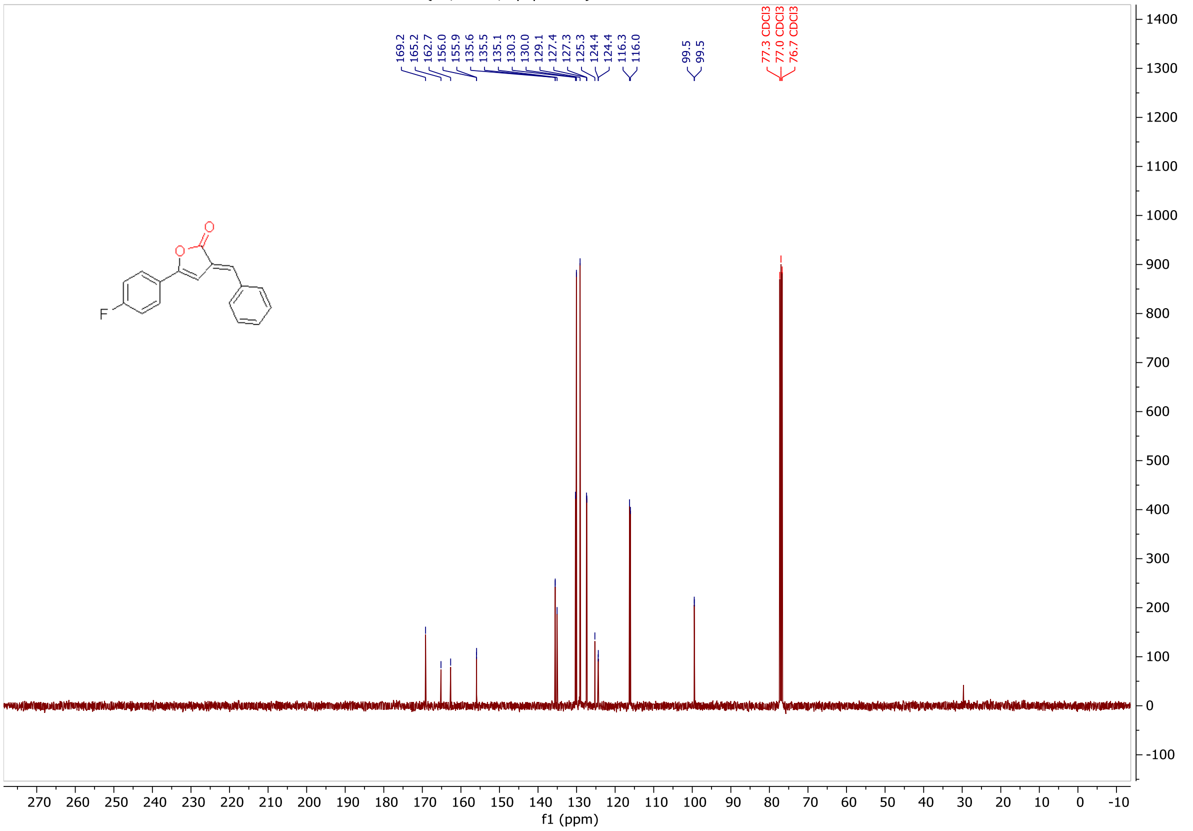

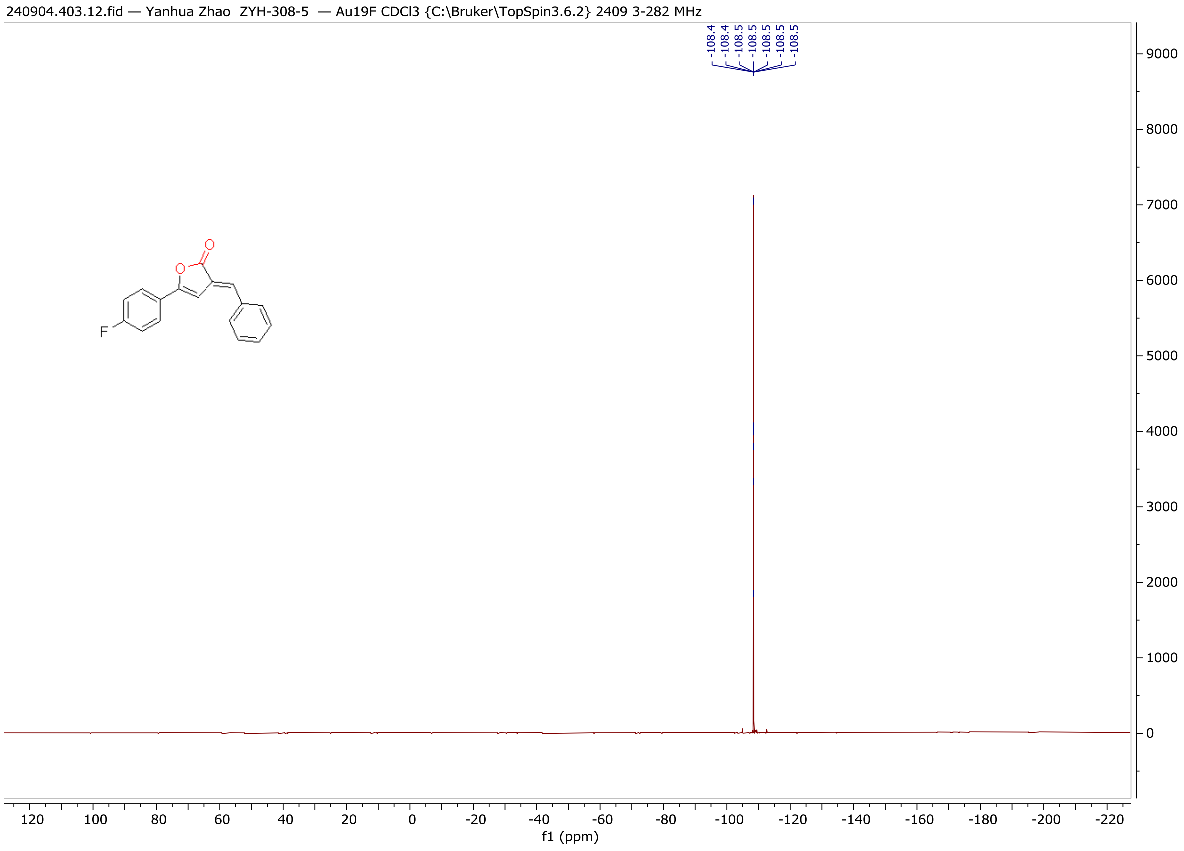

240904.403.12.fid — Yanhua Zhao ZYH-308-5 — Au19F CDCl3 {C:\Bruker\TopSpin3.6.2} 2409 3-282 MHz
-108.4
-108.4
-108.5
-108.5
-108.5
-108.5
-108.5
F
O
O
f1 (ppm)

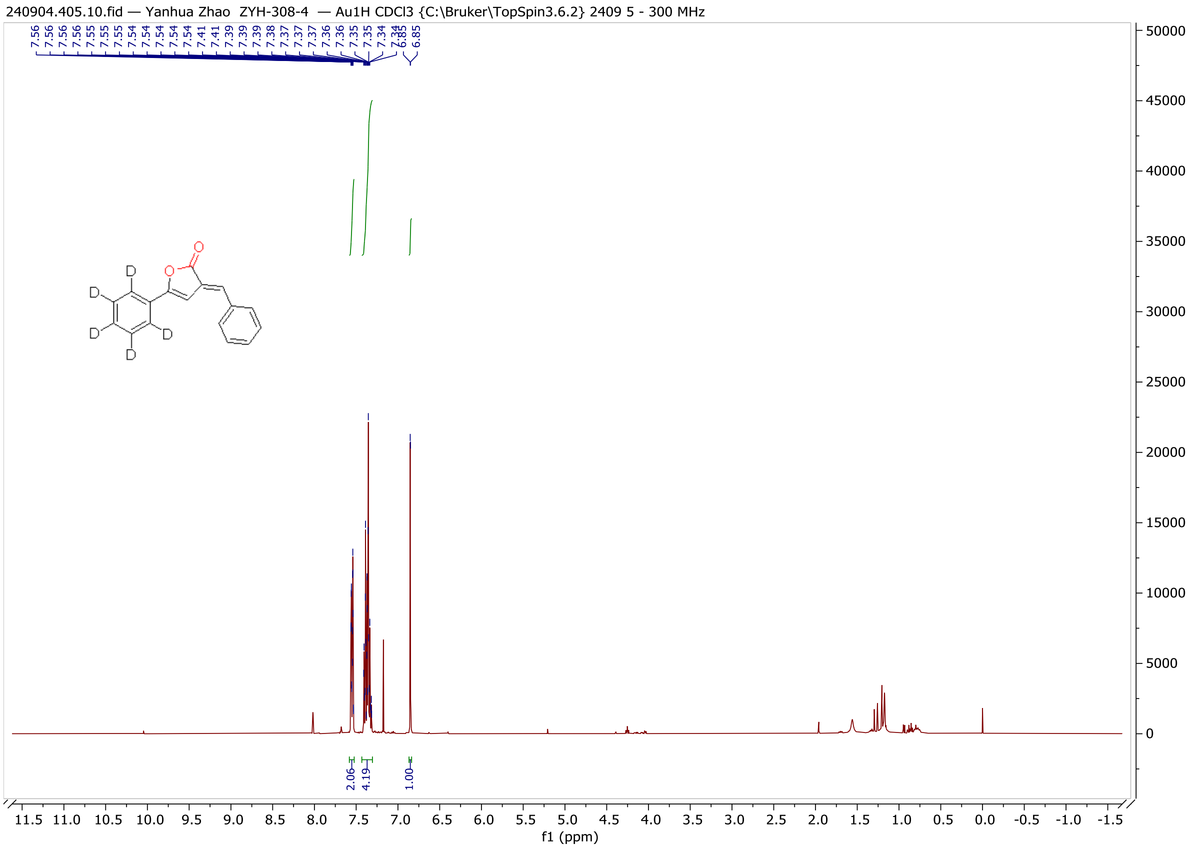
240904.405.10.fid — Yanhua Zhao ZYH-308-4 — Au1H CDCl3 {C:\Bruker\TopSpin3.6.2} 2409 5 - 300 MHz
7.56 7.56 7.56 7.56 7.55 7.55 7.55 7.54 7.54 7.54 7.54 7.54 7.41 7.41 7.39 7.39 7.39 7.38 7.37 7.37 7.37 7.36 7.35 7.35 7.34 7.33 6.85 6.85
D
O
O
2.06
4.19
1.00
f1 (ppm)

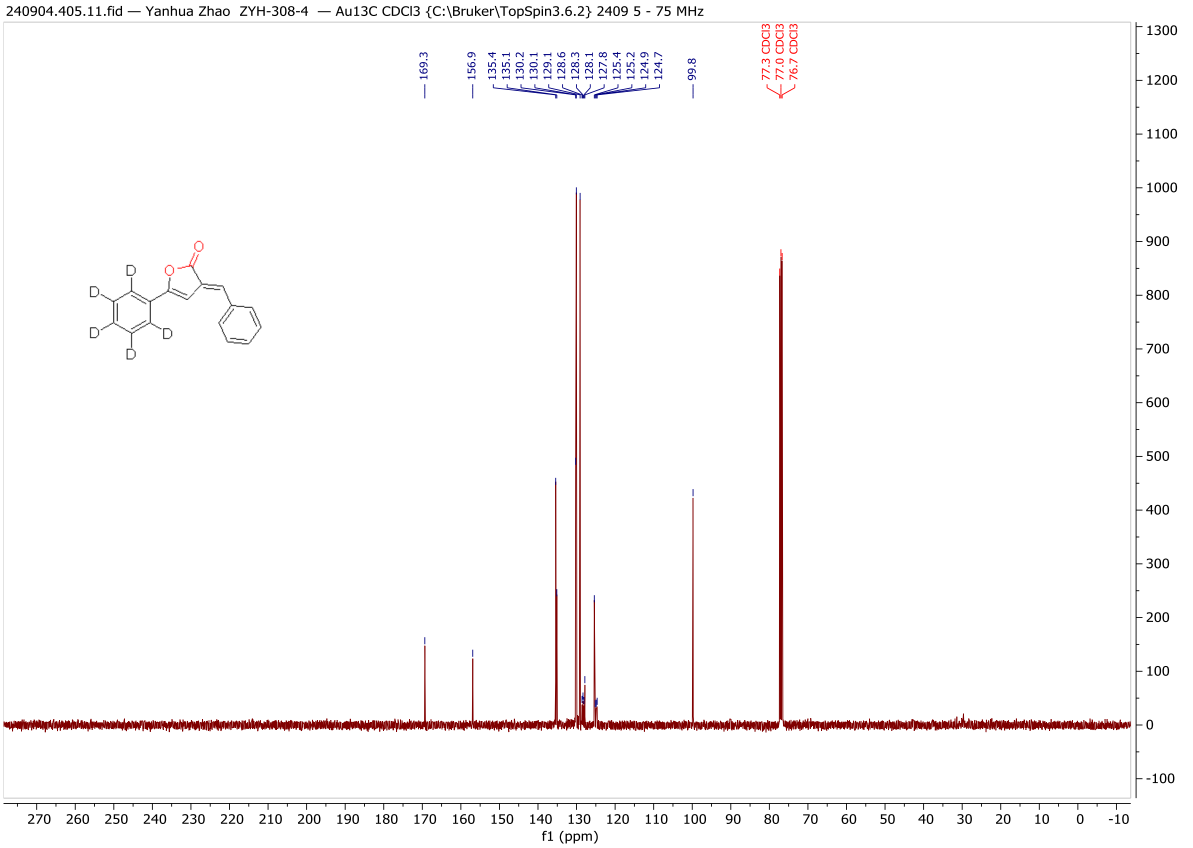
240904.405.11.fid — Yanhua Zhao ZYH-308-4 — Au13C CDCl3 {C:\Bruker\TopSpin3.6.2} 2409 5 - 75 MHz
169.3
156.9
135.4 135.1 130.2 130.1 129.1 128.6 128.3 128.1 127.8 125.4 125.2 124.9 124.7
99.8
77.3 CDCl3 77.0 CDCl3 76.7 CDCl3
D
O
O
f1 (ppm)

240905.418.10.fid — Yanhua Zhao ZYH-308-7 — Au1H CDCl3 {C:\Bruker\TopSpin3.6.2} 2409 18 - 300 MHz

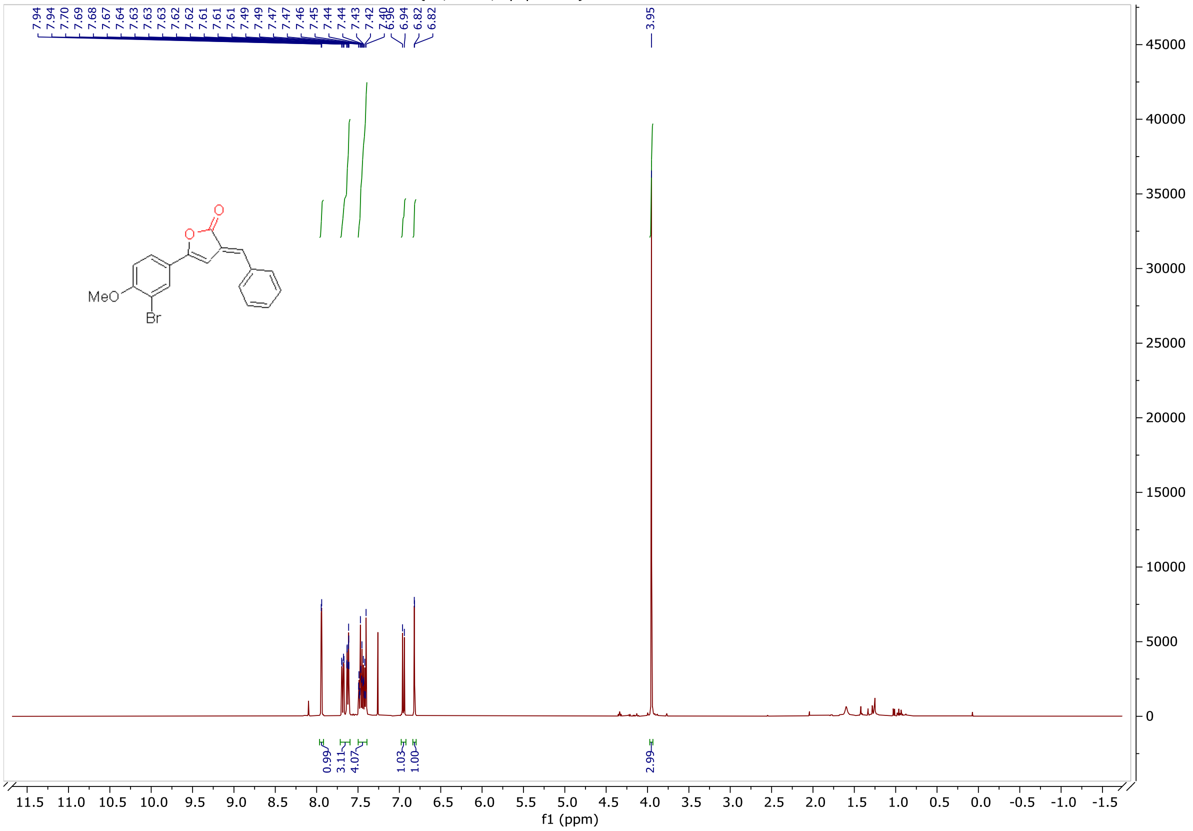

240905.418.11.fid — Yanhua Zhao ZYH-308-7 — Au13C CDCl3 {C:\Bruker\TopSpin3.6.2} 2409 18 - 75 MHz

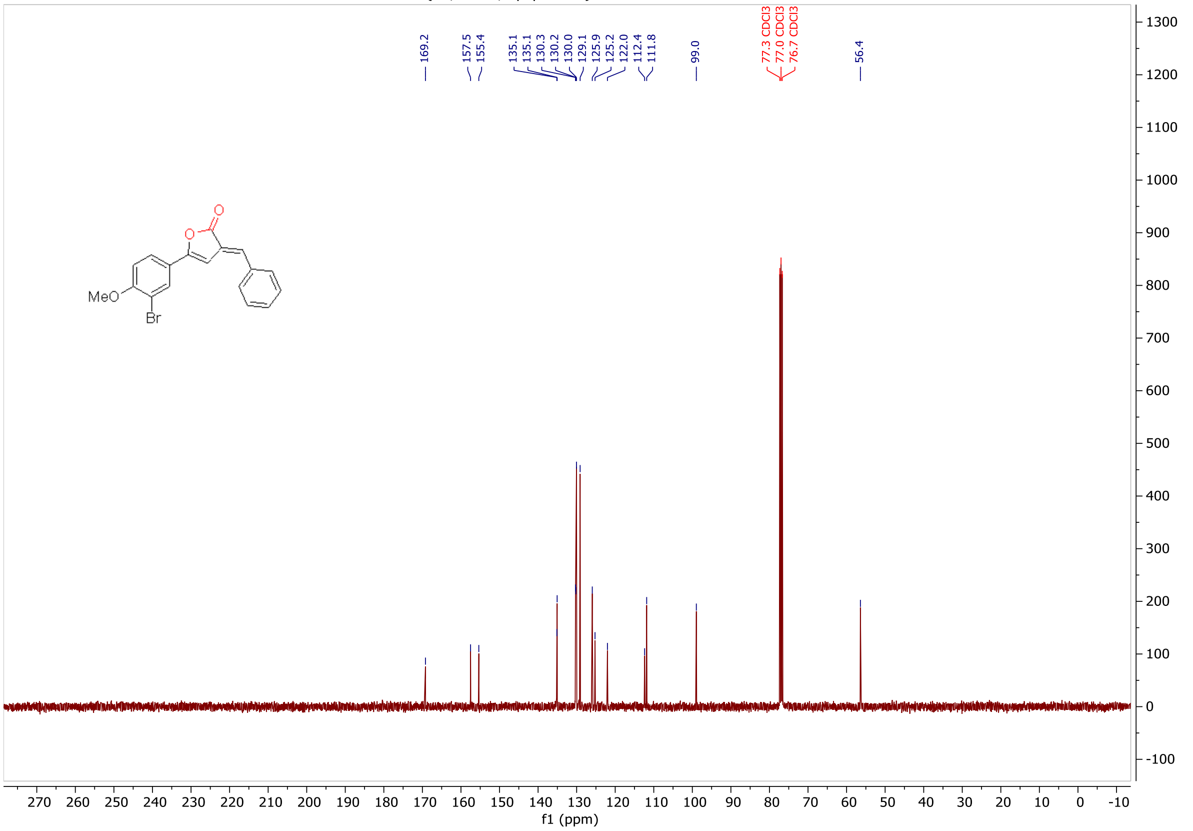

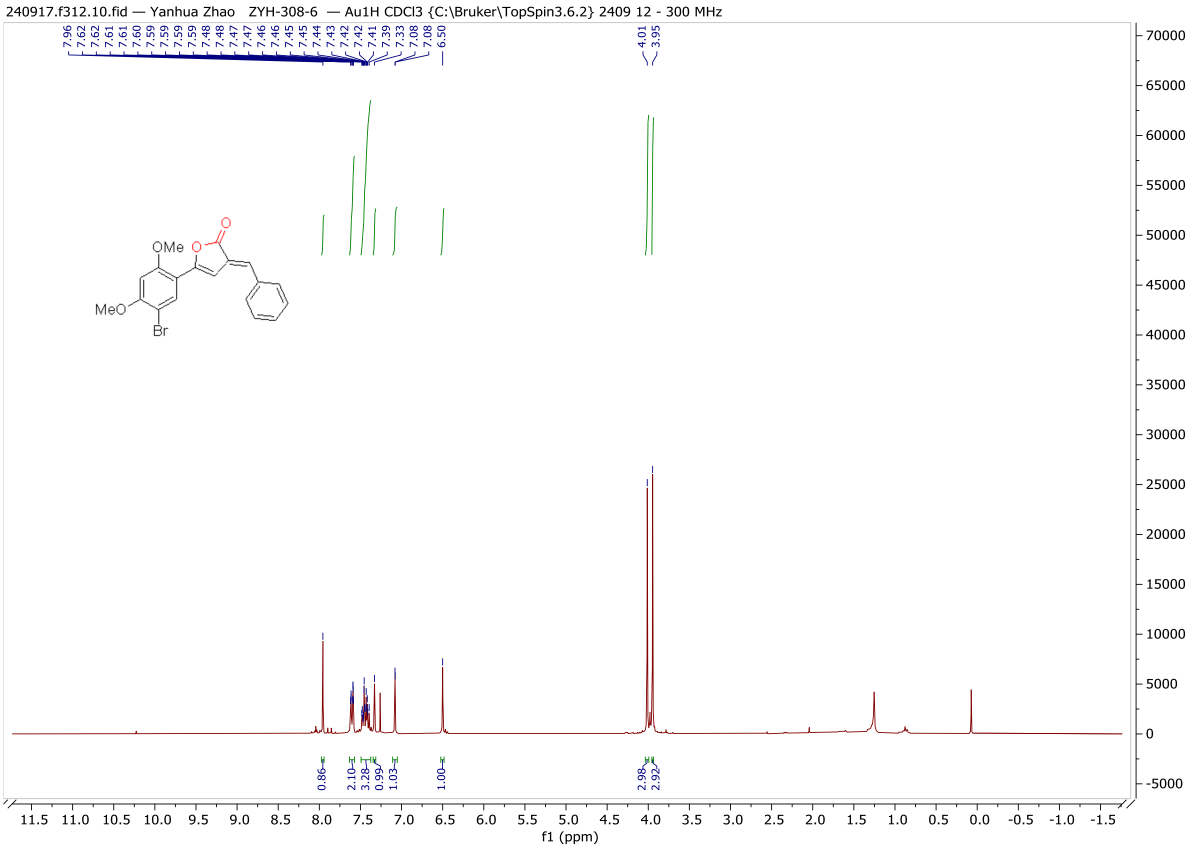
240917.f312.10.fid — Yanhua Zhao ZYH-308-6 — Au1H CDCl3 {C:\Bruker\TopSpin3.6.2} 2409 12 - 300 MHz
f1 (ppm)

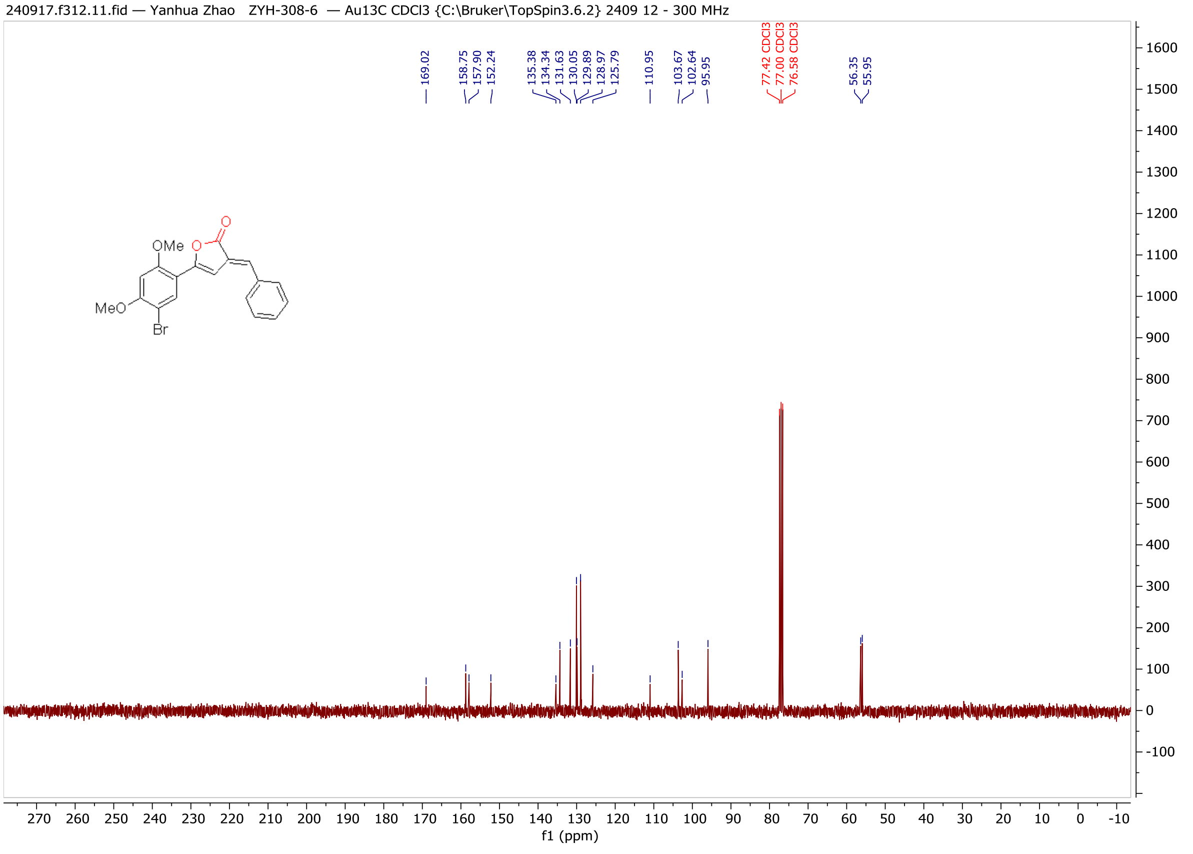
240917.f312.11.fid — Yanhua Zhao ZYH-308-6 — Au13C CDCl3 {C:\Bruker\TopSpin3.6.2} 2409 12 - 300 MHz
f1 (ppm)
